# Synapse-type-specific competitive Hebbian learning forms functional recurrent networks

**DOI:** 10.1073/pnas.2305326121

**Published:** 2024-06-13

**Authors:** Samuel Eckmann, Edward James Young, Julijana Gjorgjieva

**Affiliations:** ^a^Computation in Neural Circuits Group, Max Planck Institute for Brain Research, Frankfurt am Main 60438, Germany; ^b^Computational and Biological Learning Lab, Department of Engineering, University of Cambridge, Cambridge CB2 1PZ, United Kingdom; ^c^School of Life Sciences, Technical University Munich, Freising 85354, Germany

**Keywords:** synaptic plasticity, recurrent networks, excitation–inhibition balance, surround suppression

## Abstract

Cortical networks exhibit complex stimulus–response patterns that are based on specific recurrent interactions between neurons. For example, the balance between excitatory and inhibitory currents has been identified as a central component of cortical computations. However, it remains unclear how the required synaptic connectivity can emerge in developing circuits where synapses between excitatory and inhibitory neurons are simultaneously plastic. Using theory and modeling, we propose that a wide range of cortical response properties can arise from a single plasticity paradigm that acts simultaneously at all excitatory and inhibitory connections—Hebbian learning that is stabilized by the synapse-type-specific competition for a limited supply of synaptic resources. In plastic recurrent circuits, this competition enables the formation and decorrelation of inhibition-balanced receptive fields. Networks develop an assembly structure with stronger synaptic connections between similarly tuned excitatory and inhibitory neurons and exhibit response normalization and orientation-specific center-surround suppression, reflecting the stimulus statistics during training. These results demonstrate how neurons can self-organize into functional networks and suggest an essential role for synapse-type-specific competitive learning in the development of cortical circuits.

## Significance

Cortical circuits perform diverse computations, primarily determined by highly structured synaptic connectivity patterns that develop during early sensory experience via synaptic plasticity. To understand how these structured connectivity patterns emerge, we introduce a general learning framework for networks of recurrently connected neurons. The framework is rooted in the biologically plausible assumption that synapses compete for limited synaptic resources, which stabilizes synaptic growth. Motivated by the unique protein composition of different synapse types, we assume that different synapse types compete for separate resource pools. Using theory and simulation, we show how this synapse-type-specific competition allows the stable development of structured synaptic connectivity patterns, as well as diverse computations like response normalization and surround suppression.

Computation in neural circuits is based on the interactions between recurrently connected excitatory (E) and inhibitory (I) neurons (1–4). In sensory cortices, response normalization, surround and gain modulation, predictive processing, and attention all critically involve inhibitory neurons (5–10). Theoretical work has highlighted the experimentally observed balance of stimulus selective excitatory and inhibitory input currents as a critical requirement for many neural computations (11–16). For example, recent models based on balanced E–I networks can explain a wide range of cortical phenomena, such as cross-orientation and surround suppression (17, 18), as well as stimulus-induced neural variability (19–21). A major caveat of these models is that the network connectivity is usually static and designed by hand, albeit based on experimental measurements. In contrast, in the brain, synapses are plastic and adjust to the statistics of sensory inputs. How synaptic weights self-organize in a biologically plausible manner to generate many of the nonlinear response properties observed experimentally is not well understood. Earlier theoretical work on inhibitory plasticity has focused on the balance of excitation and inhibition in single neurons (22–24), but has not been able to explain the development of inhibition-balanced receptive fields when excitatory and inhibitory inputs are both plastic. In more recent recurrent network models, only a fraction of excitatory and inhibitory synapse-types are modeled as plastic and neural responses exhibit a narrow subset of the different response patterns recorded in experiments (14, 25–29).

Here, we present a Hebbian learning framework with minimal assumptions that explains a wide range of experimental observations. Our framework is based on two key properties: First, all synaptic strengths evolve according to a Hebbian plasticity rule that is stabilized by the competition for a limited supply of synaptic resources (30–33). Second, motivated by the unique protein composition of excitatory and inhibitory synapses, different synapse types compete for separate resource pools. Building on classical work on Hebbian plasticity (30, 31), we develop an analytical framework that provides an intuitive understanding of the weight dynamics in recurrent networks of excitatory and inhibitory neurons. In numerical simulations, we reveal how the synapse-type-specific competition for resources enables the self-organization of neurons into functional networks. Beyond the formation of inhibition-balanced feedforward receptive fields, we demonstrate that emergent recurrent connectivity can generate a wide range of computations observed in cortical circuits.

## Results

### Synapse-Type-Specific Plasticity Enables the Joint Development of Stimulus Selectivity and E–I Balance.

**Fig. 1.**
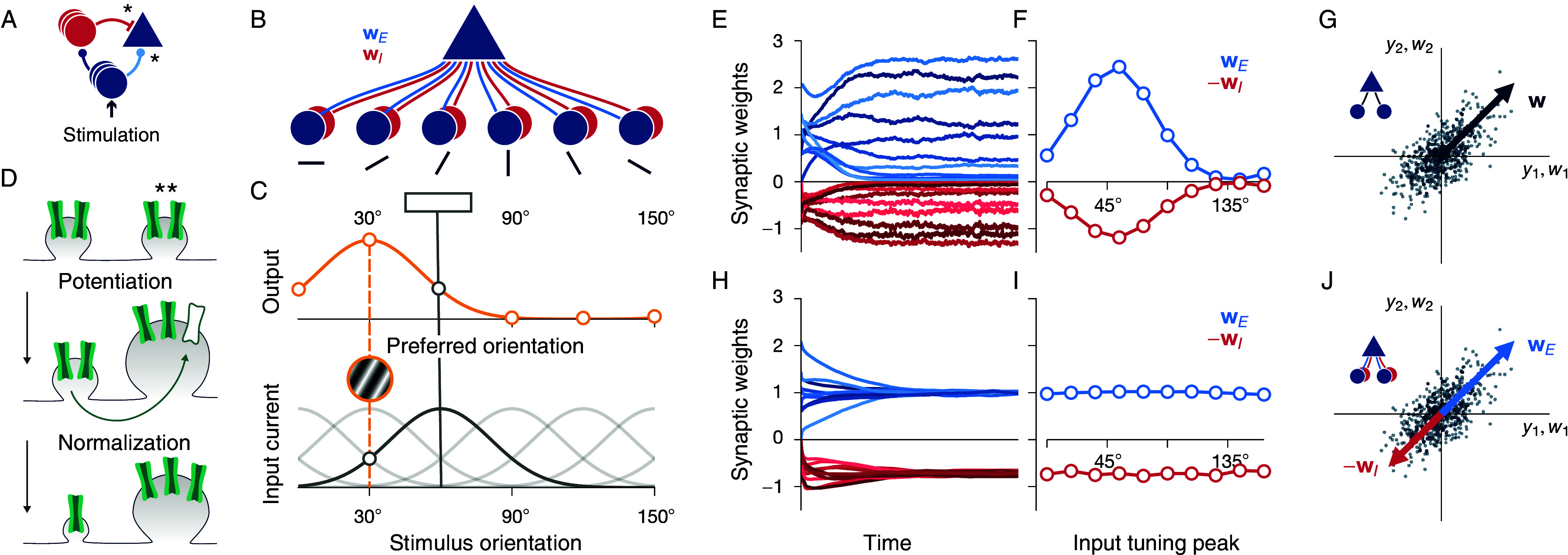
Synapse-type-specific competitive Hebbian learning enables the development of stimulus selectivity and inhibitory balance. (*A*) Feedforward input to a model pyramidal neuron (blue triangle) during stimulation. The neuron receives direct excitation (light blue) and disynaptic inhibition (red). Plastic synapses are marked by *. (*B*) A single postsynaptic pyramidal neuron receives synaptic input from a population of excitatory ($w_{E}$), and a population of inhibitory ($w_{I}$) neurons. (*C*) Excitatory and inhibitory input neurons are equally tuned to the orientation of a stimulus grating (bottom, tuning curve of neurons tuned to 60^°^ highlighted in dark gray) and exhibit a Gaussian-shaped population response (orange, solid line) when a single grating of 30^°^ is presented (orange plate, dashed line). (*D*) Hebbian potentiation of a synapse (**) is normalized due to a limited amount of synaptic resources in the dendritic branch, here reflected by a fixed number of synaptic channels (green). (*E*) Weight convergence of synapses of the feedforward circuit in *B*, where excitatory (blue) and inhibitory (red) weights are plastic according to synapse-type-specific competitive Hebbian learning. All synaptic weights were initialized randomly. (*F*) Final synaptic weight strengths, after training, as a function of the tuning peak of the corresponding presynaptic neurons. (*G*) Excitatory synaptic weight vector (blue arrow) of a single pyramidal neuron with linear activation function. The pyramidal neuron receives input from two excitatory neurons ($y_{1}$ and $y_{2}$, compare *Inset*). Each dot corresponds to one input pattern. After training, the weight vector aligns with the direction of maximum variance, which corresponds to the principal eigenvector of the input covariance matrix. (*H* and *I*) Same as in *E* and *F*, but for classic inhibitory plasticity. The development of stimulus selectivity is prevented by fast inhibitory plasticity. (*J*) Excitatory (blue) and inhibitory (red) synaptic weight vectors of a single pyramidal neuron with linear activation function. The pyramidal neuron receives input from two pairs of excitatory and inhibitory neurons ($y_{1}$ and $y_{2}$, compare *Inset*). Each excitatory-inhibitory input pair has identical firing activities $y_{i}$. After training via synapse-type-specific competitive Hebbian learning, the excitatory and inhibitory weight vectors both align with the principal component, i.e., excitatory and inhibitory synaptic weights are balanced.

To understand plasticity in recurrently connected E–I networks, we considered simplified circuits of increasing complexity. We first asked how E–I balance and stimulus selectivity can simultaneously develop in a single neuron. The neuron receives input from an upstream population of excitatory neurons, and disynaptic inhibitory input from a population of laterally connected inhibitory neurons that themselves receive input from the same upstream population (Fig. 1*A*). We studied the self-organization of excitatory and inhibitory synapses that project onto the single postsynaptic neuron (Fig. 1*B*), assuming that input synapses that project onto inhibitory neurons remained fixed (Fig. 1*A*). Following experimental results (34–37), we assumed that inhibitory and excitatory input neurons are equally selective for the orientation of a stimulus grating (Fig. 1*C*, *Bottom*). We presented uniformly distributed oriented stimuli to the network in random order. Stimuli elicited a Gaussian-shaped response in the population of input neurons (Fig. 1*C*, *Top*) and thus drove the postsynaptic neuron (see *Materials and Methods* for details). Synapses are plastic according to a basic Hebbian rule:

[1] $$\begin{array}{c} \Delta w_{A}\propto y_{A}r,\;A\in \left\{E,I\right\}, \end{array}$$

where r is the postsynaptic firing rate, $y_{A}$ is a vector that holds the presynaptic firing rates of excitatory (A=E) and inhibitory (A=I) neurons, and $\Delta w_{A}$ are the corresponding synaptic weight changes. Experimental results have shown that after the induction of long-term plasticity neither the total excitatory nor the total inhibitory synaptic area change (32). This suggests that a synapse can only grow at the expense of another synapse—a competitive mechanism potentially mediated by the limited supply of synaptic proteins (Fig. 1*D*) (33). Motivated by these results, we adopted a competitive normalization rule for both excitatory and inhibitory synapses:

[2] $$\begin{array}{c} w_{A}\leftarrow W_{A}\frac{w_{A}+\Delta w_{A}}{\|w_{A}+\Delta w_{A}\|}, \end{array}$$

where A∈{E,I}, and $W_{E}$, $W_{I}$ are the maintained total excitatory and inhibitory synaptic weight, respectively. Shortly after random initialization, excitatory and inhibitory weights stabilize (Fig. 1*E*) and form aligned, Gaussian-shaped tuning curves (Fig. 1*F*) that reflect the shape of the input stimuli (Fig. 1*C*). As a result, neural responses become orientation selective while inhibitory and excitatory inputs are equally tuned, which demonstrates the joint development of stimulus selectivity and E–I balance.

### Excitatory Plasticity Performs Principal Component Analysis.

To uncover the principles of synapse-type-specific competitive Hebbian learning, we analyzed the feedforward model analytically. It is well established that in the absence of inhibition, competitive Hebbian learning rules generate stimulus selective excitatory receptive fields (30, 31). In the case of a linear activation function, $r\propto u\equiv w^{⊺}y$, the expected total synaptic efficacy changes can be expressed as (31):

[3] $$\begin{array}{cc} \left\langle {\dot{w}}_{E}\right\rangle & \propto Cw_{E}-\gamma w_{E}, \end{array}$$

were $C=\langle \;y_{E}y_{E}^{⊺}\rangle$ is the input covariance matrix, with ⟨·⟩ being the temporal average, and γ is a scalar normalization factor that regulates Hebbian growth. Then, fixed points, for which $\langle {\dot{w}}_{E}\rangle =0$, are eigenvectors of the covariance matrix. The neuron becomes selective to the first principal component of its input data, i.e., the fixed point input weight vector aligns with the input space direction of maximum variance (30, 31) (Fig. 1*G*; see *SI Appendix*, section 1.2 for details). For nonlinear activation functions r=f(u), neurons become selective for higher-order correlations, e.g., independent components, in their inputs (38, 39). Such learning rules have been shown to result in feedforward receptive fields that resemble simple cell receptive fields in the visual cortex (40, 41). In the following, we call the fixed points of such pure feedforward circuits ‘input modes’. This entails principal components, in the case of linear activation functions, and more complex, e.g., simple-cell-like, receptive fields in the case of nonlinear activation functions.

### Classic Inhibitory Plasticity Prevents Stimulus Selectivity.

We next examined how inhibitory plasticity affects the development of stimulus selectivity. Previous work has suggested that inhibitory synaptic plasticity in the cortex is Hebbian (42, 43) and imposes a target firing rate $r_{0}$ on the postsynaptic neuron (23):

[4] $$\begin{array}{c} \left\langle {\dot{w}}_{I}\right\rangle \propto \left\langle y_{I}\left(r-r_{0}\right)\right\rangle , \end{array}$$

where synaptic change becomes zero when the postsynaptic firing rate r is equal to the target rate $r_{0}$. With this “classic” inhibitory plasticity rule, inhibitory synaptic weight growth is unbounded. However, since an increase of inhibitory synaptic weights usually entails a decrease in postsynaptic firing rate r, the plasticity rule is self-limiting and synaptic weights stop growing once the target firing rate $r_{0}$ is reached. When excitatory synaptic weights remain fixed, classic inhibitory plasticity leads to balanced excitatory and inhibitory input currents (23). However, when excitatory synaptic weights are also plastic, neurons develop no stimulus selectivity (24): classic inhibitory plasticity must act on a faster timescale than excitatory plasticity to maintain stability (24). Then the postsynaptic target firing rate is consistently met and average excitatory synaptic weight changes only differ among each other due to different average presynaptic firing rates, which prevents the development of stimulus selectivity (Fig. 1*H* and *I*; see *SI Appendix*, section 1.2.3 for details).

### Synapse-Type-Specific Competition Enables Balanced Principal Component Analysis.

Synapse-type-specific competitive Hebbian learning (Eqs. **1** and **2**) enables the joint development of stimulus selectivity and balanced input currents. In contrast to classic inhibitory plasticity, under synapse-type-specific competitive Hebbian learning, inhibitory synaptic growth is not stabilized by a target firing rate. Instead, as excitatory synapses, inhibitory synapses compete for a limited supply of synaptic resources that maintain the total amount of synaptic strength. As we did for excitatory synapses (Eq. **3**), we incorporated the normalization step (Eq. **2**) into the update rule (Eq. **1**) and considered the simpler case of a linear activation function f(u)∝u:

[5] $$\begin{array}{c} \left\langle \dot{\overset{¯}{w}}\right\rangle \propto \overset{¯}{C}\overset{¯}{w}-\gamma \left(\begin{array}{c} w_{E}\\ 0 \end{array}\right)-\rho \left(\begin{array}{c} 0\\ w_{I} \end{array}\right), \end{array}$$

[6] $$\begin{array}{c} \overset{¯}{w}=\left(\begin{array}{c} w_{E}\\ w_{I} \end{array}\right),\;\overset{¯}{C}\equiv \langle \left(\begin{array}{c} y_{E}y_{E}^{⊺}-y_{E}y_{I}^{⊺}\\ y_{I}y_{E}^{⊺}-y_{I}y_{I}^{⊺} \end{array}\right)\rangle , \end{array}$$

where γ and ρ are scalars that ensure normalization of excitatory and inhibitory weights, respectively. In addition, we defined the modified covariance matrix $\overset{¯}{C}$. Then multiples of the excitatory and the inhibitory part of the eigenvectors of the modified covariance matrix $\overset{¯}{C}$ are fixed points of the weight dynamics (see *SI Appendix*, section 2 for details). When excitatory and inhibitory inputs are equally stimulus selective, such that one can approximate $y_{E}\propto y_{I}$, the modified covariance matrix $\overset{¯}{C}$ is composed of multiples of the original covariance matrix C (cf. Eq. **6**). This implies that, if excitatory and inhibitory synaptic weights have identical shape, $w_{E}\propto w_{I}$, equal to a multiple of an eigenvector of C, the system is in a fixed point (Fig. 1*J*), where $\langle \dot{\overset{¯}{w}}\rangle =0$ (cf. Eq. **5**). Neurons become selective for activity along one particular input direction, while excitatory and inhibitory neural inputs are cotuned, which explains the joint development of stimulus selectivity and E–I balance in feedforward circuits, in agreement with our numerical simulations with nonlinear activation functions (Fig. 1*E* and *F*).

### Lateral Inputs Shape Feedforward Weight Dynamics.

We wanted to understand how fully plastic recurrent networks of excitatory and inhibitory neurons can self-organize into functional circuits. Therefore, we next investigated the effect of synapse-type-specific competitive Hebbian learning in recurrent networks.

**Fig. 2.**
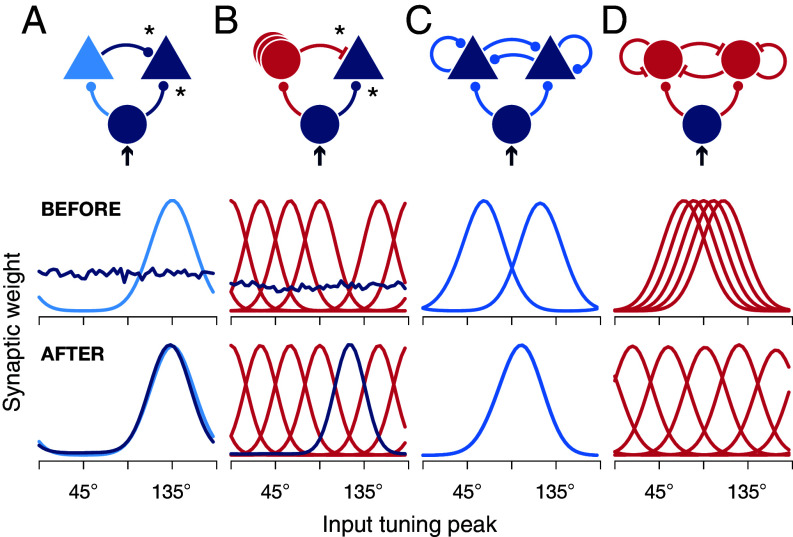
Feedforward tunings are affected by lateral input in microcircuit motifs. (*A*) In addition to feedforward input from a population of orientation-tuned excitatory cells (blue circle), a neuron receives lateral input from an excitatory neuron with fixed feedforward tuning (light blue). * indicates plastic synapses. Feedforward tuning curves of the two neurons are shown before (*Center* row) and after (*Bottom* row) training. (*B*) Same as in *A*, for lateral input from multiple inhibitory neurons with fixed feedforward tuning. (*C*) Same as in *A*, for two recurrently connected excitatory neurons with all feedforward and recurrent synapses plastic. (*D*) Same as in *C*, for inhibitory neurons. All synapses plastic.

In a first step, we considered how lateral input from an excitatory neuron with fixed selectivity for a specific feedforward input mode affects synaptic weight dynamics in a simple microcircuit motif (Fig. 2*A*, *Top*). We observed that a downstream neuron becomes preferentially tuned to the feedforward input mode of the lateral projecting neuron (Fig. 2*A*, *Bottom*; cf. *SI Appendix*, section 3). Similarly, laterally projecting inhibitory neurons repel downstream neurons from their input modes (Fig. 2*B*). However, when two excitatory neurons are reciprocally connected, they pull each other toward their respective input modes, and their tuning curves and activities become correlated (Fig. 2*C*). This contradicts experimental observations that brain activity decorrelates over development (44, 45). In line with these results, in our model, interconnected inhibitory neurons repel each other and their tuning curves decorrelate (Fig. 2*D*).

### Tuning Curve Decorrelation in Fully Plastic Recurrent E–I Networks.

**Fig. 3.**
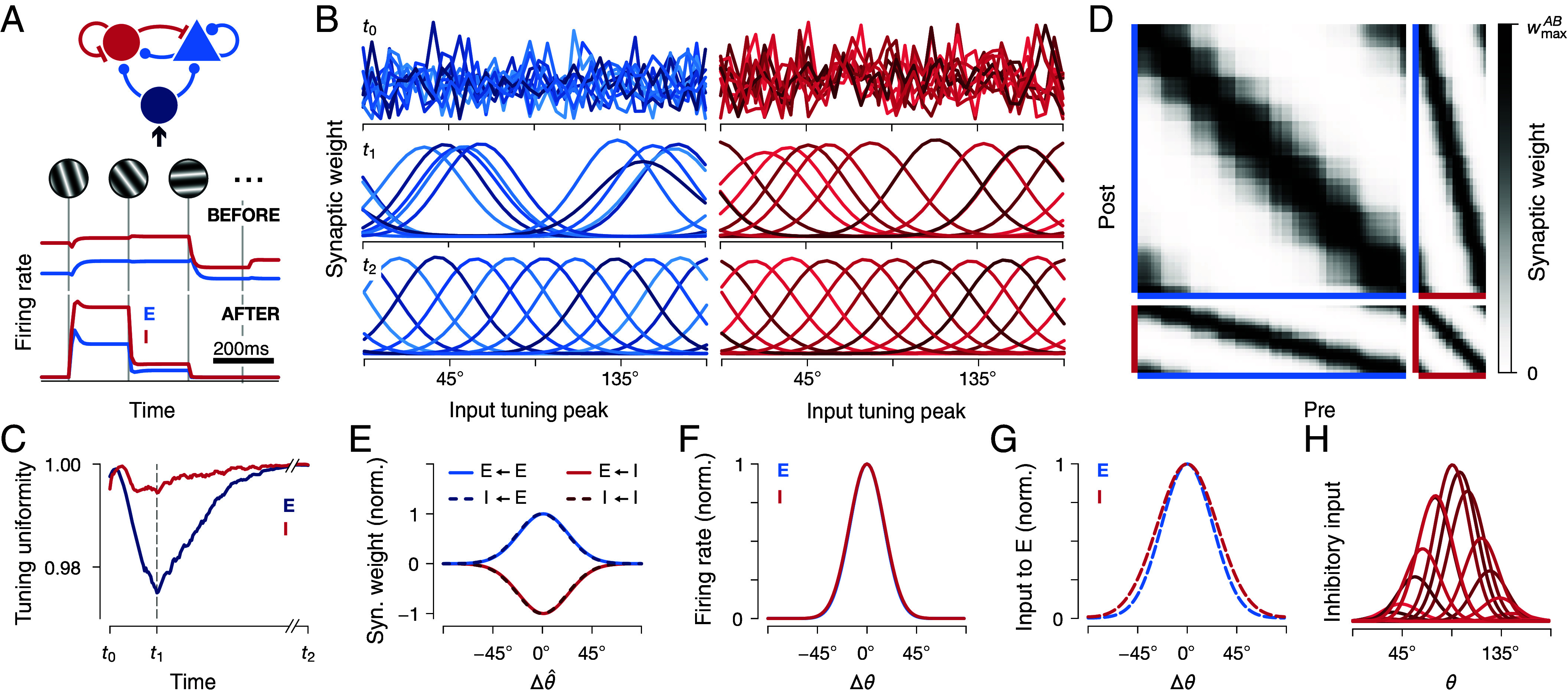
Tuning curve decorrelation in plastic recurrent networks. (*A*) *Top*: A population of recurrently connected excitatory and inhibitory neurons receives input from a set of input neurons that are tuned to different stimulus orientations (cf. Fig. 1*B*, *Bottom*). Every 200ms a different orientation is presented to the network (vertical gray lines). At the same time, all synapses exhibit plasticity according to a synapse-type-specific Hebbian rule (*Materials and Methods*). *Bottom*: typical firing rate activity of one excitatory (blue) and one inhibitory (red) neuron before and after training. (*B*) Feedforward tuning curves of $N_{E}=10$ excitatory neurons before ($t_{0}$, *Top*), during ($t_{1}$, *Center*), and after ($t_{2}$, *Bottom*) training. Synaptic weights were initialized randomly. Different color shades indicate weights of different postsynaptic neurons. Compare Movies S1 and S2. (*C*) Feedforward population tuning uniformity (*Materials and Methods*) of excitatory and inhibitory neurons in *B*. Time points $t_{0},t_{1},t_{2}$ correspond to time points in *B*. (*D*) Connectivity matrices after training a network of $N_{E}=80$ excitatory (blue) and $N_{I}=20$ inhibitory (red) neurons. Neurons are sorted according to their preferred orientation $\hat{\theta }$, as measured by their peak response to different oriented gratings. $w_{\text{max}}^{\mathrm{AB}}$ is the largest synaptic weight between population A and B; A,B∈{E,I}. (*E*) Normalized (norm.) recurrent weight strengths as a function of the difference between preferred orientations of the pre- and postsynaptic neurons, $\Delta \hat{\theta }={\hat{\theta }}_{\text{post}}-{\hat{\theta }}_{\text{pre}}$, averaged over all neuron pairs. Input weights to excitatory (solid) and inhibitory (dashed) neurons overlap. (*F*) Average firing rate response of inhibitory and excitatory neurons to a stimulus orientation θ, relative to their preferred orientation, $\Delta \theta =\hat{\theta }-\theta$, averaged over all neurons. Curves for excitatory (blue) and inhibitory (red) neurons overlap. (*G*) Same as in *F*, but for average excitatory and inhibitory inputs to excitatory neurons. (*H*) Inhibitory input to an excitatory neuron with preferred orientation close to $90^{{}^{\circ}}$. Each curve corresponds to the input from one presynaptic inhibitory neuron for stimuli of different orientations θ.

Recent experimental studies have suggested that inhibitory neurons drive decorrelation of neural activities (46, 47). Hence, we asked whether the interaction between excitatory and inhibitory neurons can serve to decorrelate not only inhibitory but also excitatory neural activities. To address this question we explored the consequences of synapse-type-specific competitive Hebbian learning in a network of recurrently connected excitatory and inhibitory neurons. We presented different oriented gratings in random order to a network where all feedforward and recurrent synapses are plastic (Fig. 3*A*, *Top*). We observed a sharp increase in response selectivity (Fig. 3*A*, *Bottom*) that is reflected in the reconfiguration of feedforward synaptic weights (cf. Movies S1 and S2): Shortly after random initialization (Fig. 3 *B*, *Top*), excitatory neurons predominantly connect to a subset of input neurons with similar stimulus selectivities (Fig. 3*B*, *Center left*). We quantified the uniformity of the distribution of feedforward tuning curves during training (Fig. 3*C*; *Materials and Methods*) and found that inhibitory neurons maintained a much wider coverage of the input stimulus space than the excitatory population (cf. Fig. 3*B*, *Center*, $t_{1}$). Eventually, tuning curves of excitatory as well as inhibitory neurons decorrelate and cover the whole stimulus space with minimal overlap (Fig. 3*B*, *Bottom*), in sharp contrast to circuits without inhibition, where tuning curves become clustered (cf. Fig. 2*C*). After training, neurons are organized in an assembly-like structure. Neurons that are similarly tuned became more strongly connected (Fig. 3*D* and *E*), as is observed experimentally (48–58). We found that inhibitory neurons become as selective for stimulus orientations as excitatory neurons (34–37) (Fig. 3*F*), while excitatory input is balanced by similarly tuned inhibitory input (Fig. 3*G*) from multiple overlapping inhibitory neurons (Fig. 3*H*), in agreement with experimental results (12, 59–64); but see refs. 65–70.

In summary, we find that synapse-type-specific competitive Hebbian learning in fully plastic recurrent networks is sufficient to decorrelate neural activities and leads to preferential connectivity between similarly tuned neurons, as observed in cortical circuits.

### Inhibitory Neurons Balance Excitatory Attraction and Enable Decorrelation.

**Fig. 4.**
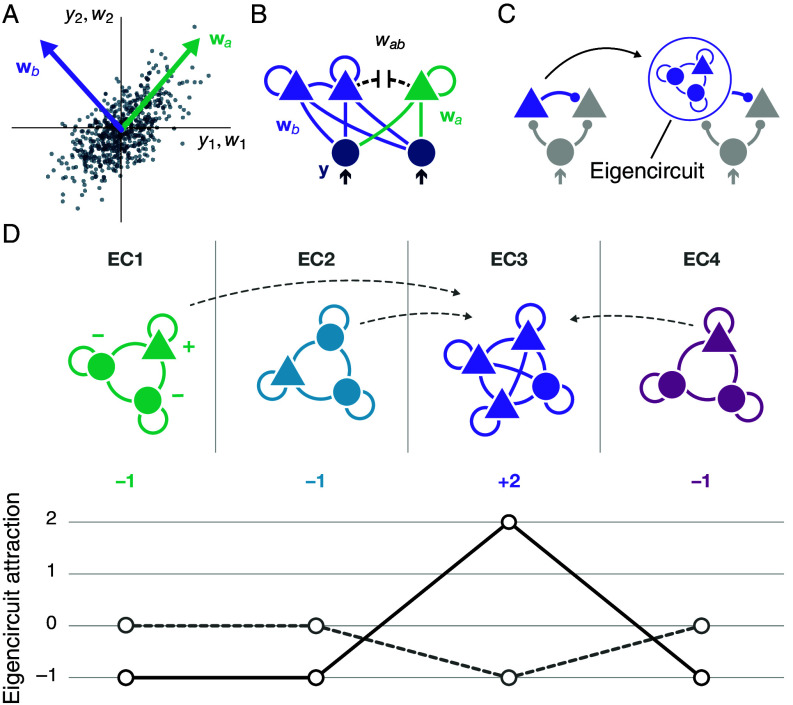
Illustration of eigencircuit decomposition and attraction. (*A*) Feedforward synaptic weight vectors $w_{a}$, $w_{b}$ of two neurons that are tuned to two different principal components (*Top*, purple, and green) of the input data. Each dark blue dot represents one presynaptic firing pattern (cf. Fig. 1*G*). (*B*) Synaptic weights $w_{\mathrm{ab}}$ between neurons that are tuned to different eigenvectors decay to zero, while neurons tuned to the same eigenvector form recurrently connected eigencircuits (purple). (*C*) As single, laterally projecting neurons shape the effective attraction of their input mode (left; cf. Fig. 2), eigencircuits also increase or decrease the effective attraction of their respective eigenvector direction (*Right*). (*D*) A recurrent network of excitatory (triangles) and inhibitory (circles) neurons that are distributed across four decoupled eigencircuits (EC, *Top*). Each excitatory neuron contributes plus one (+), each inhibitory neuron minus one (−) to the eigencircuit attraction, $\lambda_{\text{eig}}$ (solid line, *Bottom*). Due to synaptic plasticity, neurons are pulled toward the most attractive eigencircuit, EC3 (gray dashed arrows, *Top*). After all neurons integrate into the same eigencircuit (EC3), its attraction becomes negative, while the now unoccupied eigencircuits (EC1, EC2, EC4) are neutral (dashed line, *Bottom*).

To uncover how recurrent inhibition can prevent all neurons from becoming selective for a single input mode, we investigated the fundamental principles of synapse-type-specific competitive Hebbian learning in recurrent networks analytically (*SI Appendix*, section 5). In the simplified case of linear activation functions, input modes are eigenvectors of the input covariance matrix (cf. Eq. **3**). Since these eigenvectors are orthogonal by definition (Fig. 4*A*), the activities of neurons that are tuned to different eigenvectors are uncorrelated, and their reciprocal connections decay to zero under Hebbian plasticity (Fig. 4*B*). Then, neurons that are tuned to the same input mode form recurrent “eigencircuits” that are otherwise separated from the rest of the network (*SI Appendix*, section 4). We characterize a mode’s effective attraction as a number such that, if a mode has a higher attraction than a competing mode, then neurons responding to the mode with lower attraction are unstable and shift their tuning toward the mode with higher attraction (see *SI Appendix* for details). Just like single, laterally projecting neurons (*SI Appendix*, section 3), eigencircuits also modify the effective attraction of their input mode (Fig. 4*C*). The decomposition of the network into eigencircuits allows to write the effective attraction $\overset{¯}{\lambda }$ of each input mode as the sum of a feedforward component λ and the variances of the firing rates of the neurons that reside in the respective eigencircuit (cf. *SI Appendix*, sections 4.1 and 4.2):

[7] $$\begin{array}{c} \overset{¯}{\lambda }=\lambda +\lambda_{\text{eig}}=\lambda +\sum_{i}\sigma_{E,i}^{2}-\sum_{j}\sigma_{I,j}^{2}, \end{array}$$

where we defined the contribution of recurrently projecting neurons to the effective attraction of an input mode as the eigencircuit attraction, $\lambda_{\text{eig}}$. Note that, in general, variances $\sigma_{E/I}^{2}$ depend on the total synaptic weights, and the number of excitatory and inhibitory neurons in the eigencircuit (*SI Appendix*, section 4.2). This reveals that the attractive and repulsive effects of excitatory and inhibitory neurons can balance each other. In a simplified example, we assumed that all input modes have equal feedforward attraction, equal to λ, while each excitatory neuron contributes plus one and each inhibitory neuron minus one to the effective attraction (Fig. 4*D*, *Top*). Then the eigencircuit attractions becomes $\lambda_{\text{eig}}=n_{E}-n_{I}$ (Fig. 4*D*, *Bottom*, solid line). In this configuration, the network is unstable: All neurons are attracted toward the input mode with the highest effective attraction (EC3), which suggests that all tuning curves will eventually collapse onto the same input mode. However, when all neurons become selective to the most attractive input mode, that mode would become repulsive (Fig. 4*D*, *Bottom*, dashed gray line), as each increase in attraction due to an additional excitatory neuron is balanced by a decrease in attraction due to two additional inhibitory neurons. Consequently, the resulting eigencircuit is unstable and neurons are repelled toward nonrepulsive, unoccupied input modes; distributed across the stimulus space.

While this example conveys the core principle of how recurrently connected neurons adjust their tunings, the actual dynamics of synaptic weights are more complex (*SI Appendix*, section 5). In particular, neurons do not switch their tuning between input modes in discrete steps but shift their tuning gradually. Due to the recurrent nature of the circuit, even small tuning shifts affect the attractions of the respective eigencircuits (cf. *SI Appendix*, section 5.2.3). In our simulations, we therefore never observe a full collapse of all tuning curves onto the same input mode before neurons distribute across the stimulus space. Instead, neurons rapidly develop tuned feedforward receptive fields that gradually shift to maximize tuning uniformity, with little to no oscillatory dynamics (Fig. 3*B* and *C* and Movies S1 and S2). In the simplified case of linear activation functions, we derive the following condition that prevents the collapse of all tuning curves onto a single input mode:

[8] $$\begin{array}{c} N_{E}\sigma_{E}^{2}<N_{I}\sigma_{I}^{2}, \end{array}$$

where $\sigma_{E}^{2}$, $\sigma_{I}^{2}$ are the average of the variances of the excitatory and inhibitory firing rates, and $N_{E}$, $N_{I}$ are the total number of neurons in the network (cf. *SI Appendix*, section 5.2.4). These results show that recruiting recurrent inhibition can prevent tuning curve collapse and enables decorrelation, where a lower number of inhibitory neurons can be compensated by an increase in neural activation.

### Plastic Recurrent E–I Networks Perform Response Normalization and Exhibit Winner-Takes-All Dynamics.

**Fig. 5.**
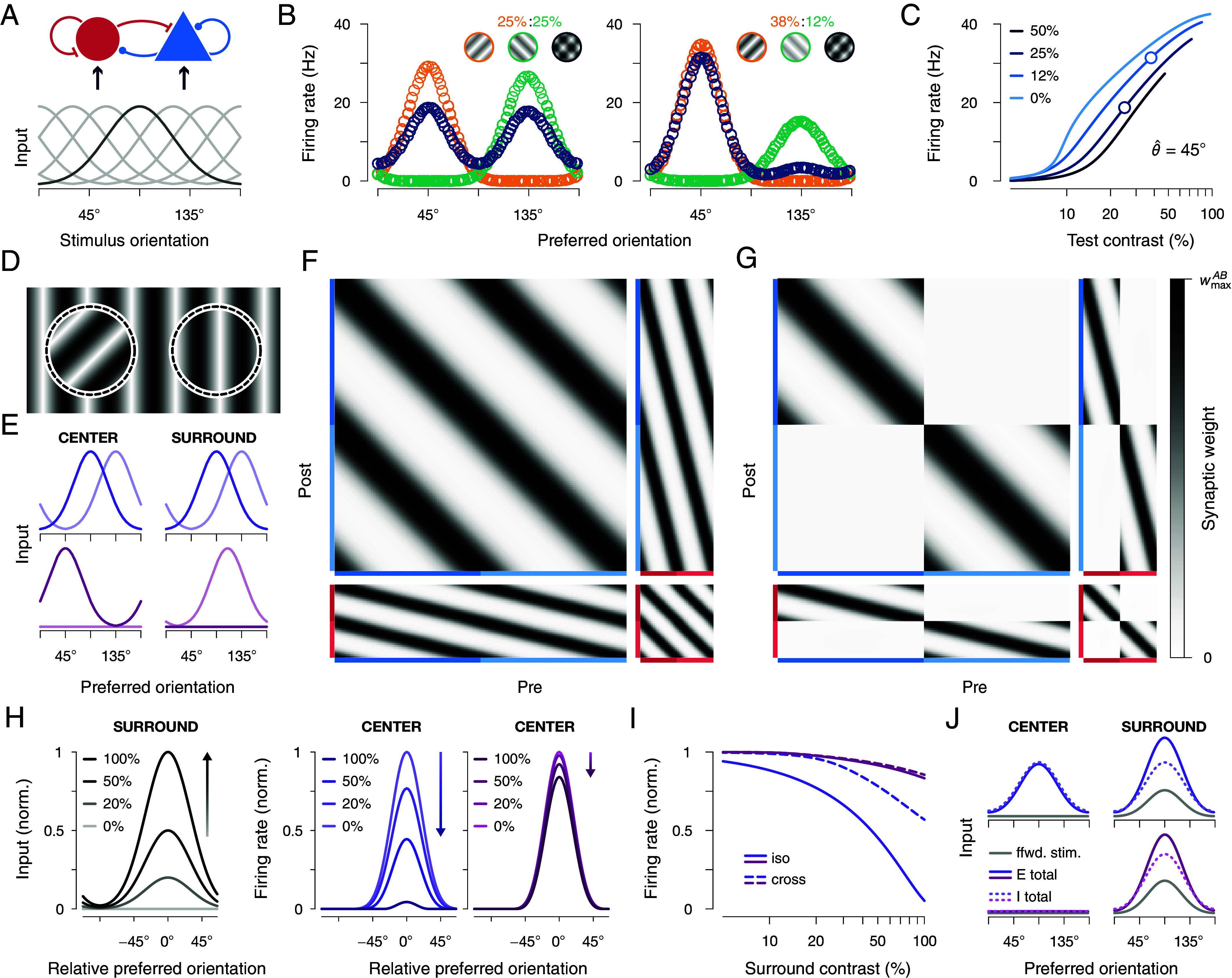
Cross-orientation and surround suppression in trained neural networks. (*A*) A plastic recurrent network of excitatory and inhibitory neurons (*Top*) receives input according to fixed feedforward tuning curves (*Bottom*). Input amplitudes were modulated with stimulus contrast. Tuning curve of neurons with preferred orientation of $90^{{}^{\circ}}$ highlighted in dark gray. (*B*) Response of 80 excitatory neurons to a test grating (orange, $45^{{}^{\circ}}$) and a mask grating (green, $135^{{}^{\circ}}$) of different contrast levels (*Insets*, grating contrasts increased for better visibility). Gratings are presented separately (orange & green) or together (dark blue). Each open circle corresponds to the response of one excitatory neuron. (*C*) Contrast response curve of a single excitatory neuron with preferred orientation $\hat{\theta }=45^{{}^{\circ}}$ to the test and mask gratings in *B*. Different mask contrasts are indicated by different color shades. The bottom/top circles correspond to the left/right contrast level configurations in *B*. (*D*) Center (*Left*) and surround region (*Right*) with different oriented stimuli. (*E*) Example stimuli during training with different stimulus statistics. *Top*: Neurons tuned to the same orientation, but different regions (center region, *Left*; or surround region, *Right*) receive identical input; two example stimuli are shown in solid and transparent purple, respectively. *Bottom*: Neurons tuned to the center, and surround regions are stimulated separately; two example stimuli are shown in solid and transparent red, respectively. Either the surround or the center regions are stimulated, while the other region receives zero input. (*F*) Recurrent connectivity matrix between excitatory (blue) and inhibitory (red) neurons (cf. Fig. 3*D*) after training the network with correlated center and surround stimuli (corresponds to purple color in *E*, *Top*). Neurons are sorted according to their feedforward orientation tuning. Color shades indicate tuning to the center (dark) or surround (light) region. (*G*) Same as in *F*, but for a network trained with single gratings that were presented either in the center or the surround region (corresponds to red color in *E*, *Bottom*). (*H*) Suppression of excitatory population activity in response to increasing surround stimulation for two networks trained under different stimulus statistics. *Left*: network stimulation. Neurons tuned to the center region are stimulated by an oriented grating of constant, 100% contrast (not shown) while neurons tuned to the surround region are stimulated with an oriented grating of increasing contrast (shades; compare *Insets*). Identical stimulation protocol for both training statistics. *Center* and *Right*: network response. The activity of excitatory neurons that are tuned to the center region is suppressed with increasing surround contrast. The magnitude of suppression depends on the stimulus statistics during training (purple vs. red, colors as in *E*). (*I*) Response of one excitatory neuron to center and surround stimulation after training. A center stimulus of preferred orientation was presented at constant contrast while the contrast of a cross- (dashed) or iso-oriented (solid) surround stimulus changed. Colors indicate different stimulus statistics during training (as in *E*). (*J*) Total excitatory (solid) and inhibitory (dotted) input to excitatory neurons during stimulation of only the surround region with an oriented grating of $90^{{}^{\circ}}$. Excitatory input due to feedforward stimulation (ffwd. stim.) is shown in light gray. Colors (*Top* vs. *Bottom*) indicate different input statistics during training (as in *E*).

Our results thus far reveal how synapse-type-specific competitive Hebbian learning can explain the development of structured recurrent connectivity. We next asked whether synapse-type-specific competitive Hebbian learning can also explain the emergence of nonlinear network computations. For example, the firing rate response of neurons in the visual cortex to multiple overlayed oriented gratings is normalized in a nonlinear fashion (71, 72). While this form of normalization is mostly of thalamic origin (73–75), there is most likely also a cortical component (72, 76). A recently introduced E–I network model with static, hand-crafted connectivity can explain these modulations (18, 77). We explored whether the recurrent connectivity can instead be learned from a network’s input stimulus statistics. We consider a circuit with fixed feedforward tuning and plastic recurrent connectivity (Fig. 5*A*). After training the network with single oriented grating stimuli (Fig. 5*A*, *Bottom*), we found that neural responses to a cross-oriented mask grating that is presented in addition to a regular test grating are normalized, i.e., the response to the combined stimulus is weaker than the sum of the responses to the individual gratings (Fig. 5*B*, *Left*). When the contrast of the mask grating is lower than the test grating’s, the network responds in a winner-takes-all fashion: The higher-contrast test grating dominates activities while the lower-contrast mask grating is suppressed (Fig. 5*B*, *Right*). As observed experimentally (71, 72, 78), we found that this orientation-specific response normalization is divisive and shifts the log-scale contrast-response function to the right (Fig. 5*C*).

### Sensory Input Statistics Shape Computational Functions of Recurrent Circuits.

We next investigated how the stimulus statistics during training affect receptive field properties. We considered a plastic network where two neural populations receive tuned input from either a center or a surround region of the visual field (Fig. 5*D*). During training, we presented either the same oriented grating in both regions (Fig. 5*E*, *Top*, purple), or a single grating in just one region (Fig. 5*E*, *Bottom*, red), at 50% contrast (cf. Table 1). These stimulus statistics heavily influenced the recurrent connectivity structure in the network. When identical oriented stimuli are presented to the center and surround regions during training, neurons with similar orientation tuning become most strongly connected, independent from which region the neurons receive their feedforward input (Fig. 5*F*). However, when the center and surround regions are stimulated separately during training, neurons only connect to similarly tuned neurons within the same region and cross-region connectivity decays to zero (Fig. 5*G*). These differences in the recurrent connectivity structure are also reflected in the networks’ response properties. We found that after training, the response of center-tuned neurons exhibits orientation-specific surround suppression, reflecting the stimulus statistics during training. When the center and the surround regions are stimulated separately during training, iso- or cross-oriented stimuli in the surround both elicit minimal suppression of the center-tuned population’s response to a center stimulus (Fig. 5*H* and *I*, red). In contrast, in the case of correlated stimulation of the center and surround regions during training, the response of the center population is markedly suppressed when an additional surround stimulus is presented (Fig. 5*H* and *I*, purple). Importantly, suppression is stronger for iso- compared to cross-orientations (Fig. 5*I*, solid and dashed lines), as has been reported experimentally (79–82). We further investigated the lateral interactions between neurons tuned to the center and surround regions by presenting an oriented stimulus only in the surround region, while observing the total excitatory and inhibitory inputs to excitatory neurons (Fig. 5*J*). We found that the total excitatory input to stimulated excitatory neurons in the surround was larger than the total inhibitory input (Fig. 5*J*, right column). When center and surround neurons were stimulated together during training, both center and surround received similar, balanced E and I recurrent input, but the surround cells also received feedforward excitation, yielding more total excitation (Fig. 5*J*, *Top*, purple). When center neurons were not stimulated with the surround neurons during training, they received no input from a surround-only stimulus (Fig. 5*J*, *Bottom*, red). In the case of correlated stimulation of the center and surround regions during training, this lateral input was orientation-specific. Center neurons tuned to the same orientations as stimulated neurons in the surround received stronger input than center neurons tuned to different orientations (Fig. 5*J*, *Top left*), reflecting the input stimulus statistics during training (Fig. 5*E*) and the resulting recurrent connectivity (Fig. 5*F*). A similar balance of excitatory and inhibitory lateral inputs has previously been observed in the barrel cortex (83). Together, this demonstrates that synapse-type-specific competitive Hebbian learning produces extraclassical receptive fields that modulate feedforward responses via recurrent interactions that reflect the input statistics during training.

**Table 1.** Simulation parameters Different parameters for different panels are separated by commas

Figure	1*E* & *F*, *H* & *I*	2*A*–D (*A*, *B*)	3*A*–*C*, *D*–*H*	5*A*–*C*, *D*–*J*
$N_{E}$	1	2, 1, 2, 0	10, 80	80, 80×2
$N_{I}$	10	0, 5, 0, 5	10, 20	20, 20×2
$N_{F}$	10	50	40, 80	80, 80×2
a	1	0.04 (0.2, 0.08)	0.04	0.04
b	0.25	0	0	0
n	2	2	2	2
$\mu_{W}$	0.1	0.1	0.2	0.2
$\sigma_{W}$	0.05	0.01	0.1	0.1
$W_{\mathrm{EE}}$	10	1, 1, 4, -	2, 0.6	3.51
$W_{\mathrm{IE}}$	-	-	2, 0.85	3.35
$W_{\mathrm{EI}}$	5, -	-, 0.5, -, -	0.8, 0.3	1.84
$W_{\mathrm{II}}$	-	-, -, -, 0.5	0.5, 0.35	1.44
$W_{\mathrm{EF}}$	-	(0.9, -)	-	$1.4^{‡}$
$W_{\mathrm{IF}}$	-	(-, 1)	-	$1.4^{‡}$
c	1	1	1	0.5
$A_{F}$	1	1	35, 140	80
$\sigma_{F}$	$20^{{}^{\circ}}$	$12^{{}^{\circ}}$	$12^{{}^{\circ}}$	$30^{{}^{\circ}}/\sqrt{2}$
$\sigma_{\theta }$	-	-, -, $22^{{}^{\circ}}$, $22^{{}^{\circ}}$ ($22^{{}^{\circ}}$, $16^{{}^{\circ}}$)	-	$30^{{}^{\circ}}/\sqrt{2}$
Δt	200ms	20ms	10ms	10ms
$\tau_{E}$	200ms	40ms	20ms	25ms
$\tau_{I}$	-	28ms	17ms	12.5ms
$\epsilon_{\mathrm{EE}}$	-	$0.4\times 10^{-8}\;{\mathrm{ms}}^{-1}$	$2\times 10^{-9}\;{\mathrm{ms}}^{-1}$, $1.0\times 10^{-10}\;{\mathrm{ms}}^{-1}$	$1.0\times 10^{-9}\;{\mathrm{ms}}^{-1}$
$\epsilon_{\mathrm{IE}}$	-	$0.8\times 10^{-8}\;{\mathrm{ms}}^{-1}$	$3\times 10^{-9}\;{\mathrm{ms}}^{-1}$, $1.5\times 10^{-10}\;{\mathrm{ms}}^{-1}$	$1.5\times 10^{-9}\;{\mathrm{ms}}^{-1}$
$\epsilon_{\mathrm{EI}}$	$2\times 10^{-4}\;{\mathrm{ms}}^{-1}$, $4\times 10^{-4}\;{\mathrm{ms}}^{-1}$	$0.6\times 10^{-8}\;{\mathrm{ms}}^{-1}$	$4\times 10^{-9}\;{\mathrm{ms}}^{-1}$, $2.0\times 10^{-10}\;{\mathrm{ms}}^{-1}$	$2.0\times 10^{-9}\;{\mathrm{ms}}^{-1}$
$\epsilon_{\mathrm{II}}$	-	$1.0\times 10^{-8}\;{\mathrm{ms}}^{-1}$	$5\times 10^{-9}\;{\mathrm{ms}}^{-1}$, $2.5\times 10^{-10}\;{\mathrm{ms}}^{-1}$	$2.5\times 10^{-9}\;{\mathrm{ms}}^{-1}$
$\epsilon_{\mathrm{EF}}$	$1\times 10^{-4}\;{\mathrm{ms}}^{-1}$, $2\times 10^{-4}\;{\mathrm{ms}}^{-1}$	$\epsilon_{\mathrm{EE}}$	$\epsilon_{\mathrm{EE}}$	-
$\epsilon_{\mathrm{IF}}$	-	$\epsilon_{\mathrm{IE}}$	$\epsilon_{\mathrm{IE}}$	-
$r_{0}$	-, 0.25	-	-	-

Dashes indicate that parameters were not used in the simulation. For Fig. 5, weight norms of static synaptic weights are indicated by “‡.” For Fig. 2, parameters of static, laterally projecting neurons are given in brackets (comma separated for panels *A* and *B*).

## Discussion

Our results suggest that synapse-type-specific competitive Hebbian learning is the key mechanism that enables the formation of functional recurrent networks. Rather than hand-tuning connectivity to selectively explain experimental data, our circuits emerge from a single unsupervised, biologically plausible learning paradigm that acts simultaneously at all synapses. In a single framework, our networks readily explain multiple experimental observations, including the development of stimulus selectivity, excitation–inhibition balance, decorrelated neural activity, assembly structures, response normalization, and orientation-specific surround suppression. These results demonstrate how the connectivity of inhibition-balanced networks is shaped by their input statistics and explain the experience-dependent formation of extraclassical receptive fields (84–88). Unlike previous models (89–94), our networks are composed of excitatory and inhibitory neurons with fully plastic recurrent connectivity.

Early theoretical work on inhibitory plasticity assumed that synapses evolve to maintain the mean firing rate of postsynaptic excitatory neurons (23). When excitatory input is static, this leads to neural tunings where inhibition and excitation are balanced. However, when excitatory synapses are simultaneously plastic according to a simple Hebbian rule, the circuit is unstable and can not explain the joint development of feedforward stimulus tuning and inhibitory balance (24) (*SI Appendix*, section 1.2.3). The system can be stabilized when the Hebbian growth of excitatory synapses is controlled by a BCM-like plasticity threshold. This introduces fierce competition between different input streams in the form of subtractive weight normalization, which leads to winner-takes-all dynamics among synapses that do not allow for the development of extended receptive fields (24, 31, 95). Later models have proposed more intricate plasticity rules, some of which consider, e.g., voltages or currents, in addition to pre- and postsynaptic action potentials (25, 28, 96–102), as summarized in several recent reviews (14, 103–106). In recent years, there has also been a resurgence of interest in normative approaches (28, 29, 107). In these approaches, it is postulated that synaptic plasticity rules act to optimize an objective function that describes a desirable network property. Motivated by the notorious instability of recurrent networks, one obvious objective is stability, e.g., in the form of firing rate homeostasis.

Following early theoretical work that suggested such a homeostatic role for synaptic plasticity of inhibitory synapses onto excitatory neurons (23), two recent studies propose a similar role for the plasticity of other recurrent synapse types (28, 29). Indeed, such plasticity rules allow the formation of inhibition-balanced receptive fields (28), and stabilize network activity, even when faced with strong recurrent connections (29). However, none of these rules have been applied in fully plastic recurrent networks with structured feedforward input. Even in complex models that use many different forms of plasticity, some synapse types are kept static after initialization, to maintain stable network activity (23, 26, 27, 102). While such networks still show many interesting dynamics, they lack the rich computational functions of circuits with structured connectivity between all neuron types (18, 77). In contrast, our learning rule is minimalistic and only relies on general Hebbian synaptic growth that is stabilized by competitive interactions. Importantly, our theory does not depend on a specific biophysical implementation of the Hebbian plasticity paradigm. We only require that synapses follow the basic Hebbian principle of synaptic strengthening following concurrent pre- and postsynaptic activity. In the past, competitive Hebbian learning has been investigated theoretically for excitatory synaptic inputs to single neurons (30, 31, 39, 108, 109), but not for inhibitory inputs or in recurrent networks. Our analysis demonstrates that competitive Hebbian plasticity is a suitable learning mechanism for networks of recurrently connected excitatory and inhibitory neurons, while being analytically tractable and biologically plausible.

Competitive interactions between synapses have been observed in many different preparations and have been attributed to various mechanisms (110–121). While previous work has focused on competitive interactions between excitatory synapses, our results support the notion that similar competitive processes are also active at inhibitory synapses (32, 122). The local competition for a limited supply of synaptic building blocks is a biologically plausible normalization mechanism (33, 115, 120, 123, 124). Many synaptic proteins are specific to inhibitory or excitatory synapses and reside in one synapse-type, but not the other (125, 126). Therefore, in this work, we assume a synapse-type-specific competition for different synaptic resource pools and implement separate normalization constants for inhibitory and excitatory synapses. On a finer scale, synapses of different excitatory and inhibitory neuron subtypes also differ in their protein composition (126–129). In principle, this allows for the precise regulation of different input pathways via the adjustment of subtype-specific resource pools (130–136). Furthermore, axons of different neuron subtypes target spatially separated regions on the dendritic tree, allowing for pathway-specific local competition. For example, somatostatin-positive cortical Martinotti cells target the apical dendritic tree of pyramidal cells, while parvalbumin-positive basket cells form synapses closer to the soma (1), which suggests that afferents of these cell types compete for separate resources pools. We anticipate such subtype-specific mechanisms to be crucial for the functional development of any network with multiple neuron subtypes (137, 138).

In the brain, total synaptic strengths are dynamic and homeostatically regulated on a timescale of hours to days (139–142). In addition to maintaining average firing rates in response to network-scale perturbations, a prominent framework puts forward homeostatic scaling of synaptic strengths as a stabilizing mechanism of Hebbian growth (143). However, theoretical models suggest that homeostatic scaling is too slow to balance rapid synaptic plasticity (144). In our networks, Hebbian growth is instead thought to be stabilized by the competition for a limited pool of synapse-type-specific resources, while total synaptic strengths remain fixed. This competition is fast due to rapid interactions on a molecular level (33, 120). Compared to Hebbian growth, infinitely fast, as a synapse can only grow at the expense of another. Therefore, we suggest that homeostatic scaling of total synaptic strengths is not required for immediate network stability but instead controls the operating regime of the network (16, 77, 145).

Our results demonstrate how multisynaptic, inhibitory interactions can decorrelate excitatory neurons. In contrast, inhibitory neurons can inhibit each other monosynaptically and do not require additional recurrent interactions for decorrelation. Accordingly, we observe that during training, inhibitory neurons are more decorrelated compared to excitatory neurons (Fig. 3*C*). These insights complement recent experimental results that suggest an instrumental role of inhibition in the decorrelation of excitatory networks in the mouse prefrontal cortex during early development (47). Recent experimental studies in the ferret visual cortex report conflicting evidence—either supporting (46) or contradicting (146) aligned developmental trajectories of excitatory and inhibitory populations. In our simulations, we observe similar developmental trajectories for excitatory and inhibitory populations. However, we focused on synaptic plasticity and did not consider other processes, like critical periods (147, 148), that are known to shape circuit development.

Cortical computations rely on strong recurrent synaptic weights that result in neural activities that can deviate significantly from the input stimulus pattern (15, 16, 18) (cf. Fig. 5*B*, *Left*, combined grating response). Such a decoupling of network activity from feedforward input due to recurrent interactions can lead to neural tunings that do not reflect the input stimulus statistics (cf. *SI Appendix*, section 3).In our theory (*SI Appendix*, section 4), we assume that neurons are tuned to feedforward modes and thereby implicitly assume that network activity is dominated by feedforward input. In our numerical simulations of fully plastic recurrent networks, we find that for intermediate levels of recurrence (cf. Table 1 and Figs. 1–3), the network’s activities are indeed dominated by feedforward inputs. In case of strong recurrence (Fig. 5), we ensure feedforward dominance by presenting single oriented gratings that match the fixed feedforward tunings of neurons (cf. Fig. 5). Such gratings elicit a Gaussian-shaped response that is sharpened due to the recurrent connectivity, but maintains the general correlation structure compared to purely feedforward-driven networks (compare tuning widths in Fig. 5*A*, *Bottom*, and *B*, single grating response). Biological cortical networks are strongly recurrently connected (149–153). However, neural activity and the induction and polarity of synaptic plasticity are regulated by neuromodulators (154–158), which may control the destabilizing effect of strong recurrent connectivity. In addition, different synapse types do not develop simultaneously but progress through different developmental stages (137, 159, 160). For example, the development of recurrent excitatory connections is delayed compared to that of feedforward synapses (131, 161). Taking these factors into account will be essential for future models of developing recurrent circuits.

In our networks, structured feedforward input is crucial for the development of orientation-selective receptive fields. However, already at the time before eye opening cortical neurons exhibit substantial selectivity for stimulus orientation, without having been exposed to the statistical regularities of visual inputs (162–164). One hypothesis is that, instead, spontaneous activity in the retina provides the statistical structure required for the initial development of orientation selectivity (165–167). In our model, circuit formation depends only on the statistical regularities between input streams and is agnostic with respect to their origin. Therefore, we expect our approach to extend beyond sensory cortices and to provide a fundamental framework for plasticity in recurrent neural networks.

## Materials and Methods

### Computational Model.

We consider networks of rate coding excitatory (E) and inhibitory (I) neurons that receive input from themselves and a population of feedforward input neurons (F). Membrane potential vectors u evolve according to

[9] $$\begin{array}{c} \tau_{A}{\dot{u}}_{A}=-u_{A}+W_{\mathrm{AF}}r_{F}+W_{\mathrm{AE}}r_{E}-W_{\mathrm{AI}}r_{I},\;A\in \left\{E,I\right\}, \end{array}$$

where $\tau_{A}$ is the activity timescale. **W**_*AB*_ are matrices that hold synaptic weights between the presynaptic population B and the postsynaptic population A with B∈{E,I,F}. All differential equations were numerically integrated using the Euler method in timesteps of Δt. Entries of weight matrices were drawn from a normal distribution with mean $\mu_{W}$ equal to two times the SD $\sigma_{W}$, which yields mainly positive entries. Negative entries were set to their absolute value. Before the start of the simulation, excitatory and inhibitory weights were normalized as described below. Unless stated otherwise, prior to normalization, all *recurrent* excitatory weights were set to zero, i.e., initially networks were dominated by feedforward input. Firing rate vectors $r_{A}$ are given as a function $f(u_{A})$ of the membrane potential $u_{A}$:

[10] $$\begin{array}{c} r_{A}=f\left(u_{A}\right),\;f\left(u_{A}\right)=a{\left[u_{A}-b\right]}_{+}^{n},\;A\in \left\{E,I\right\} \end{array}$$

with ${\left[\cdot \right]}_{+}=\text{max}\left(0,\cdot \right)$ and scalar constants a, b, and n.

### Plasticity and Normalization.

Plastic weights evolve according to a Hebbian plasticity rule

[11] $$\begin{array}{c} {\dot{W}}_{\mathrm{AB}}=\epsilon_{\mathrm{AB}}r_{A}r_{B}^{⊺},\;A\in \left\{E,I\right\},\;B\in \left\{E,I,F\right\} \end{array}$$

where $\epsilon_{\mathrm{AB}}$ is a scalar learning rate, and ^⊺^ indicates the transpose. After each plasticity step, synaptic weights are normalized such that the total excitatory and inhibitory postsynaptic weights are maintained:

[12] $$\begin{array}{cc} w_{\mathrm{AB}}^{\left(ij\right)} & \leftarrow W_{\mathrm{AE}}\frac{w_{\mathrm{AB}}^{\left(ij\right)}}{\sum_{j}w_{\mathrm{AE}}^{\left(ij\right)}+\sum_{k}w_{\mathrm{AF}}^{\left(ik\right)}}, \end{array}$$

[13] $$\begin{array}{cc} w_{\mathrm{AI}}^{\left(ij\right)} & \leftarrow W_{\mathrm{AI}}\frac{w_{\mathrm{AI}}^{\left(ij\right)}}{\sum_{j}w_{\mathrm{AI}}^{\left(ij\right)}},\;A\in \left\{E,I\right\},\;B\in \left\{E,F\right\}, \end{array}$$

where $W_{\mathrm{AE}},W_{\mathrm{AI}}$ are the total excitatory and inhibitory synaptic weight norms. Weights are updated and normalized in every integration timestep Δt, in sync with the network dynamics.

In Fig. 1, we set the activity of the inhibitory input neurons equal to the activity of the excitatory input neurons, i.e., $r_{I}=r_{F}$. For panels *H* and *I* of Fig. 1, inhibitory weights evolved according to the classic inhibitory plasticity rule (23) without normalization:

[14] $$\begin{array}{c} {\dot{w}}_{\mathrm{EI}}=\epsilon_{\mathrm{EI}}\left(r_{E}-r_{0}\right)r_{I}, \end{array}$$

where $r_{0}$ is a target firing rate.

### Input Model.

The activity of feedforward input neurons depends on the orientation θ and contrast c of an input grating:

[15] $$\begin{array}{c} r_{F}=cA_{F}\text{exp}\left(-\frac{|\theta ,\theta_{F}{|}^{2}}{2\sigma_{F}^{2}}\right), \end{array}$$

where the vector $\theta_{F}$ holds the preferred orientations of the input neurons that are evenly distributed between 0 and $180^{{}^{\circ}}$, $\sigma_{F}$ is the tuning width, $A_{F}$ the maximum firing rate, and |·,·| is the angular distance, i.e., the shortest distance around a circle of circumference $180^{{}^{\circ}}$. During training, single gratings, sampled from a uniform distribution between $0^{{}^{\circ}}$ and $180^{{}^{\circ}}$, were presented to the network for 200ms, before the next stimulus was selected.

In Fig. 5 network stimulation is realized via static feedforward weights. Neurons were assigned a preferred orientation $\hat{\theta }$, evenly distributed between $0^{{}^{\circ}}$ and $180^{{}^{\circ}}$. Static feedforward weights were initialized as

[16] $$\begin{array}{c} W_{\mathrm{AF}}=\text{exp}\left(-\frac{|\hat{\theta },\theta_{F}{|}^{2}}{2\sigma_{\theta }^{2}}\right). \end{array}$$

For Fig. 5, feedforward weights are normalized separately to $W_{\mathrm{AF}}$ before the start of the simulations (cf. Table 1). In this case, feedforward weights are fixed and are not taken into account when normalizing recurrent weights. Feedforward weights of static neurons in Fig. 2*A* and *B* are processed in the same fashion. For Fig. 5, parameters were selected to result in stimulation patterns as in Rubin et al. (18). Weight norms $W_{\mathrm{AB}}$ were also adapted from Rubin et al. (18). See Table 1 for an overview of used simulation parameters.

### Tuning Curve Uniformity Measure.

In Fig. 3*C*, we quantified the uniformity of the distribution of tuning curves during learning and defined:

[17] $$\begin{array}{c} p_{j}^{A}=\sum_{i}w_{\mathrm{AF}}^{\left(ij\right)}/\sum_{\mathrm{ij}}w_{\mathrm{AF}}^{\left(ij\right)},\;A\in \left\{E,I\right\}, \end{array}$$

where $p_{j}^{A}$ is the normalized total synaptic output weight of input neuron j onto the excitatory (E) and inhibitory (I) neural population. Then $\sum_{j}p_{j}^{A}=1$, and we can define the tuning uniformity $U_{A}$ as the normalized Shannon entropy $H_{p^{A}}$.

[18] $$\begin{array}{c} U_{A}=H_{p^{A}}/\log\left(N_{F}\right)=-\sum_{j}p_{j}^{A}log\left(p_{j}^{A}\right)/\log\left(N_{F}\right). \end{array}$$

$U_{A}$ is maximal, equal to one, if $p^{A}$ is uniformly distributed, and minimal, equal to zero, if all synaptic weight is concentrated in a single input neuron. Such a concentration is highly unlikely. In our simulations, weight distributions are much closer to a uniform distribution, and the uniformity measure is close to one.

## Supplementary Material

Appendix 01 (PDF)

Movie S1. **Decorrelation of feedforward tuning curves of excitatory neurons in plastic recurrent networks**. Development of feedforward tuning curves of *N_E_* = 10 excitatory neurons (cf. Figs. 3*A* & *B*). Synaptic weights were initialized randomly. Different color shades indicate weights of different post-synaptic neurons.

Movie S2. **Decorrelation of feedforward tuning curves of inhibitory neurons in plastic recurrent networks**. Development of feedforward tuning curves of *N_I_* = 10 inhibitory neurons (cf. Figs. 3*A* & *B*). Synaptic weights were initialized randomly. Different color shades indicate weights of different post-synaptic neurons.

## Data Availability

All study data are included in the article and/or supporting information. Python code to reproduce the key results of the study is publicly available on GitHub (168).

## References

[1] R. Tremblay, S. Lee, B. Rudy, Gabaergic interneurons in the neocortex: From cellular properties to circuits. Neuron **91**, 260–292 (2016). 27477017 10.1016/j.neuron.2016.06.033 PMC4980915

[2] R. Hattori, K. V. Kuchibhotla, R. C. Froemke, T. Komiyama, Functions and dysfunctions of neocortical inhibitory neuron subtypes. Nat. Neurosci. **20**, 1199–1208 (2017). 28849791 10.1038/nn.4619 PMC7082034

[3] K. A. Pelkey , Hippocampal gabaergic inhibitory interneurons. Physiol. Rev. **97**, 1619–1747 (2017). 28954853 10.1152/physrev.00007.2017 PMC6151493

[4] A. Kepecs, G. Fishell, Interneuron cell types are fit to function. Nature **505**, 318–326 (2014). 24429630 10.1038/nature12983 PMC4349583

[5] M. Carandini, D. J. Heeger, Normalization as a canonical neural computation. Nat. Rev. Neurosci. **13**, 51 (2012). 10.1038/nrn3136 PMC3273486 22108672

[6] A. Angelucci , Circuits and mechanisms for surround modulation in visual cortex. Annu. Rev. Neurosci. **40**, 425–451 (2017). 28471714 10.1146/annurev-neuro-072116-031418 PMC5697758

[7] K. C. Wood, J. M. Blackwell, M. N. Geffen, Cortical inhibitory interneurons control sensory processing. Curr. Opin. Neurobiol. **46**, 200–207 (2017). 28938181 10.1016/j.conb.2017.08.018 PMC5693245

[8] G. B. Keller, T. D. Mrsic-Flogel, Predictive processing: A canonical cortical computation. Neuron **100**, 424–435 (2018). 30359606 10.1016/j.neuron.2018.10.003 PMC6400266

[9] O. K. Swanson, A. Maffei, From hiring to firing: Activation of inhibitory neurons and their recruitment in behavior. Front. Mol. Neurosci. **12**, 168 (2019). 31333413 10.3389/fnmol.2019.00168 PMC6617984

[10] K. A. Ferguson, J. A. Cardin, Mechanisms underlying gain modulation in the cortex. Nat. Rev. Neurosci. **21**, 80–92 (2020). 31911627 10.1038/s41583-019-0253-y PMC7408409

[11] C. Van Vreeswijk, H. Sompolinsky, Chaos in neuronal networks with balanced excitatory and inhibitory activity. Science **274**, 1724–1726 (1996). 8939866 10.1126/science.274.5293.1724

[12] J. S. Isaacson, M. Scanziani, How inhibition shapes cortical activity. Neuron **72**, 231–243 (2011). 22017986 10.1016/j.neuron.2011.09.027 PMC3236361

[13] S. Denève, C. K. Machens, Efficient codes and balanced networks. Nat. Neurosci. **19**, 375–382 (2016). 26906504 10.1038/nn.4243

[14] G. Hennequin, E. J. Agnes, T. P. Vogels, Inhibitory plasticity: Balance, control, and codependence. Annu. Rev. Neurosci. **40**, 557–579 (2017). 28598717 10.1146/annurev-neuro-072116-031005

[15] S. Sadeh, C. Clopath, Inhibitory stabilization and cortical computation. Nat. Rev. Neurosci. **22**, 21–37 (2021). 33177630 10.1038/s41583-020-00390-z

[16] Y. Ahmadian, K. D. Miller, What is the dynamical regime of cerebral cortex? Neuron **109**, 3373–3391 (2021). 34464597 10.1016/j.neuron.2021.07.031 PMC9129095

[17] H. Ozeki, I. M. Finn, E. S. Schaffer, K. D. Miller, D. Ferster, Inhibitory stabilization of the cortical network underlies visual surround suppression. Neuron **62**, 578–592 (2009). 19477158 10.1016/j.neuron.2009.03.028 PMC2691725

[18] D. B. Rubin, S. D. Van Hooser, K. D. Miller, The stabilized supralinear network: A unifying circuit motif underlying multi-input integration in sensory cortex. Neuron **85**, 402–417 (2015). 25611511 10.1016/j.neuron.2014.12.026 PMC4344127

[19] G. Hennequin, Y. Ahmadian, D. B. Rubin, M. Lengyel, K. D. Miller, The dynamical regime of sensory cortex: Stable dynamics around a single stimulus-tuned attractor account for patterns of noise variability. Neuron **98**, 846–860 (2018). 29772203 10.1016/j.neuron.2018.04.017 PMC5971207

[20] R. Echeveste, L. Aitchison, G. Hennequin, M. Lengyel, Cortical-like dynamics in recurrent circuits optimized for sampling-based probabilistic inference. Nat. Neurosci. **23**, 1138–1149 (2020). 32778794 10.1038/s41593-020-0671-1 PMC7610392

[21] W. Soo, M. Lengyel, Training stochastic stabilized supralinear networks by dynamics-neutral growth. Adv. Neural Inf. Process. Syst. **35**, 29278–29291 (2022).

[22] Y. Luz, M. Shamir, Balancing feed-forward excitation and inhibition via Hebbian inhibitory synaptic plasticity. PLoS Comput. Biol. **8**, e1002334 (2012). 22291583 10.1371/journal.pcbi.1002334 PMC3266879

[23] T. P. Vogels, H. Sprekeler, F. Zenke, C. Clopath, W. Gerstner, Inhibitory plasticity balances excitation and inhibition in sensory pathways and memory networks. Science **334**, 1569–1573 (2011). 22075724 10.1126/science.1211095

[24] C. Clopath, T. P. Vogels, R. C. Froemke, H. Sprekeler, Receptive field formation by interacting excitatory and inhibitory synaptic plasticity. bioRxiv [Preprint] (2016). 10.1101/066589 (Accessed 15 May 2024).

[25] P. D. King, J. Zylberberg, M. R. DeWeese, Inhibitory interneurons decorrelate excitatory cells to drive sparse code formation in a spiking model of v1. J. Neurosci. **33**, 5475–5485 (2013). 23536063 10.1523/JNEUROSCI.4188-12.2013 PMC6705060

[26] A. Litwin-Kumar, B. Doiron, Formation and maintenance of neuronal assemblies through synaptic plasticity. Nat. Commun. **5**, 1–12 (2014). 10.1038/ncomms6319 25395015

[27] F. Zenke, E. J. Agnes, W. Gerstner, Diverse synaptic plasticity mechanisms orchestrated to form and retrieve memories in spiking neural networks. Nat. Commun. **6**, 1–13 (2015). 10.1038/ncomms7922 PMC4411307 25897632

[28] O. Mackwood, L. B. Naumann, H. Sprekeler, Learning excitatory-inhibitory neuronal assemblies in recurrent networks. eLife **10**, e59715 (2021). 33900199 10.7554/eLife.59715 PMC8075581

[29] S. Soldado-Magraner, M. J. Seay, R. Laje, D. V. Buonomano, Paradoxical self-sustained dynamics emerge from orchestrated excitatory and inhibitory homeostatic plasticity rules. Proc. Natl. Acad. Sci. U.S.A. **119**, e2200621119 (2022). 36251988 10.1073/pnas.2200621119 PMC9618084

[30] E. Oja, Simplified neuron model as a principal component analyzer. J. Math. Biol. **15**, 267–273 (1982). 7153672 10.1007/BF00275687

[31] K. D. Miller, D. J. MacKay, The role of constraints in Hebbian learning. Neural Comput. **6**, 100–126 (1994).

[32] J. N. Bourne, K. M. Harris, Coordination of size and number of excitatory and inhibitory synapses results in a balanced structural plasticity along mature hippocampal CA1 dendrites during LTP. Hippocampus **21**, 354–373 (2011). 20101601 10.1002/hipo.20768 PMC2891364

[33] J. Triesch, A. D. Vo, A. S. Hafner, Competition for synaptic building blocks shapes synaptic plasticity. eLife **7**, e37836 (2018). 30222108 10.7554/eLife.37836 PMC6181566

[34] J. A. Hirsch , Functionally distinct inhibitory neurons at the first stage of visual cortical processing. Nat. Neurosci. **6**, 1300–1308 (2003). 14625553 10.1038/nn1152

[35] J. A. Cardin, L. A. Palmer, D. Contreras, Stimulus feature selectivity in excitatory and inhibitory neurons in primary visual cortex. J. Neurosci. **27**, 10333–10344 (2007). 17898205 10.1523/JNEUROSCI.1692-07.2007 PMC3025280

[36] C. A. Runyan , Response features of parvalbumin-expressing interneurons suggest precise roles for subtypes of inhibition in visual cortex. Neuron **67**, 847–857 (2010). 20826315 10.1016/j.neuron.2010.08.006 PMC2948796

[37] A. K. Moore, M. Wehr, Parvalbumin-expressing inhibitory interneurons in auditory cortex are well-tuned for frequency. J. Neurosci. **33**, 13713–13723 (2013). 23966693 10.1523/JNEUROSCI.0663-13.2013 PMC3755717

[38] E. Oja, “Learning in non-linear constrained Hebbian networks” in *Proceedings of the ICANN’91* (1991), pp. 385–390.

[39] G. Ocker, M. Buice, Tensor decompositions of higher-order correlations by nonlinear Hebbian plasticity. Adv. Neural Information Process. Syst. **34**, 11326–11339 (2021).

[40] A. J. Bell, T. J. Sejnowski, The “independent components’’ of natural scenes are edge filters. Vis. Res. **37**, 3327–3338 (1997). 9425547 10.1016/s0042-6989(97)00121-1 PMC2882863

[41] C. S. Brito, W. Gerstner, Nonlinear Hebbian learning as a unifying principle in receptive field formation. PLoS Comput. Biol. **12**, e1005070 (2016). 27690349 10.1371/journal.pcbi.1005070 PMC5045191

[42] J. A. D’Amour, R. C. Froemke, Inhibitory and excitatory spike-timing-dependent plasticity in the auditory cortex. Neuron **86**, 514–528 (2015). 25843405 10.1016/j.neuron.2015.03.014 PMC4409545

[43] F. Lagzi, M. C. Bustos, A. M. Oswald, B. Doiron, Assembly formation is stabilized by parvalbumin neurons and accelerated by somatostatin neurons. bioRxiv [Preprint] (2021). 10.1101/2021.09.06.459211 (Accessed 15 May 2024).

[44] P. Golshani , Internally mediated developmental desynchronization of neocortical network activity. J. Neurosci. **29**, 10890–10899 (2009). 19726647 10.1523/JNEUROSCI.2012-09.2009 PMC2771734

[45] N. L. Rochefort , Sparsification of neuronal activity in the visual cortex at eye-opening. Proc. Natl. Acad. Sci. U.S.A. **106**, 15049–15054 (2009). 19706480 10.1073/pnas.0907660106 PMC2736444

[46] H. N. Mulholland, B. Hein, M. Kaschube, G. B. Smith, Tightly coupled inhibitory and excitatory functional networks in the developing primary visual cortex. eLife **10**, e72456 (2021). 34878404 10.7554/eLife.72456 PMC8654369

[47] M. Chini, T. Pfeffer, I. Hanganu-Opatz, An increase of inhibition drives the developmental decorrelation of neural activity. eLife **11**, e78811 (2022). 35975980 10.7554/eLife.78811 PMC9448324

[48] C. D. Gilbert, T. N. Wiesel, Columnar specificity of intrinsic horizontal and corticocortical connections in cat visual cortex. J. Neurosci. **9**, 2432–2442 (1989). 2746337 10.1523/JNEUROSCI.09-07-02432.1989 PMC6569760

[49] Y. Yoshimura, J. L. Dantzker, E. M. Callaway, Excitatory cortical neurons form fine-scale functional networks. Nature **433**, 868–873 (2005). 15729343 10.1038/nature03252

[50] Y. Yoshimura, E. M. Callaway, Fine-scale specificity of cortical networks depends on inhibitory cell type and connectivity. Nat. Neurosci. **8**, 1552–1559 (2005). 16222228 10.1038/nn1565

[51] D. E. Wilson , Gabaergic neurons in ferret visual cortex participate in functionally specific networks. Neuron **93**, 1058–1065 (2017). 28279352 10.1016/j.neuron.2017.02.035 PMC5477844

[52] H. Ko , Functional specificity of local synaptic connections in neocortical networks. Nature **473**, 87–91 (2011). 21478872 10.1038/nature09880 PMC3089591

[53] A. D. Lien, M. Scanziani, Tuned thalamic excitation is amplified by visual cortical circuits. Nat. Neurosci. **16**, 1315–1323 (2013). 23933748 10.1038/nn.3488 PMC3774518

[54] Y. Li, L. A. Ibrahim, B. Liu, L. I. Zhang, H. W. Tao, Linear transformation of thalamocortical input by intracortical excitation. Nat. Neurosci. **16**, 1324–1330 (2013). 23933750 10.1038/nn.3494 PMC3855439

[55] L. Li, Y. Li, M. Zhou, H. W. Tao, L. I. Zhang, Intracortical multiplication of thalamocortical signals in mouse auditory cortex. Nat. Neurosci. **16**, 1179–1181 (2013). 23933752 10.1038/nn.3493 PMC3844430

[56] L. Cossell , Functional organization of excitatory synaptic strength in primary visual cortex. Nature **518**, 399–403 (2015). 25652823 10.1038/nature14182 PMC4843963

[57] M. F. Iacaruso, I. T. Gasler, S. B. Hofer, Synaptic organization of visual space in primary visual cortex. Nature **547**, 449–452 (2017). 28700575 10.1038/nature23019 PMC5533220

[58] P. Znamenskiy , Functional specificity of recurrent inhibition in visual cortex. Neuron **112**, 991–1000 (2024). 38244539 10.1016/j.neuron.2023.12.013 PMC7618320

[59] T. W. Troyer, A. E. Krukowski, N. J. Priebe, K. D. Miller, Contrast-invariant orientation tuning in cat visual cortex: Thalamocortical input tuning and correlation-based intracortical connectivity. J. Neurosci. **18**, 5908–5927 (1998). 9671678 10.1523/JNEUROSCI.18-15-05908.1998 PMC6793055

[60] J. S. Anderson, M. Carandini, D. Ferster, Orientation tuning of input conductance, excitation, and inhibition in cat primary visual cortex. J. Neurophysiol. **84**, 909–926 (2000). 10938316 10.1152/jn.2000.84.2.909

[61] L. M. Martinez, J. M. Alonso, R. C. Reid, J. A. Hirsch, Laminar processing of stimulus orientation in cat visual cortex. J. Physiol. **540**, 321–333 (2002). 11927690 10.1113/jphysiol.2001.012776 PMC2290204

[62] J. Mariño , Invariant computations in local cortical networks with balanced excitation and inhibition. Nat. Neurosci. **8**, 194–201 (2005). 15665876 10.1038/nn1391

[63] A. Y. Tan, B. D. Brown, B. Scholl, D. Mohanty, N. J. Priebe, Orientation selectivity of synaptic input to neurons in mouse and cat primary visual cortex. J. Neurosci. **31**, 12339–12350 (2011). 21865476 10.1523/JNEUROSCI.2039-11.2011 PMC3202243

[64] D. E. Wilson, B. Scholl, D. Fitzpatrick, Differential tuning of excitation and inhibition shapes direction selectivity in ferret visual cortex. Nature **560**, 97–101 (2018). 30046106 10.1038/s41586-018-0354-1 PMC6946183

[65] D. Rose, C. Blakemore, Effects of bicuculline on functions of inhibition in visual cortex. Nature **249**, 375–377 (1974). 4842746 10.1038/249375a0

[66] X. Pei, T. Vidyasagar, M. Volgushev, O. Creutzfeldt, Receptive field analysis and orientation selectivity of postsynaptic potentials of simple cells in cat visual cortex. J. Neurosci. **14**, 7130–7140 (1994). 7965103 10.1523/JNEUROSCI.14-11-07130.1994 PMC6577294

[67] C. Monier, F. Chavane, P. Baudot, L. J. Graham, Y. Frégnac, Orientation and direction selectivity of synaptic inputs in visual cortical neurons: A diversity of combinations produces spike tuning. Neuron **37**, 663–680 (2003). 12597863 10.1016/s0896-6273(03)00064-3

[68] G. K. Wu, R. Arbuckle, H. W. Bh Liu, LI Zhang. Tao, Lateral sharpening of cortical frequency tuning by approximately balanced inhibition. Neuron **58**, 132–143 (2008). 18400169 10.1016/j.neuron.2008.01.035 PMC2447869

[69] Bh. Liu , Broad inhibition sharpens orientation selectivity by expanding input dynamic range in mouse simple cells. Neuron **71**, 542–554 (2011). 21835349 10.1016/j.neuron.2011.06.017 PMC3154747

[70] Y. Li, B. Liu, X. Chou, L. I. Zhang, H. W. Tao, Synaptic basis for differential orientation selectivity between complex and simple cells in mouse visual cortex. J. Neurosci. **35**, 11081–11093 (2015). 26245969 10.1523/JNEUROSCI.5246-14.2015 PMC4524977

[71] L. Busse, A. R. Wade, M. Carandini, Representation of concurrent stimuli by population activity in visual cortex. Neuron **64**, 931–942 (2009). 20064398 10.1016/j.neuron.2009.11.004 PMC2807406

[72] S. P. MacEvoy, T. R. Tucker, D. Fitzpatrick, A precise form of divisive suppression supports population coding in the primary visual cortex. Nat. Neurosci. **12**, 637–645 (2009). 19396165 10.1038/nn.2310 PMC2875123

[73] B. Li, J. K. Thompson, T. Duong, M. R. Peterson, R. D. Freeman, Origins of cross-orientation suppression in the visual cortex. J. Neurophysiol. **96**, 1755–1764 (2006). 16855109 10.1152/jn.00425.2006

[74] N. J. Priebe, D. Ferster, Mechanisms underlying cross-orientation suppression in cat visual cortex. Nat. Neurosci. **9**, 552–561 (2006). 16520737 10.1038/nn1660

[75] D. Barbera, N. J. Priebe, L. L. Glickfeld, Feedforward mechanisms of cross-orientation interactions in mouse v1. Neuron **110**, 297–311 (2022). 34735779 10.1016/j.neuron.2021.10.017 PMC8920535

[76] F. Sengpiel, V. Vorobyov, Intracortical origins of interocular suppression in the visual cortex. J. Neurosci. **25**, 6394–6400 (2005). 16000630 10.1523/JNEUROSCI.0862-05.2005 PMC6725266

[77] Y. Ahmadian, D. B. Rubin, K. D. Miller, Analysis of the stabilized supralinear network. Neural Comp. **25**, 1994–2037 (2013). 10.1162/NECO_a_00472 PMC4026108 23663149

[78] T. C. Freeman, S. Durand, D. C. Kiper, M. Carandini, Suppression without inhibition in visual cortex. Neuron **35**, 759–771 (2002). 12194874 10.1016/s0896-6273(02)00819-x

[79] C. Blakemore, E. A. Tobin, Lateral inhibition between orientation detectors in the cat’s visual cortex. Exp. Brain Res. **15**, 439–440 (1972). 5079475 10.1007/BF00234129

[80] J. J. Knierim, D. C. Van Essen, Neuronal responses to static texture patterns in area v1 of the alert macaque monkey. J. Neurophysiol. **67**, 961–980 (1992). 1588394 10.1152/jn.1992.67.4.961

[81] J. R. Cavanaugh, W. Bair, J. A. Movshon, Selectivity and spatial distribution of signals from the receptive field surround in macaque v1 neurons. J. Neurophysiol. **88**, 2547–2556 (2002). 12424293 10.1152/jn.00693.2001

[82] B. S. Webb, N. T. Dhruv, S. G. Solomon, C. Tailby, P. Lennie, Early and late mechanisms of surround suppression in striate cortex of macaque. J. Neurosci. **25**, 11666–11675 (2005). 16354925 10.1523/JNEUROSCI.3414-05.2005 PMC6726034

[83] H. Adesnik, M. Scanziani, Lateral competition for cortical space by layer-specific horizontal circuits. Nature **464**, 1155–1160 (2010). 20414303 10.1038/nature08935 PMC2908490

[84] M. Pecka, Y. Han, E. Sader, T. D. Mrsic-Flogel, Experience-dependent specialization of receptive field surround for selective coding of natural scenes. Neuron **84**, 457–469 (2014). 25263755 10.1016/j.neuron.2014.09.010 PMC4210638

[85] S. V. David, W. E. Vinje, J. L. Gallant, Natural stimulus statistics alter the receptive field structure of v1 neurons. J. Neurosci. **24**, 6991–7006 (2004). 15295035 10.1523/JNEUROSCI.1422-04.2004 PMC6729594

[86] G. Felsen, J. Touryan, F. Han, Y. Dan, Cortical sensitivity to visual features in natural scenes. PLoS Biol. **3**, e342 (2005). 16171408 10.1371/journal.pbio.0030342 PMC1233414

[87] G. Felsen, J. Touryan, Y. Dan, Contextual modulation of orientation tuning contributes to efficient processing of natural stimuli. Network: Comput. Neural Syst. **16**, 139–149 (2005). 10.1080/09548980500463347 16411493

[88] E. Froudarakis , Population code in mouse v1 facilitates readout of natural scenes through increased sparseness. Nat. Neurosci. **17**, 851–857 (2014). 24747577 10.1038/nn.3707 PMC4106281

[89] O. Schwartz, E. P. Simoncelli, Natural signal statistics and sensory gain control. Nat. Neurosci. **4**, 819–825 (2001). 11477428 10.1038/90526

[90] P. Berkes, G. Orbán, M. Lengyel, J. Fiser, Spontaneous cortical activity reveals hallmarks of an optimal internal model of the environment. Science **331**, 83–87 (2011). 21212356 10.1126/science.1195870 PMC3065813

[91] M. Zhu, C. J. Rozell, Visual nonclassical receptive field effects emerge from sparse coding in a dynamical system. PLoS Comput. Biol. **9**, e1003191 (2013). 24009491 10.1371/journal.pcbi.1003191 PMC3757072

[92] M. F. Burg , Learning divisive normalization in primary visual cortex. PLoS Comput. Biol. **17**, e1009028 (2021). 34097695 10.1371/journal.pcbi.1009028 PMC8211272

[93] V. Veerabadran, R. Raina, V. R. de Sa, “Bio-inspired learnable divisive normalization for ANNs” in *SVRHM 2021 Workshop NeurIPS* (2021).

[94] J. Fu , Pattern completion and disruption characterize contextual modulation in mouse visual cortex. bioRxiv [Preprint] (2023). 10.1101/2023.03.13.532473 (Accessed 15 May 2024).

[95] E. L. Bienenstock, L. N. Cooper, P. W. Munro, Theory for the development of neuron selectivity: Orientation specificity and binocular interaction in visual cortex. J. Neurosci. **2**, 32–48 (1982). 7054394 10.1523/JNEUROSCI.02-01-00032.1982 PMC6564292

[96] F. I. Kleberg, T. Fukai, M. Gilson, Excitatory and inhibitory STDP jointly tune feedforward neural circuits to selectively propagate correlated spiking activity. Front. Comput. Neurosci. **8**, 53 (2014). 24847242 10.3389/fncom.2014.00053 PMC4019846

[97] F. Effenberger, J. Jost, A. Levina, Self-organization in balanced state networks by STDP and homeostatic plasticity. PLoS Comput. Biol. **11**, e1004420 (2015). 26335425 10.1371/journal.pcbi.1004420 PMC4559467

[98] S. Sadeh, C. Clopath, S. Rotter, Emergence of functional specificity in balanced networks with synaptic plasticity. PLoS Comput. Biol. **11**, e1004307 (2015). 26090844 10.1371/journal.pcbi.1004307 PMC4474917

[99] J. Aljadeff, J. D’amour, R. E. Field, R. C. Froemke, C. Clopath, Cortical credit assignment by Hebbian, neuromodulatory and inhibitory plasticity. arXiv [Preprint] (2019). https://arxiv.org/abs/1911.00307 (Accessed 15 May 2024).

[100] V. Pedrosa, C. Clopath, Voltage-based inhibitory synaptic plasticity: Network regulation, diversity, and flexibility. bioRxiv [Preprint] (2020). 10.1101/2020.12.08.416263 (Accessed 15 May 2024).

[101] C. Miehl, J. Gjorgjieva, Stability and learning in excitatory synapses by nonlinear inhibitory plasticity. PLoS Comput. Biol. **18**, e1010682 (2022). 36459503 10.1371/journal.pcbi.1010682 PMC9718420

[102] E. J. Agnes, T. P. Vogels, Co-dependent excitatory and inhibitory plasticity accounts for quick, stable and long-lasting memories in biological networks. Nat. Neurosci. **27**, 964–974 (2024). 38509348 10.1038/s41593-024-01597-4 PMC11089004

[103] T. P. Vogels , Inhibitory synaptic plasticity: Spike timing-dependence and putative network function. Front. Neural Circuits **7**, 119 (2013). 23882186 10.3389/fncir.2013.00119 PMC3714539

[104] H. Sprekeler, Functional consequences of inhibitory plasticity: Homeostasis, the excitation-inhibition balance and beyond. Curr. Opin. Neurobiol. **43**, 198–203 (2017). 28500933 10.1016/j.conb.2017.03.014

[105] Y. K. Wu, C. Miehl, J. Gjorgjieva, Regulation of circuit organization and function through inhibitory synaptic plasticity. Trends Neurosci. **45**, 884–898 (2022). 36404455 10.1016/j.tins.2022.10.006

[106] C. Miehl, S. Onasch, D. Festa, J. Gjorgjieva, Formation and computational implications of assemblies in neural circuits. J. Physiol. **601**, 3071–3090 (2023). 36068723 10.1113/JP282750

[107] C. Pehlevan, A. M. Sengupta, D. B. Chklovskii, Why do similarity matching objectives lead to Hebbian/anti-Hebbian networks? Neural Comput. **30**, 84–124 (2017). 28957017 10.1162/neco_a_01018

[108] C. Von der Malsburg, Self-organization of orientation sensitive cells in the striate cortex. Kybernetik **14**, 85–100 (1973). 4786750 10.1007/BF00288907

[109] V. Delattre, D. Keller, M. Perich, H. Markram, E. B. Muller, Network-timing-dependent plasticity. Front. Cell. Neurosci. **9**, 220 (2015). 26106298 10.3389/fncel.2015.00220 PMC4460533

[110] T. Magchielse, E. Meeter, The effect of neuronal activity on the competitive elimination of neuromuscular junctions in tissue culture. Dev. Brain Res. **25**, 211–220 (1986). 10.1016/s0006-8993(86)80229-3 3955371

[111] P. G. Nelson, C. Yu, R. D. Fields, E. A. Neale, Synaptic connections in vitro: Modulation of number and efficacy by electrical activity. Science **244**, 585–587 (1989). 2717942 10.1126/science.2717942

[112] Y. J. Lo, M. M. Poo, Activity-dependent synaptic competition in vitro: Heterosynaptic suppression of developing synapses. Science **254**, 1019–1022 (1991). 1658939 10.1126/science.1658939

[113] M. Scanziani, R. C. Malenka, R. A. Nicoll, Role of intercellular interactions in heterosynaptic long-term depression. Nature **380**, 446–450 (1996). 8602244 10.1038/380446a0

[114] S. Royer, D. Paré, Conservation of total synaptic weight through balanced synaptic depression and potentiation. Nature **422**, 518–522 (2003). 12673250 10.1038/nature01530

[115] R. Fonseca, U. V. Nägerl, R. G. Morris, T. Bonhoeffer, Competing for memory: Hippocampal LTP under regimes of reduced protein synthesis. Neuron **44**, 1011–1020 (2004). 15603743 10.1016/j.neuron.2004.10.033

[116] I. Rabinowitch, I. Segev, Two opposing plasticity mechanisms pulling a single synapse. Trends Neurosci. **31**, 377–383 (2008). 18602704 10.1016/j.tins.2008.05.005

[117] A. Govindarajan, I. Israely, S. Y. Huang, S. Tonegawa, The dendritic branch is the preferred integrative unit for protein synthesis-dependent LTP. Neuron **69**, 132–146 (2011). 21220104 10.1016/j.neuron.2010.12.008 PMC3032443

[118] W. C. Oh, L. K. Parajuli, K. Zito, Heterosynaptic structural plasticity on local dendritic segments of hippocampal CA1 neurons. Cell Rep. **10**, 162–169 (2015). 25558061 10.1016/j.celrep.2014.12.016 PMC4294981

[119] S. El-Boustani , Locally coordinated synaptic plasticity of visual cortex neurons in vivo. Science **360**, 1349–1354 (2018). 29930137 10.1126/science.aao0862 PMC6366621

[120] G. Antunes, F. Simoes-de Souza, AMPA receptor trafficking and its role in heterosynaptic plasticity. Sci. Rep. **8**, 1–14 (2018). 29985438 10.1038/s41598-018-28581-w PMC6037747

[121] A. Perez-Alvarez , Endoplasmic reticulum visits highly active spines and prevents runaway potentiation of synapses. Nat. Commun. **11**, 1–10 (2020). 33033259 10.1038/s41467-020-18889-5 PMC7546627

[122] T. Ravasenga , Spatial regulation of coordinated excitatory and inhibitory synaptic plasticity at dendritic synapses. Cell Rep. **38**, 110347 (2022). 35139381 10.1016/j.celrep.2022.110347 PMC8844559

[123] N. W. Gray, R. M. Weimer, I. Bureau, K. Svoboda, Rapid redistribution of synaptic PSD-95 in the neocortex in vivo. PLoS Biol. **4**, e370 (2006). 17090216 10.1371/journal.pbio.0040370 PMC1634879

[124] S. H. Lee , Super-resolution imaging of synaptic and extra-synaptic AMPA receptors with different-sized fluorescent probes. eLife **6**, e27744 (2017). 28749340 10.7554/eLife.27744 PMC5779237

[125] M. Sheng, E. Kim, The postsynaptic organization of synapses. Cold Spring Harb. Persp. Biol. **3**, a005678 (2011). 10.1101/cshperspect.a005678 PMC3225953 22046028

[126] M. van Oostrum , The proteomic landscape of synaptic diversity across brain regions and cell types. Cell **186**, 5411–5427 (2023). 37918396 10.1016/j.cell.2023.09.028 PMC10686415

[127] A. Gupta, Y. Wang, H. Markram, Organizing principles for a diversity of GABAergic interneurons and synapses in the neocortex. Science **287**, 273–278 (2000). 10634775 10.1126/science.287.5451.273

[128] A. M. Craig, H. Boudin, Molecular heterogeneity of central synapses: Afferent and target regulation. Nat. Neurosci. **4**, 569–578 (2001). 11369937 10.1038/88388

[129] G. H. Diering, R. L. Huganir, The AMPA receptor code of synaptic plasticity. Neuron **100**, 314–329 (2018). 30359599 10.1016/j.neuron.2018.10.018 PMC6214363

[130] J. J. Zhu, Activity level-dependent synapse-specific AMPA receptor trafficking regulates transmission kinetics. J. Neurosci. **29**, 6320–6335 (2009). 19439609 10.1523/JNEUROSCI.4630-08.2009 PMC2734326

[131] J. A. Wen, A. L. Barth, Input-specific critical periods for experience-dependent plasticity in layer 2/3 pyramidal neurons. J. Neurosci. **31**, 4456–4465 (2011). 21430146 10.1523/JNEUROSCI.6042-10.2011 PMC3066457

[132] J. N. Levinson, A. El-Husseini, Building excitatory and inhibitory synapses: Balancing neuroligin partnerships. Neuron **48**, 171–174 (2005). 16242398 10.1016/j.neuron.2005.09.017

[133] A. A. Chubykin , Activity-dependent validation of excitatory versus inhibitory synapses by neuroligin-1 versus neuroligin-2. Neuron **54**, 919–931 (2007). 17582332 10.1016/j.neuron.2007.05.029 PMC3738748

[134] M. W. Self, R. N. Kooijmans, H. Supèr, V. A. Lamme, P. R. Roelfsema, Different glutamate receptors convey feedforward and recurrent processing in macaque v1. Proc. Natl. Acad. Sci. U.S.A. **109**, 11031–11036 (2012). 22615394 10.1073/pnas.1119527109 PMC3390882

[135] M. E. Horn, R. A. Nicoll, Somatostatin and parvalbumin inhibitory synapses onto hippocampal pyramidal neurons are regulated by distinct mechanisms. Proc. Natl. Acad. Sci. U.S.A. **115**, 589–594 (2018). 29295931 10.1073/pnas.1719523115 PMC5777005

[136] C. Bernard , Cortical wiring by synapse type-specific control of local protein synthesis. Science **378**, eabm7466 (2022). 36423280 10.1126/science.abm7466 PMC7618116

[137] R. S. Larsen, P. J. Sjöström, Synapse-type-specific plasticity in local circuits. Curr. Opin. Neurobiol. **35**, 127–135 (2015). 26310110 10.1016/j.conb.2015.08.001 PMC5280068

[138] A. R. McFarlan , The plasticitome of cortical interneurons. Nat. Rev. Neurosci. **24**, 80–97 (2022). 36585520 10.1038/s41583-022-00663-9

[139] G. G. Turrigiano, K. R. Leslie, N. S. Desai, L. C. Rutherford, S. B. Nelson, Activity-dependent scaling of quantal amplitude in neocortical neurons. Nature **391**, 892–896 (1998). 9495341 10.1038/36103

[140] G. G. Turrigiano, S. B. Nelson, Homeostatic plasticity in the developing nervous system. Nat. Rev. Neurosci. **5**, 97–107 (2004). 14735113 10.1038/nrn1327

[141] P. Wenner, Mechanisms of GABAergic homeostatic plasticity. Neural Plast. **2011**, 489470 (2011). 21876819 10.1155/2011/489470 PMC3159121

[142] I. Spiegel , Npas4 regulates excitatory-inhibitory balance within neural circuits through cell-type-specific gene programs. Cell **157**, 1216–1229 (2014). 24855953 10.1016/j.cell.2014.03.058 PMC4089405

[143] G. G. Turrigiano, The dialectic of Hebb and homeostasis. Philos. Trans. R. Soc. B: Biol. Sci. **372**, 20160258 (2017). 10.1098/rstb.2016.0258 PMC5247594 28093556

[144] F. Zenke, W. Gerstner, S. Ganguli, The temporal paradox of Hebbian learning and homeostatic plasticity. Curr. Opin. Neurobiol. **43**, 166–176 (2017). 28431369 10.1016/j.conb.2017.03.015

[145] N. Kraynyukova, T. Tchumatchenko, Stabilized supralinear network can give rise to bistable, oscillatory, and persistent activity. Proc. Natl. Acad. Sci. U.S.A. **115**, 3464–3469 (2018). 29531035 10.1073/pnas.1700080115 PMC5879648

[146] J. T. Chang, D. Fitzpatrick, Development of visual response selectivity in cortical GABAergic interneurons. Nat. Commun. **13**, 1–14 (2022). 35778379 10.1038/s41467-022-31284-6 PMC9249896

[147] T. K. Hensch, Critical period plasticity in local cortical circuits. Nat. Rev. Neurosci. **6**, 877–888 (2005). 16261181 10.1038/nrn1787

[148] C. N. Levelt, M. Hübener, Critical-period plasticity in the visual cortex. Annu. Rev. Neurosci. **35**, 309–330 (2012). 22462544 10.1146/annurev-neuro-061010-113813

[149] A. Peters, B. R. Payne, Numerical relationships between geniculocortical afferents and pyramidal cell modules in cat primary visual cortex. Cereb. Cortex **3**, 69–78 (1993). 8439740 10.1093/cercor/3.1.69

[150] A. Peters, B. R. Payne, J. Budd, A numerical analysis of the geniculocortical input to striate cortex in the monkey. Cereb. Cortex **4**, 215–229 (1994). 8075528 10.1093/cercor/4.3.215

[151] R. J. Douglas, K. A. Martin, Neuronal circuits of the neocortex. Annu. Rev. Neurosci. **27**, 419–451 (2004). 15217339 10.1146/annurev.neuro.27.070203.144152

[152] S. Lefort, C. Tomm, J. C. F. Sarria, C. C. Petersen, The excitatory neuronal network of the C2 barrel column in mouse primary somatosensory cortex. Neuron **61**, 301–316 (2009). 19186171 10.1016/j.neuron.2008.12.020

[153] V. Braitenberg, A. Schüz, Cortex: Statistics and Geometry of Neuronal Connectivity (Springer Science & Business Media, 2013).

[154] G. H. Seol , Neuromodulators control the polarity of spike-timing-dependent synaptic plasticity. Neuron **55**, 919–929 (2007). 17880895 10.1016/j.neuron.2007.08.013 PMC2756178

[155] V. Pawlak, J. R. Wickens, A. Kirkwood, J. N. Kerr, Timing is not everything: Neuromodulation opens the STDP gate. Front. Synaptic Neurosci. **2**, 146 (2010). 21423532 10.3389/fnsyn.2010.00146 PMC3059689

[156] R. C. Froemke, Plasticity of cortical excitatory-inhibitory balance. Annu. Rev. Neurosci. **38**, 195–219 (2015). 25897875 10.1146/annurev-neuro-071714-034002 PMC4652600

[157] Z. Brzosko, S. B. Mierau, O. Paulsen, Neuromodulation of spike-timing-dependent plasticity: Past, present, and future. Neuron **103**, 563–581 (2019). 31437453 10.1016/j.neuron.2019.05.041

[158] A. A. Disney, Neuromodulatory control of early visual processing in macaque. Annu. Rev. Vision Sci. **7**, 181–199 (2021). 10.1146/annurev-vision-100119-125739 34524875

[159] A. Maffei, G. Turrigiano, The age of plasticity: Developmental regulation of synaptic plasticity in neocortical microcircuits. Progr. Brain Res. **169**, 211–223 (2008). 10.1016/S0079-6123(07)00012-X 18394476

[160] A. E. Takesian, T. K. Hensch, Balancing plasticity/stability across brain development. Progr. Brain Res. **207**, 3–34 (2013). 10.1016/B978-0-444-63327-9.00001-1 24309249

[161] H. Ko , The emergence of functional microcircuits in visual cortex. Nature **496**, 96–100 (2013). 23552948 10.1038/nature12015 PMC4843961

[162] D. H. Hubel, T. N. Wiesel, Receptive fields of cells in striate cortex of very young, visually inexperienced kittens. J. Neurophysiol. **26**, 994–1002 (1963). 14084171 10.1152/jn.1963.26.6.994

[163] T. N. Wiesel, D. H. Hubel, Ordered arrangement of orientation columns in monkeys lacking visual experience. J. Comp. Neurol. **158**, 307–318 (1974). 4215829 10.1002/cne.901580306

[164] B. Chapman, M. P. Stryker, Development of orientation selectivity in ferret visual cortex and effects of deprivation. J. Neurosci. **13**, 5251–5262 (1993). 8254372 10.1523/JNEUROSCI.13-12-05251.1993 PMC6576418

[165] J. B. Ackman, T. J. Burbridge, M. C. Crair, Retinal waves coordinate patterned activity throughout the developing visual system. Nature **490**, 219–225 (2012). 23060192 10.1038/nature11529 PMC3962269

[166] A. Thompson, A. Gribizis, C. Chen, M. C. Crair, Activity-dependent development of visual receptive fields. Curr. Opin. Neurobiol. **42**, 136–143 (2017). 28088066 10.1016/j.conb.2016.12.007 PMC5375035

[167] F. J. Martini, T. Guillamón-Vivancos, V. Moreno-Juan, M. Valdeolmillos, G. López-Bendito, Spontaneous activity in developing thalamic and cortical sensory networks. Neuron **109**, 2519–2534 (2021). 34293296 10.1016/j.neuron.2021.06.026 PMC7611560

[168] S. Eckmann, Synapse-type-specific competitive Hebbian learning. GitHub. https://github.com/comp-neural-circuits/Synapse-type-specific-competitive-Hebbian-learning. Accessed 15 May 2024. 10.1073/pnas.2305326121 PMC11194505 38870059

