# Supplementary material for: Synapse-type-specific competitive Hebbian learning forms functional recurrent networks
Source: Proc Natl Acad Sci U S A. 2024 Jun 13;121(25):e2305326121. doi: 10.1073/pnas.2305326121 (PMC11194505; doi:10.1073/pnas.2305326121)
Supplement: Supplementary file 1 — Appendix 01 (PDF) [file pnas.2305326121.sapp.pdf]

Supplementary Material for

Synapse-type-specific competitive Hebbian learning forms functional recurrent networks

Samuel Eckmann ✉[,1,2], **Edward James Young**[2], **and Julijana Gjorgjieva**[1,3]

[1]Max Planck Institute for Brain Research, Frankfurt am Main, Germany
[2]Computational and Biological Learning Lab, University of Cambridge, Cambridge, United Kingdom
[3]School of Life Sciences, Technical University Munich, Freising, Germany
✉Corresponding author, Email: ec.sam@outlook.com

Contents

1 Linear competitive Hebbian learning finds principal components **2**
1.1 Hebbian plasticity without normalization is unstable . . . 2
1.2 Weight constraints stabilize unlimited Hebbian growth . . . 3
1.2.1 Fixed points . . . 4
1.2.2 Stability analysis . . . 4
1.2.3 Classic Inhibitory plasticity prevents stimulus selectivity . . . 5

2 Synapse-type-specific normalization balances E-I receptive fields **6**
2.1 Fixed points . . . 7
2.1.1 Eigenvectors of the modified covariance matrix are fixed points . . . 7
2.1.2 Eigenvectors and eigenvalues of the modified covariance matrix . . . 8
2.1.3 Non-eigenvector fixed points . . . 8
2.1.4 General fixed points . . . 9
2.2 Stability analysis . . . 10
2.2.1 Principal component analysis in inhibition modified input space . . . 13
2.2.2 Fast inhibition increases stability . . . 13
2.2.3 Stability of non-eigenvector fixed points . . . 14

3 Lateral input stretches and compresses the feedforward input space **15**

4 Eigencircuits **19**
4.1 Variance propagation . . . 19
4.2 Consistency conditions provide eigencircuit firing rate variances . . . 20
4.3 A note on the choice of weight norm . . . 21

5 E-I networks with fully plastic recurrent connectivity **22**
5.1 Fixed points . . . 24
5.2 Stability analysis . . . 25
5.2.1 The transformed Jacobian . . . 26
5.2.2 Stability conditions . . . 29
5.2.3 Eigencircuit stability depends on recurrent connectivity . . . 30
5.2.4 Decorrelation condition . . . 32

6 Movie captions **32**

1 Linear competitive Hebbian learning finds principal components

Before considering inhibitory plasticity, we recapitulate how linear Hebbian learning finds the principal eigenvector of a neuron's inputs. Although first described by Oja (*1*), we will mostly follow the derivation by Miller and MacKay (2) that we will later extend to inhibitory neurons.

1.1 Hebbian plasticity without normalization is unstable

We consider a single neuron that receives input from a set of excitatory neurons (Fig. S1*A*). Its output firing rate r is a weighted sum of the firing rates of its pre-synaptic inputs $\mathbf{y}$. One can conveniently write this as a dot product:

$$\tau_r \dot{r} = -r + \sum_i w_i y_i = -r + \mathbf{w}^\mathsf{T} \mathbf{y}, \tag{1}$$

where $\mathbf{w}$ is a vector that holds the synaptic weights, and τ_r defines the timescale at which the activity changes. In the following, lowercase letters in bold indicate vectors, and uppercase letters in bold matrices. Following Hebb's principle, synaptic weight changes depend on the pre- and post-synaptic firing rates. In vector notation:

$$\boldsymbol{\tau} \dot{\mathbf{w}} = \mathbf{y} r \tag{2}$$

where the constant τ sets the timescale of plasticity. Assuming that synaptic weights change on a much slower timescale than firing rates, $\tau_r \ll \tau$, we make the simplifying assumption that r reaches its fixed point instantaneously, i.e., $\tau_r \ll 1$ and $r = \mathbf{w}^\mathsf{T} \mathbf{y}$, and consider the same plasticity timescale for all synapses $\boldsymbol{\tau} = \mathbb{1}$. Then, the average change of the synaptic weights can be expressed as a linear transformation of the original weight vector:

$$\langle \dot{\mathbf{w}} \rangle = \langle \mathbf{y} r \rangle = \langle \mathbf{y}\mathbf{y}^T \mathbf{w} \rangle = \mathbf{C}\boldsymbol{w}, \quad \boldsymbol{C} \equiv \langle \boldsymbol{y}\boldsymbol{y}^\mathsf{T} \rangle, \tag{3}$$

where $\langle \cdot \rangle$ is a temporal average and $\boldsymbol{C}$ is the covariance matrix of the synaptic inputs $\mathbf{y}$, assuming inputs have zero mean, $\langle \mathbf{y} \rangle = \mathbf{0}$. In the following, we only consider the average weight changes and omit the angled notation for convenience. To solve this differential equation, we express the weight change in the eigenvector basis of the covariance matrix $\mathbf{C}$, which is symmetric and positive-semidefinite and, therefore, has a complete set of orthonormal eigenvectors with non-negative eigenvalues.

$$\dot{\mathbf{w}}_v \equiv \mathbf{V}^{-1} \dot{\mathbf{w}} = \mathbf{V}^\mathsf{T} \mathbf{C} \mathbf{V} \mathbf{V}^\mathsf{T} \mathbf{w} = \boldsymbol{\Lambda} \mathbf{w}_v, \tag{4}$$

$$\Rightarrow \boldsymbol{w}_v = \exp(\boldsymbol{\Lambda} t) \boldsymbol{w}_v(t_0). \tag{5}$$

Here, $\boldsymbol{\Lambda}$ is the diagonal eigenvalue matrix, and each column of $\mathbf{V}$ holds mutually orthogonal eigenvectors, i.e., $\mathbf{V}\mathbf{V}^\mathsf{T} = \mathbb{1}$, and $\mathbf{V}^{-1} = \mathbf{V}^\mathsf{T}$. Each eigenvector component grows exponentially at a rate given by the respective eigenvalue, which we identify with the *attraction* of the input component. We call eigenvector components with positive eigenvalue *attractive*, and the eigenvector component with the largest eigenvalue the *most attractive* input mode. We will later see that eigenvalues that describe the dynamics of input modes can become negative (Sec. 2). We will call such input modes with negative corresponding eigenvalue *repulsive*.

In summary, we find that unconstrained Hebbian plasticity results in the unlimited growth of synaptic weights and is therefore unstable. One way to constrain this unlimited growth is to modify the Hebbian learning rule such that the total synaptic weight is maintained.

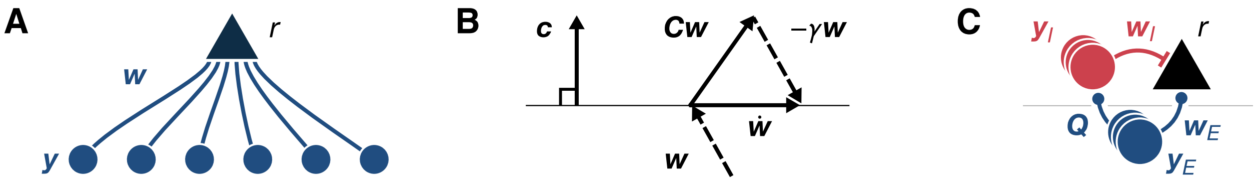

Figure S1: (*A*) Feedforward excitatory circuit. A post-synaptic neuron with output firing rate r receives synapses $\boldsymbol{w}$ from a set of excitatory neurons with firing rates y_E. (*B*) The normalization operation constrains synaptic weight changes $\dot{\boldsymbol{w}}$ to a hyperplane that is perpendicular to the constraint vector $\boldsymbol{c}$ by subtracting a multiple γ of the weight vector $\boldsymbol{w}$. See text for details. Figure adapted from Miller and MacKay (2). (*C*) Feedforward inhibitory circuit. A post-synaptic neuron with output firing rate r receives excitatory synapses $\boldsymbol{w}_E$ from a population of N_E excitatory neurons with firing rates $\boldsymbol{y}_E$, and inhibitory synapses $\boldsymbol{w}_I$ from a population of N_I inhibitory neurons with firing rates $\boldsymbol{y}_I$. The gray horizontal line indicates the separation between two hypothetical brain regions or cortical layers.

1.2 Weight constraints stabilize unlimited Hebbian growth

Hebbian plasticity and weight normalization can be considered as two discrete steps. First, growing weights according to the Hebbian rule. Second, normalizing to maintain the total synaptic weight. In this section, we will follow Miller and MacKay (2) and show how one can integrate these two discrete steps into one and derive the effective weight change $\dot{\boldsymbol{w}}$. One can write the two steps as

$$\tilde{\boldsymbol{w}} = \boldsymbol{w}(t) + \boldsymbol{C}\boldsymbol{w}\Delta t, \quad \boldsymbol{w}(t+\Delta t) = \frac{W}{\boldsymbol{c}^{\mathsf{T}}\tilde{\boldsymbol{w}}}\tilde{\boldsymbol{w}}, \quad W \equiv \boldsymbol{c}^{\mathsf{T}}\boldsymbol{w}(t), \tag{6}$$

This update rule maintains the projection of $\boldsymbol{w}$ onto the constraint vector $\boldsymbol{c}$ by multiplicatively scaling the weight vector after the Hebbian learning step, i.e., $\tilde{\boldsymbol{w}}$. Alternatively, if we let W be a constant (cf. Eq. 6), the projection onto $\boldsymbol{c}$ would be constrained to be equal to that constant. In the following, we instead assume that the weights are already properly normalized and set the projection value as it was before the plasticity timestep, i.e., equal to W as defined above.

$$\boldsymbol{w}(t+\Delta t) = \beta\left[\boldsymbol{w}(t) + \boldsymbol{C}\boldsymbol{w}(t)\Delta t\right], \quad \beta(\boldsymbol{w}(t), \Delta t) = \frac{\boldsymbol{c}^{\mathsf{T}}\boldsymbol{w}(t)}{\boldsymbol{c}^{\mathsf{T}}\left[\boldsymbol{C}\boldsymbol{w}(t)\Delta t + \boldsymbol{w}(t)\right]}, \tag{7}$$

where β describes the multiplicative normalization that depends on the size of the timestep Δt and the previous weight $w(t)$. It is straightforward to check that the projection of the weight vector onto the constraint vector $\boldsymbol{c}$ does not change, i.e.,

$$\boldsymbol{c}^{\mathsf{T}}\boldsymbol{w}(t+\Delta t) = \boldsymbol{c}^{\mathsf{T}}\boldsymbol{w}(t). \tag{8}$$

Then, the effective weight change $\dot{\boldsymbol{w}}$ is given as

$$\dot{\boldsymbol{w}} = \lim_{\Delta t\to 0}\frac{\boldsymbol{w}(t+\Delta t) - \boldsymbol{w}(t)}{\Delta t} = \lim_{\Delta t\to 0}\left[\frac{\beta - 1}{\Delta t}\boldsymbol{w}(t) + \beta\boldsymbol{C}\boldsymbol{w}(t)\right] \tag{9}$$

$$= \lim_{\Delta t\to 0}\left[\frac{\beta - 1}{\Delta t}\boldsymbol{w}(t) + \beta\boldsymbol{C}\boldsymbol{w}(t) + \boldsymbol{C}\boldsymbol{w}(t) - \boldsymbol{C}\boldsymbol{w}(t)\right] \tag{10}$$

$$= \lim_{\Delta t\to 0}\left[\boldsymbol{C}\boldsymbol{w}(t) - \frac{1-\beta}{\Delta t}\left[\boldsymbol{w}(t) + \boldsymbol{C}\boldsymbol{w}(t)\Delta t\right]\right] \tag{11}$$

$$= \lim_{\Delta t\to 0}\left[\boldsymbol{C}\boldsymbol{w}(t) - \frac{1-\beta}{\beta\Delta t}\boldsymbol{w}(t+\Delta t)\right], \tag{12}$$

where, in the first and last steps, we used the definition of $\boldsymbol{w}(t+\Delta t)$ in Eq. 7. Next, we take the limit

$$\lim_{\Delta t\to 0}\frac{1-\beta}{\beta\Delta t} = \lim_{\Delta t\to 0}\frac{1}{\Delta t}\left(\frac{1}{\beta} - 1\right) \tag{13}$$

$$= \lim_{\Delta t\to 0}\frac{1}{\Delta t}\left(\frac{\boldsymbol{c}^{\mathsf{T}}\left[\boldsymbol{C}\boldsymbol{w}\Delta t + \boldsymbol{w}\right]}{\boldsymbol{c}^{\mathsf{T}}\boldsymbol{w}} - 1\right) \tag{14}$$

$$= \lim_{\Delta t\to 0}\frac{\boldsymbol{c}^{\mathsf{T}}\boldsymbol{C}\boldsymbol{w}}{\boldsymbol{c}^{\mathsf{T}}\boldsymbol{w}} + \frac{\boldsymbol{c}^{\mathsf{T}}\boldsymbol{w}}{\boldsymbol{c}^{\mathsf{T}}\boldsymbol{w}\Delta t} - \frac{1}{\Delta t} = \frac{\boldsymbol{c}^{\mathsf{T}}\boldsymbol{C}\boldsymbol{w}}{\boldsymbol{c}^{\mathsf{T}}\boldsymbol{w}}. \tag{15}$$

In summary, we get (cf. Fig. S1*B*):

$$\boxed{\Rightarrow \dot{\boldsymbol{w}} = \boldsymbol{C}\boldsymbol{w} - \gamma\boldsymbol{w}, \quad \gamma \equiv \frac{\boldsymbol{c}^{\mathsf{T}}\boldsymbol{C}\boldsymbol{w}}{\boldsymbol{c}^{\mathsf{T}}\boldsymbol{w}}.} \tag{16}$$

Here, γ is a scalar normalization factor that depends on the current weight $\boldsymbol{w}$.

An alternative way to derive $\dot{\boldsymbol{w}}$ is to guess the shape of the multiplicative normalization term in Eq. 16 and require that the change along the constraint vector is zero[1], i.e.,

$$\frac{\mathrm{d}}{\mathrm{d}t}\left(\boldsymbol{c}^{\mathsf{T}}\boldsymbol{w}\right) = \boldsymbol{c}^{\mathsf{T}}\dot{\boldsymbol{w}} = \boldsymbol{c}^{\mathsf{T}}\boldsymbol{C}\boldsymbol{w} - \gamma\boldsymbol{c}^{\mathsf{T}}\boldsymbol{w} \overset{!}{=} 0, \quad \Rightarrow \gamma = \frac{\boldsymbol{c}^{\mathsf{T}}\boldsymbol{C}\boldsymbol{w}}{\boldsymbol{c}^{\mathsf{T}}\boldsymbol{w}}, \tag{17}$$

Note that for $\boldsymbol{c}$ being a constant vector of ones, the L1-norm of the weight vector is maintained. However, $\boldsymbol{c}$ does not have to be constant. For example, for $\boldsymbol{c} = \boldsymbol{w}$ the L2-norm is maintained. Also, note that one can analogously derive effective plasticity rules when weights are constrained via subtractive normalization with the ansatz $\dot{\boldsymbol{w}} = \boldsymbol{C}\boldsymbol{w} - \zeta\boldsymbol{k}$, where $\boldsymbol{k}$ is a vector of ones (2).

[1] We indicate an equality or condition that we want to be fulfilled with an exclamation point over the equal sign.

1.2.1 Fixed points

From Eq. 16 it is clear that multiples of eigenvectors $\boldsymbol{v}$ of $\boldsymbol{C}$ are fixed points, for which $\dot{\boldsymbol{w}}^* = 0$. Explicitly, for a scalar constant a and $\boldsymbol{w}^* = a\boldsymbol{v}$ one gets:

$$\dot{\boldsymbol{w}}^* = a\boldsymbol{C}\boldsymbol{v} - \frac{\boldsymbol{c}^\mathsf{T}\boldsymbol{C}\boldsymbol{v}}{\boldsymbol{c}^\mathsf{T}\boldsymbol{v}}a\boldsymbol{v} = a\lambda\boldsymbol{v} - \frac{\boldsymbol{c}^\mathsf{T}\lambda\boldsymbol{v}}{\boldsymbol{c}^\mathsf{T}\boldsymbol{v}}a\boldsymbol{v} = 0. \quad [18]$$

Note that this is independent of the choice of the constraint vector $\boldsymbol{c}$. We next consider the stability of these eigenvector fixed points.

1.2.2 Stability analysis

In the previous sections, we showed how multiplicative normalization constrains the norm of the weight vector and therefore prevents the otherwise unlimited growth of Hebbian plasticity. However, even when the total synaptic weight is constrained, synaptic weights might still be unstable and never settle into a fixed point, e.g., experiencing oscillatory dynamics and unstable fixed points. Following Miller and MacKay (2), we will now explore under what conditions fixed points are stable.

Formally, a fixed point in a linear system is stable when the largest eigenvalue of the Jacobian is negative, or marginally stable when it is equal to zero (3). The weight dynamics around a fixed point $\boldsymbol{w}^*$ can be approximated with its Taylor expansion:

$$\dot{\boldsymbol{w}} \approx \dot{\boldsymbol{w}}^* + \sum_i \left.\frac{\mathrm{d}\dot{\boldsymbol{w}}}{\mathrm{d}w_i}\right|_* (w_i - w_i^*), \quad [19]$$

$$= \left.\frac{\mathrm{d}\dot{\boldsymbol{w}}}{\mathrm{d}\boldsymbol{w}}\right|_* (\boldsymbol{w} - \boldsymbol{w}^*), \quad [20]$$

$$= \boldsymbol{J}^*(\boldsymbol{w} - \boldsymbol{w}^*), \quad [21]$$

where $\dot{\boldsymbol{w}}^*$ is zero, by definition, and $\boldsymbol{J}^*$ is the Jacobian evaluated at the fixed point. The Jacobian is defined as

$$\boldsymbol{J}^* \equiv \begin{pmatrix} \left.\frac{\mathrm{d}\dot{w}_1}{\mathrm{d}w_1}\right|_* & \cdots & \left.\frac{\mathrm{d}\dot{w}_1}{\mathrm{d}w_N}\right|_* \\ \vdots & & \vdots \\ \left.\frac{\mathrm{d}\dot{w}_N}{\mathrm{d}w_1}\right|_* & \cdots & \left.\frac{\mathrm{d}\dot{w}_N}{\mathrm{d}w_N}\right|_* \end{pmatrix} \equiv \left.\frac{\mathrm{d}\dot{\boldsymbol{w}}}{\mathrm{d}\boldsymbol{w}}\right|_*. \quad [22]$$

A fixed point is stable if small perturbations away from the fixed point, $\Delta\boldsymbol{w} = \boldsymbol{w} - \boldsymbol{w}^*$, decay to zero, i.e.,

$$\frac{\mathrm{d}}{\mathrm{d}t}\Delta\boldsymbol{w} = \dot{\boldsymbol{w}} - \dot{\boldsymbol{w}}^* = \dot{\boldsymbol{w}} \approx \boldsymbol{J}^*\Delta\boldsymbol{w}, \quad [23]$$

where we approximated $\dot{\boldsymbol{w}}$ with its Taylor expansion (Eq. 19), since the perturbation is small, i.e., $\boldsymbol{w}$ is close to the fixed point. The result is a linear differential equation that one can solve as

$$\Delta\boldsymbol{w}(t) = \exp\big(\boldsymbol{J}^* t\big)\Delta\boldsymbol{w}(t_0), \quad [24]$$

where all vector components decay to zero if all eigenvalues of $\boldsymbol{J}^*$ are negative[1,2]. As we will see later, it is useful to rewrite the weight dynamics (Eq. 16) as

$$\dot{\boldsymbol{w}} = \boldsymbol{C}\boldsymbol{w} - \boldsymbol{w}\gamma, \quad [25]$$

$$= \boldsymbol{C}\boldsymbol{w} - \frac{\boldsymbol{w}\boldsymbol{c}^\mathsf{T}\boldsymbol{C}\boldsymbol{w}}{\boldsymbol{c}^\mathsf{T}\boldsymbol{w}}, \quad [26]$$

$$= \left[\mathbb{1} - \frac{\boldsymbol{w}\boldsymbol{c}^\mathsf{T}}{\boldsymbol{c}^\mathsf{T}\boldsymbol{w}}\right]\boldsymbol{C}\boldsymbol{w}. \quad [27]$$

[1]This can be seen by formulating the system in the eigenbasis of $\boldsymbol{J}^*$. Then, the matrix exponential becomes: $\boldsymbol{V}^{-1}\exp(\boldsymbol{J}^* t)\boldsymbol{V} = \exp(\Lambda_{\boldsymbol{J}} t)$, where $\boldsymbol{V}$ holds eigenvectors and $\Lambda_{\boldsymbol{J}}$ is a diagonal matrix that holds the eigenvalues of $\boldsymbol{J}^*$.

[2]In general, the real part of the eigenvalues of the Jacobian have to be negative for a fixed point to be stable. However, since C is a covariance matrix, it is positive definite with positive, real eigenvalues. We will see (Eq. 32) that from this it follows that the eigenvalues of the Jacobian are also real.

It follows[1]:

$$\left.\frac{\mathrm{d}\dot{\boldsymbol{w}}}{\mathrm{d}\boldsymbol{w}}\right|_{*} = \left[\mathbb{1} - \frac{\boldsymbol{v}^{*}\boldsymbol{c}^{\mathsf{T}}}{\boldsymbol{c}^{\mathsf{T}}\boldsymbol{v}^{*}}\right]\boldsymbol{C} + \left[-\frac{\mathbb{1}\boldsymbol{c}^{\mathsf{T}}}{\boldsymbol{c}^{\mathsf{T}}\boldsymbol{v}^{*}} + \frac{\boldsymbol{v}^{*}\boldsymbol{c}^{\mathsf{T}}\boldsymbol{c}^{\mathsf{T}}}{(\boldsymbol{c}^{\mathsf{T}}\boldsymbol{v}^{*})^{2}}\right]\boldsymbol{C}\boldsymbol{v}^{*}, \tag{28}$$

$$= \left[\mathbb{1} - \frac{\boldsymbol{v}^{*}\boldsymbol{c}^{\mathsf{T}}}{\boldsymbol{c}^{\mathsf{T}}\boldsymbol{v}^{*}}\right][\boldsymbol{C} - \lambda^{*}\mathbb{1}], \tag{29}$$

where $\boldsymbol{w}\big|_{*} = \boldsymbol{w}^{*} = a\boldsymbol{v}^{*}$ is the fixed point with $\boldsymbol{v}^{*}$ being an eigenvector of $\boldsymbol{C}$. The scalar a is the length of the fixed point weight vector $\boldsymbol{w}^{*}$ (which cancels) and λ^{*} is the eigenvalue to $\boldsymbol{v}^{*}$. To find the eigenvalues of the Jacobian, $\lambda_{\boldsymbol{J}}$, we diagonalize $\boldsymbol{J}$ by switching to the eigenbasis of $\boldsymbol{C}$. When $\boldsymbol{V}$ is the matrix that holds the eigenvectors of $\boldsymbol{C}$ as columns one gets

$$\boldsymbol{V}^{\mathsf{T}}\left.\frac{\mathrm{d}\dot{\boldsymbol{w}}}{\mathrm{d}\boldsymbol{w}}\right|_{*}\boldsymbol{V} = \left[\mathbb{1} - \boldsymbol{V}^{\mathsf{T}}\frac{\boldsymbol{v}^{*}\boldsymbol{c}^{\mathsf{T}}}{\boldsymbol{c}^{\mathsf{T}}\boldsymbol{v}^{*}}\boldsymbol{V}\right]\left[\boldsymbol{V}^{\mathsf{T}}\boldsymbol{C}\boldsymbol{V} - \lambda^{*}\mathbb{1}\right], \tag{30}$$

$$= \left[\mathbb{1} - \boldsymbol{e}^{*}\frac{\boldsymbol{c}^{\mathsf{T}}\boldsymbol{V}}{\boldsymbol{c}^{\mathsf{T}}\boldsymbol{v}^{*}}\right][\Lambda - \lambda^{*}\mathbb{1}], \tag{31}$$

where Λ is a diagonal matrix that holds the eigenvalues of $\boldsymbol{C}$. Without loss of generality, we can assume that the first column of $\boldsymbol{V}$ is equal to $\boldsymbol{v}^{*}$. Then $\boldsymbol{e}^{*} = \boldsymbol{V}^{\mathsf{T}}\boldsymbol{v}^{*}$ is a column vector of zeros, except for the first entry, which is equal to one. Then, the first bracket becomes an upper triangular matrix with ones on the diagonal, except for the first diagonal entry, which is zero. From this, it follows[2] that the eigenvalues of the Jacobian are

$$\boxed{\lambda_{\boldsymbol{J}} = \lambda - \lambda^{*}.} \tag{32}$$

If λ^{*} is the largest eigenvalue, i.e., $\boldsymbol{w}^{*}$ is a multiple of the principal eigenvector of $\boldsymbol{C}$, then all $\lambda_{\boldsymbol{J}}$ are negative or zero, and the fixed point is marginally stable. If there exists a $\lambda > \lambda^{*}$, the corresponding $\lambda_{\boldsymbol{J}}$ is positive and the fixed point is unstable. Therefore, the eigenvector corresponding to the principal eigenvalue is the only (marginally) stable fixed point. In summary, linear Hebbian learning combined with multiplicative normalization becomes selective for the principal eigenvector of the input covariance matrix and thus performs principal component analysis (PCA). Next, we consider what happens when a neuron also receives inhibitory input.

1.2.3 Classic Inhibitory plasticity prevents stimulus selectivity

Previous work suggested a homeostatic inhibitory synaptic plasticity rule (*4*) that enforced a post-synaptic target firing rate r_0:

$$\dot{\mathbf{w}}_I \propto \mathbf{y}_I\,(r - r_0)\,. \tag{33}$$

However, when combined with excitatory plasticity, this classic rule prevents the development of stimulus selectivity (cf. Fig. 1*A*, *E* & *F*). For completeness, we briefly recapitulate this result, presented in Clopath *et al.*(*5*): We consider a simplified circuit of a single post-synaptic neuron with firing rate r that receives lateral input from N_I inhibitory neurons, while all neurons receive feedforward input from a population of N_E excitatory neurons[3] (cf. Fig. S1*C*). Then, $\boldsymbol{y}_{\boldsymbol{E}}$ and $\boldsymbol{y}_{\boldsymbol{I}}$ are vectors that hold the firing rates of the excitatory and inhibitory populations. We now explore the self-organization of excitatory and inhibitory synaptic weights, $\boldsymbol{w}_E$ and $\boldsymbol{w}_I$, that project onto the single post-synaptic neuron, while input synapses $\boldsymbol{Q}$ that project onto inhibitory neurons remain fixed. In Clopath *et al.* (*5*), the authors find that classic inhibitory plasticity is required to act faster than excitatory plasticity to enable stable weight dynamics (*5*). For much faster inhibitory plasticity, the dynamics of excitatory and inhibitory weights decouples, and fixed points of the inhibitory weights $\mathbf{w}_I^{*}$ can be considered separately from the fixed points of excitatory weights. When excitatory and inhibitory inputs are equally stimulus selective, the fast dynamics of inhibitory weights ensures that the target firing rate is consistently met, $r^{*} \approx r_0$, and plasticity of excitatory synapses only depend on pre-synaptic terms and constants[4]:

$$\langle\dot{\mathbf{w}}_I^{*}\rangle = 0 \quad\Rightarrow\quad \langle\dot{\mathbf{w}}_E\rangle \propto \langle\mathbf{y}_E\rangle r_0 - \text{normalization.} \tag{34}$$

[1]To make sense of the vector notation, it helps to first consider the b'th column of $\frac{\mathrm{d}\dot{\boldsymbol{w}}}{\mathrm{d}\boldsymbol{w}}$ which is equal to $\frac{\mathrm{d}\dot{\boldsymbol{w}}}{\mathrm{d}w_b}$, where w_b is the b'th vector component of $\boldsymbol{w}$.

[2]Because the eigenvalues of a product of two triangular matrices is equal to the product of their eigenvalues.

[3]Note that N_I does not necessarily equal N_E.

[4]More precisely, we assume that excitatory and inhibitory inputs are similarly tuned, i.e., $\boldsymbol{y}_E = \boldsymbol{Q}^{-1}\boldsymbol{y}_I$. From $\langle\dot{\boldsymbol{w}}_I\rangle = 0$ we get $\langle\boldsymbol{y}_I r\rangle = \langle\boldsymbol{y}_I\rangle r_0$, which after multiplying by $\boldsymbol{Q}^{-1}$ becomes $\langle\boldsymbol{Q}^{-1}\boldsymbol{y}_I r\rangle = \langle\boldsymbol{Q}^{-1}\boldsymbol{y}_I\rangle r_0$. Then, for excitatory plasticity one gets $\langle\dot{\boldsymbol{w}}_E\rangle = \langle\boldsymbol{Q}^{-1}\boldsymbol{y}_I r\rangle$ − normalization = $\langle\boldsymbol{y}_E\rangle r_0$ − normalization, as stated in Eq. 34.

When all pre-synaptic neurons have similar average firing rates, $\langle y_E \rangle_i \approx y_0$, and weights change on a slower timescale than activities, as is the case biologically, the average excitatory synaptic weight change becomes

$$\langle \dot{\mathbf{w}}_E \rangle \propto \mathbf{c} y_0 r_0 - \text{normalization}, \tag{35}$$

where $\mathbf{c}$ is a vector of ones. The average synaptic weight change is identical across synapses, which prevents the development of stimulus selectivity (Fig. 1*E* & *F*). Therefore, classic inhibitory plasticity that enforces a target firing rate cannot explain the joint development of stimulus selectivity and inhibitory balance. Instead, we propose that, as excitatory weights, also inhibitory weights are constrained via a competitive process that normalizes the total inhibitory input that a neuron receives.

2 Synapse-type-specific normalization balances E-I receptive fields

Different from the normalization of excitatory weights, the normalization of inhibitory weights is not motivated by the requirement for stability. Inhibitory synaptic plasticity that depends on neural activity is self-limiting, since increasing inhibitory weights eventually prevent the neuron from firing, and thus prevent further plasticity. Instead, we motivate the normalization of inhibitory synaptic weights by the competition for a limited amount of synaptic building blocks that may also drive excitatory normalization (see Main text for details). In the following, we generalize the approach outlined in the previous Sections for excitatory weight normalization to the case of simultaneous excitatory and inhibitory normalization. We consider the same circuit architecture as in Section 1.2.3 (cf. Fig. S1*C*) with rate dynamics

$$\tau_r \dot{r} = -r + \boldsymbol{y}_E{}^{\mathsf{T}} \boldsymbol{w}_E - \boldsymbol{y}_I{}^{\mathsf{T}} \boldsymbol{w}_I{}^{\mathsf{T}} = -r + \bar{\boldsymbol{y}}^{\mathsf{T}} \begin{pmatrix} \mathbb{1} & \mathbf{0} \\ \mathbf{0} & -\mathbb{1} \end{pmatrix} \bar{\boldsymbol{w}}, \tag{36}$$

$$\bar{\boldsymbol{w}} = \begin{pmatrix} \boldsymbol{w}_E \\ \boldsymbol{w}_I \end{pmatrix}, \quad \bar{\boldsymbol{y}} = \begin{pmatrix} \boldsymbol{y}_E \\ \boldsymbol{y}_I \end{pmatrix}, \quad \boldsymbol{y}_I = \boldsymbol{Q}\boldsymbol{y}_E, \tag{37}$$

where $\mathbb{1}$ is the unit matrix with appropriate dimension, $\mathbf{0}$ are matrices of zeros and appropriate dimensionality, and we defined the modified weight and input vectors, $\bar{\boldsymbol{w}}$ and $\bar{\boldsymbol{y}}$. Similar to before, we assume fast activity dynamics, $\tau_r \ll 1$, and write the Hebbian part of the time-averaged weight dynamics as

$$\bar{\boldsymbol{\tau}} \left\langle \dot{\bar{\boldsymbol{w}}} \right\rangle = \langle \bar{\boldsymbol{y}} r \rangle = \left\langle \bar{\boldsymbol{y}} \bar{\boldsymbol{y}}^{\mathsf{T}} \right\rangle \begin{pmatrix} \mathbb{1} & \mathbf{0} \\ \mathbf{0} & -\mathbb{1} \end{pmatrix} \bar{\boldsymbol{w}}, \tag{38}$$

$$= \left\langle \begin{pmatrix} \boldsymbol{y}_E \boldsymbol{y}_E{}^{\mathsf{T}} & -\boldsymbol{y}_E \boldsymbol{y}_I{}^{\mathsf{T}} \\ \boldsymbol{y}_I \boldsymbol{y}_E{}^{\mathsf{T}} & -\boldsymbol{y}_I \boldsymbol{y}_I{}^{\mathsf{T}} \end{pmatrix} \right\rangle \bar{\boldsymbol{w}} \equiv \bar{\boldsymbol{C}} \bar{\boldsymbol{w}}, \tag{39}$$

where we defined the modified covariance matrix $\bar{\boldsymbol{C}}$. In general, we assume that all synapses of one type, excitatory or inhibitory, change equally fast (cf. Table 1). Then, the matrix $\bar{\boldsymbol{\tau}}$ holds the timescales of excitatory plasticity, $\boldsymbol{\tau}_E = \mathbb{1}\tau_E$, and inhibitory plasticity, $\boldsymbol{\tau}_I = \mathbb{1}\tau_I$, as matrices on the diagonal, and is zero otherwise. In the following, we drop the bracket notation $\langle \cdot \rangle$ for better readability. As in the case of only excitatory input, we can implement multiplicative normalization by additional constraint terms. Now also for inhibitory weights (cf. Eq. 16):

$$\boxed{\bar{\boldsymbol{\tau}} \dot{\bar{\boldsymbol{w}}} = \bar{\boldsymbol{C}} \bar{\boldsymbol{w}} - \gamma \bar{\boldsymbol{w}}_E - \rho \bar{\boldsymbol{w}}_I}\,, \tag{40}$$

$$\bar{\boldsymbol{w}}_E = \begin{pmatrix} \boldsymbol{w}_E \\ \mathbf{0} \end{pmatrix}, \quad \bar{\boldsymbol{w}}_I = \begin{pmatrix} \mathbf{0} \\ \boldsymbol{w}_I \end{pmatrix}, \tag{41}$$

where $\mathbf{0}$ indicates vectors of zeros of appropriate dimension (N_I and N_E) that we do not specify for better readability.

The constraint factors γ and ρ follow from the requirement that the weight vector does not grow along the direction of the constraint vectors $\bar{\boldsymbol{c}}_E$ and $\bar{\boldsymbol{c}}_I$. Here we choose them such that the sums over the excitatory and inhibitory

weights remain constant, i.e., the L1-norm of the excitatory and inhibitory part of the weight vector is maintained[1].

$$\bar{\boldsymbol{c}}_E{}^\mathsf{T}\dot{\bar{\boldsymbol{w}}} \stackrel{!}{=} 0, \quad \bar{\boldsymbol{c}}_I{}^\mathsf{T}\dot{\bar{\boldsymbol{w}}} \stackrel{!}{=} 0, \tag{42}$$

$$\bar{\boldsymbol{c}}_E{}^\mathsf{T} \equiv \Big(1, \dots, 1, 0, \dots, 0\Big), \quad \bar{\boldsymbol{c}}_I{}^\mathsf{T} \equiv \Big(0, \dots, 0, 1, \dots, 1\Big), \tag{43}$$

where the number of non-zero entries in $\bar{\boldsymbol{c}}_E$ and $\bar{\boldsymbol{c}}_I$ is equal to the number of excitatory N_E and inhibitory neurons N_I, respectively. Based on these requirements we derive expressions for the scalar constraint factors γ and ρ:

$$\Rightarrow \quad \gamma = \frac{\bar{\boldsymbol{c}}_E{}^\mathsf{T}\bar{\boldsymbol{C}}\bar{\boldsymbol{w}}}{\bar{\boldsymbol{c}}_E{}^\mathsf{T}\bar{\boldsymbol{w}}_E}, \quad \rho = \frac{\bar{\boldsymbol{c}}_I{}^\mathsf{T}\bar{\boldsymbol{C}}\bar{\boldsymbol{w}}}{\bar{\boldsymbol{c}}_I{}^\mathsf{T}\bar{\boldsymbol{w}}_I}. \tag{44}$$

Finally, we can write the weight dynamics as

$$\Rightarrow \quad \boxed{\bar{\boldsymbol{\tau}}\dot{\bar{\boldsymbol{w}}} = \left[\mathbb{1} - \frac{\bar{\boldsymbol{w}}_E\bar{\boldsymbol{c}}_E{}^\mathsf{T}}{\bar{\boldsymbol{c}}_E{}^\mathsf{T}\bar{\boldsymbol{w}}_E} - \frac{\bar{\boldsymbol{w}}_I\bar{\boldsymbol{c}}_I{}^\mathsf{T}}{\bar{\boldsymbol{c}}_I{}^\mathsf{T}\bar{\boldsymbol{w}}_I}\right]\bar{\boldsymbol{C}}\bar{\boldsymbol{w}}}. \tag{45}$$

2.1 Fixed points

For the fixed points we have to find weight vectors $\bar{\boldsymbol{w}}^*$ for which the time derivative $\dot{\bar{\boldsymbol{w}}}^*$ is equal to zero:

$$\bar{\boldsymbol{\tau}}\dot{\bar{\boldsymbol{w}}}^* = \bar{\boldsymbol{C}}\bar{\boldsymbol{w}}^* - \gamma\bar{\boldsymbol{w}}_E^* - \rho\bar{\boldsymbol{w}}_I^* \tag{46}$$

$$= \bar{\boldsymbol{C}}\bar{\boldsymbol{w}}^* - \frac{\bar{\boldsymbol{c}}_E{}^\mathsf{T}\bar{\boldsymbol{C}}\bar{\boldsymbol{w}}^*}{\bar{\boldsymbol{c}}_E{}^\mathsf{T}\bar{\boldsymbol{w}}_E^*}\bar{\boldsymbol{w}}_E^* - \frac{\bar{\boldsymbol{c}}_I{}^\mathsf{T}\bar{\boldsymbol{C}}\bar{\boldsymbol{w}}^*}{\bar{\boldsymbol{c}}_I{}^\mathsf{T}\bar{\boldsymbol{w}}_I^*}\bar{\boldsymbol{w}}_I^* \stackrel{!}{=} \boldsymbol{0}, \tag{47}$$

which is equivalent to

$$\boxed{\bar{\boldsymbol{C}}\bar{\boldsymbol{w}}^* \stackrel{!}{=} \lambda_E\bar{\boldsymbol{w}}_E^* + \lambda_I\bar{\boldsymbol{w}}_I^*}, \tag{48}$$

for λ_E and λ_I being arbitrary scalar.

2.1.1 Eigenvectors of the modified covariance matrix are fixed points

It is straightforward to check that multiples of eigenvectors $\bar{\boldsymbol{v}}$ of the modified covariance matrix $\bar{\boldsymbol{C}}$ with eigenvalue $\bar{\lambda}$ are fixed points:

$$\bar{\boldsymbol{C}}\bar{\boldsymbol{v}} = \bar{\lambda}\bar{\boldsymbol{v}}_E + \bar{\lambda}\bar{\boldsymbol{v}}_I = \lambda_E\bar{\boldsymbol{v}}_E + \lambda_I\bar{\boldsymbol{v}}_I \quad \Rightarrow \quad \lambda_E = \lambda_I = \bar{\lambda}. \tag{49}$$

In the following, we will refer to eigenvectors of the modified covariance matrix as fixed point eigenvectors, and to eigenvectors of the feedforward excitatory covariance matrix $\boldsymbol{C}$ as feedforward eigenvectors. Next, we will try to specify the eigenvectors of $\bar{\boldsymbol{C}}$. In general, eigenvectors of $\bar{\boldsymbol{C}}$ depend non-trivially on the tuning of the laterally projecting population (cf. Sec. 3, Eq. 121). However, the problem simplifies when the laterally projecting inhibitory neurons are tuned to multiples of eigenvectors of the excitatory population's covariance matrix. This is what one would expect when the post-synaptic excitatory neuron r and the inhibitory population $\boldsymbol{y}_E$ both receive excitatory input from the same external brain region $\boldsymbol{y}_E$ and synapses from the external population onto inhibitory neurons are plastic according to a Hebbian rule with multiplicative normalization (cf. Fig. S1C). Although we showed in Section 1.2.2 that without recurrent interactions only the principal eigenvector is a stable fixed point, we will find that with suitable recurrent interactions any feedforward eigenvector can be stable (cf. Sec. 3 & 5.2.3). Formally we set

$$\boldsymbol{y}_I = \boldsymbol{Q}\boldsymbol{y}_E = \boldsymbol{A}^\mathsf{T}\boldsymbol{V}^\mathsf{T}\boldsymbol{y}_E, \tag{50}$$

where each row of $\boldsymbol{Q} = \boldsymbol{A}^\mathsf{T}\boldsymbol{V}^\mathsf{T}$ is the feedforward weight vector of an inhibitory neuron which is equal to a positive multiple, a, of an eigenvector $\boldsymbol{v}$ of the excitatory covariance matrix $\boldsymbol{C} = \langle \boldsymbol{y}_E\boldsymbol{y}_E{}^\mathsf{T}\rangle$. Then $\boldsymbol{V}$ holds all eigenvectors as columns, and $\boldsymbol{A}$ is a matrix where each multiple is the only non-zero element per column, such that $\boldsymbol{A}\boldsymbol{A}^\mathsf{T}$ is a diagonal matrix. We will now show that in this scenario multiples of the excitatory and inhibitory part of the eigenvectors of the modified covariance matrix $\bar{\boldsymbol{C}}$ are fixed points. As a first step, we explicitly calculate the eigenvectors.

[1]The choice of the L1-norm is motivated by the synaptic competition for a fixed amount of resources, where, in the simplest case, each unit of resource linearly increases synaptic strengths. Higher-order L-norms do not affect the learning of feedforward receptive fields. However, in recurrent networks, they can lead to instabilities (cf. Sec. 4.3).

2.1.2 Eigenvectors and eigenvalues of the modified covariance matrix

In the previous section, we have seen that eigenvectors $\bar{\boldsymbol{v}}$ of the modified covariance matrix $\bar{\boldsymbol{C}}$ are fixed points. In this section, we will find an explicit expression for these eigenvectors when inhibitory neurons are tuned to feedforward eigenvectors, i.e., inhibitory neurons are tuned to eigenvectors $\boldsymbol{v}$ of the excitatory covariance matrix $\boldsymbol{C}$. Making use of Eq. 50 the modified covariance matrix becomes

$$\bar{\boldsymbol{C}} = \left\langle \begin{pmatrix} \boldsymbol{y}_E\boldsymbol{y}_E{}^\top & -\boldsymbol{y}_E\boldsymbol{y}_I{}^\top \\ \boldsymbol{y}_I\boldsymbol{y}_E{}^\top & -\boldsymbol{y}_I\boldsymbol{y}_I{}^\top \end{pmatrix} \right\rangle = \begin{pmatrix} \boldsymbol{C} & -\boldsymbol{CVA} \\ \boldsymbol{A}^\top\boldsymbol{V}^\top\boldsymbol{C} & -\boldsymbol{A}^\top\boldsymbol{V}^\top\boldsymbol{CVA} \end{pmatrix} = \begin{pmatrix} \boldsymbol{C} & -\boldsymbol{V}\Lambda\boldsymbol{A} \\ \boldsymbol{A}^\top\Lambda\boldsymbol{V}^\top & -\boldsymbol{A}^\top\Lambda\boldsymbol{A} \end{pmatrix}. \quad [51]$$

Then, a full set[1] of linearly independent eigenvectors $\bar{\boldsymbol{V}}$ and their inverse $\bar{\boldsymbol{V}}^{-1}$ is given as[2].

$$\bar{\boldsymbol{V}} = \begin{pmatrix} \boldsymbol{V} & \boldsymbol{VA} \\ \boldsymbol{A}^\top & \mathbb{1} \end{pmatrix}, \quad \bar{\boldsymbol{V}}^{-1} = \begin{pmatrix} (\mathbb{1}-\boldsymbol{AA}^\top)^{-1} & \boldsymbol{0} \\ \boldsymbol{0} & (\mathbb{1}-\boldsymbol{A}^\top\boldsymbol{A})^{-1} \end{pmatrix} \begin{pmatrix} \boldsymbol{V}^\top & -\boldsymbol{A} \\ -\boldsymbol{A}^\top\boldsymbol{V}^\top & \mathbb{1} \end{pmatrix}, \quad [52]$$

where each column of $\bar{\boldsymbol{V}}$ is an non-normalized eigenvector. The eigenvalue spectrum is

$$\bar{\boldsymbol{C}}\bar{\boldsymbol{V}} \stackrel{!}{=} \bar{\boldsymbol{V}}\bar{\Lambda} \quad \Rightarrow \bar{\Lambda} = \begin{pmatrix} \Lambda(\mathbb{1}-\boldsymbol{AA}^\top) & \boldsymbol{0} \\ \boldsymbol{0} & \boldsymbol{0} \end{pmatrix}. \quad [53]$$

Similar to before, we call eigenvectors of the modified covariance matrix with positive eigenvalue *attractive*. Different from the case of only excitatory feedforward input, eigenvalues of the modified covariance matrix can also be negative. In this case, we call the corresponding eigenvector *repulsive* (cf. Sec. 1.1).

For eigenvectors in the right matrix column of $\bar{\boldsymbol{V}}$ in Eq. 52, the excitatory and inhibitory components of the membrane potential exactly cancel, post-synaptic firing rates are zero, and no plasticity is induced: For multiple post-synaptic neurons with firing rates $\boldsymbol{r}$, where each neuron is tuned to one of these eigenvectors, one gets

$$\boldsymbol{r} = \bar{\boldsymbol{y}}^\top \begin{pmatrix} \mathbb{1} & \boldsymbol{0} \\ \boldsymbol{0} & -\mathbb{1} \end{pmatrix} \bar{\boldsymbol{V}}^\circ, \quad \bar{\boldsymbol{V}}^\circ = \begin{pmatrix} \boldsymbol{VA} \\ \mathbb{1} \end{pmatrix}, \quad \bar{\boldsymbol{y}} = \begin{pmatrix} \boldsymbol{y}_E \\ \boldsymbol{y}_I \end{pmatrix}, \quad \boldsymbol{y}_I = \boldsymbol{A}^\top\boldsymbol{V}^\top\boldsymbol{y}_E, \quad [54]$$

$$\Rightarrow \quad \boldsymbol{r} = \left(\boldsymbol{y}_E{}^\top, -\boldsymbol{y}_E{}^\top\boldsymbol{VA}\right)\begin{pmatrix} \boldsymbol{VA} \\ \mathbb{1} \end{pmatrix} = \boldsymbol{0}. \quad [55]$$

Since these eigenvectors result in post-synaptic firing rates of zero, and they define the null space of the $\bar{\boldsymbol{C}}$ matrix (Eq. 53), we call them 'null eigenvectors' or 'null fixed points', and all eigenvectors that are not null eigenvectors 'regular' eigenvectors or fixed points. Note that for each additional inhibitory neuron that is tuned to a feedforward eigenvector, there is an additional null eigenvector, since inhibitory synaptic weights can now shift between the original, and the additional inhibitory neuron to cancel post-synaptic firing. Overall, there are always N_I null eigenvectors and N_E regular eigenvectors[3]. Note that $\boldsymbol{A}$ is a matrix with exactly one non-zero element per column (cf. Eq. 50f.), and we can see from Eq. 52 that the excitatory part of each null eigenvector is proportional to the excitatory part of one regular eigenvector. In the following, when we speak of regular eigenvectors and corresponding null eigenvectors, we mean eigenvectors with proportional excitatory components.

We have already shown in Section 2.1.1 that eigenvectors of $\bar{\boldsymbol{C}}$ are fixed points. Each eigenvector specifies an exact ratio between the excitatory and inhibitory weight norm. Since our learning rule separately maintains the total excitatory and inhibitory synaptic weights, reaching any of these fixed points would require detailed fine-tuning at the point of initialization. In the next section, we show a more general set of fixed points that does not require any fine tuning of weight norms.

2.1.3 Non-eigenvector fixed points

In this section, we show that there exist fixed points that are not eigenvectors of the modified covariance matrix. In particular, arbitrary multiples of the excitatory and inhibitory parts of regular eigenvectors, i.e., of eigenvectors that

[1] Note that $\boldsymbol{A}$ and $\boldsymbol{VA}$ are of dimension $N_E \times N_I$, and $\bar{\boldsymbol{V}}$ is of dimension $(N_E+N_I)\times(N_E+N_I)$.

[2] To show that $\bar{\boldsymbol{V}}^{-1}$ is the inverse of $\bar{\boldsymbol{V}}$ it is useful to define the Moore-Penrose inverse $\boldsymbol{A}^{-1} = \boldsymbol{A}^\top(\boldsymbol{AA}^\top)^{-1}$ and note that $\boldsymbol{A}^\top(\mathbb{1}-\boldsymbol{AA}^\top)^{-1} = (\mathbb{1}-\boldsymbol{A}^\top\boldsymbol{A})^{-1}\boldsymbol{A}^\top$.

[3] Similarly, each additional laterally projecting excitatory neuron adds another null eigenvector. In that case, the lateral excitatory weight component and the feedforward excitatory weight component have opposite signs such that they cancel each other (cf. Sec. 5.1).

result in non-zero post-synaptic activity, are fixed points. We make the ansatz that the matrix $\overline{\boldsymbol{W}}^*$ holds fixed points as columns and has the shape

$$\overline{\boldsymbol{W}}^* = \begin{pmatrix} \boldsymbol{V}\boldsymbol{K}_E \\ \boldsymbol{A}^\top \boldsymbol{K}_I \end{pmatrix}, \quad [56]$$

where $\boldsymbol{K}_E$ and $\boldsymbol{K}_I$ and are diagonal scaling matrices of arbitrary constants. The fixed point condition that follows from Eq. 48 is

$$\overline{\boldsymbol{C}}\overline{\boldsymbol{W}}^* \overset{!}{=} \begin{pmatrix} \boldsymbol{W}_E^* \Lambda_E \\ \boldsymbol{W}_I^* \Lambda_I \end{pmatrix}. \quad [57]$$

We now show that for any $\boldsymbol{K}_E$, $\boldsymbol{K}_I$ we can find diagonal matrices Λ_E, Λ_I that fulfil this condition[1]. We write explicitly

$$\Rightarrow \quad \overline{\boldsymbol{C}}\overline{\boldsymbol{W}}^* = \begin{pmatrix} \boldsymbol{C} & -\boldsymbol{V}\Lambda\boldsymbol{A} \\ \boldsymbol{A}^\top \Lambda \boldsymbol{V}^\top & -\boldsymbol{A}^\top \Lambda \boldsymbol{A} \end{pmatrix} \begin{pmatrix} \boldsymbol{V}\boldsymbol{K}_E \\ \boldsymbol{A}^\top \boldsymbol{K}_I \end{pmatrix} \overset{!}{=} \begin{pmatrix} \boldsymbol{V}\boldsymbol{K}_E \Lambda_E \\ \boldsymbol{A}^\top \boldsymbol{K}_I \Lambda_I \end{pmatrix} \quad [58]$$

$$\boldsymbol{C}\boldsymbol{V}\boldsymbol{K}_E - \boldsymbol{V}\Lambda\boldsymbol{A}\boldsymbol{A}^\top \boldsymbol{K}_I \overset{!}{=} \boldsymbol{V}\boldsymbol{K}_E\Lambda_E, \quad [59]$$

$$\boldsymbol{A}^\top \Lambda \boldsymbol{V}^\top \boldsymbol{V}\boldsymbol{K}_E - \boldsymbol{A}^\top \Lambda \boldsymbol{A}\boldsymbol{A}^\top \boldsymbol{K}_I \overset{!}{=} \boldsymbol{A}^\top \boldsymbol{K}_I \Lambda_I. \quad [60]$$

$$\boldsymbol{V}\boldsymbol{K}_E\Lambda - \boldsymbol{V}\boldsymbol{K}_I\boldsymbol{A}\boldsymbol{A}^\top \Lambda \overset{!}{=} \boldsymbol{V}\boldsymbol{K}_E\Lambda_E, \quad [61]$$

$$\boldsymbol{A}^\top \boldsymbol{K}_E\Lambda - \boldsymbol{A}^\top \boldsymbol{K}_I\Lambda\boldsymbol{A}\boldsymbol{A}^\top \overset{!}{=} \boldsymbol{A}^\top \boldsymbol{K}_I\Lambda_I, \quad [62]$$

where we made use of the fact that independent of their subscript, the $\boldsymbol{K}$, Λ, and $\boldsymbol{A}\boldsymbol{A}^\top$ matrices are diagonal and commute. By comparing the left and right sides of the equations, we find

$$\Lambda_E = \Lambda \left(\mathbb{1} - \boldsymbol{K}_E^{-1}\boldsymbol{K}_I\boldsymbol{A}\boldsymbol{A}^\top \right), \quad [63]$$

$$\Lambda_I = \Lambda \left(\boldsymbol{K}_I^{-1}\boldsymbol{K}_E - \boldsymbol{A}\boldsymbol{A}^\top \right), \quad [64]$$

which are diagonal matrices, as required[2]. Before we consider the stability of these fixed points in Section 2.2, we first show that there is an additional set of fixed points.

2.1.4 General fixed points

Having covered various special cases of fixed points for the dynamics, we now consider the general problem. Recall that fixed points are defined to satisfy Equation 48:

$$\overline{\boldsymbol{C}}\overline{\boldsymbol{w}}^* = \lambda_E \overline{\boldsymbol{w}}_E^* + \lambda_I \overline{\boldsymbol{w}}_I^* \quad [65]$$

Expanding this using our expression for $\overline{\boldsymbol{C}}$ (Eq. 51), we can see that this is equivalent to:

$$\boldsymbol{V}\Lambda\boldsymbol{V}^\top \boldsymbol{w}_E^* - \boldsymbol{V}\Lambda\boldsymbol{A}\boldsymbol{w}_I^* = \lambda_E \boldsymbol{w}_E^*, \quad [66]$$

$$\boldsymbol{A}^\top \Lambda \boldsymbol{V}^\top \boldsymbol{w}_E^* - \boldsymbol{A}^\top \Lambda \boldsymbol{A}\boldsymbol{w}_I^* = \lambda_I \boldsymbol{w}_I^*, \quad [67]$$

and equivalently

$$\Lambda(\boldsymbol{V}^\top \boldsymbol{w}_E^* - \boldsymbol{A}\boldsymbol{w}_I^*) = \lambda_E \boldsymbol{V}^\top \boldsymbol{w}_E^*, \quad [68]$$

$$\boldsymbol{A}^\top \Lambda(\boldsymbol{V}^\top \boldsymbol{w}_E^* - \boldsymbol{A}\boldsymbol{w}_I^*) = \lambda_I \boldsymbol{w}_I^*. \quad [69]$$

Inserting the first into the second expression, we can conclude that

$$\lambda_E \boldsymbol{A}^\top \boldsymbol{V}^\top \boldsymbol{w}_E^* = \lambda_I \boldsymbol{w}_I^*. \quad [70]$$

[1] For $\boldsymbol{K}_E = \mathbb{1}$ and $\boldsymbol{K}_I = (\boldsymbol{A}\boldsymbol{A}^\top)^{-1}$ columns of $\boldsymbol{W}^*$ holds null eigenvectors that can be formed by a linear combination of null eigenvectors $\overline{\boldsymbol{V}}^\circ$ given in Eq. 54.

[2] In general, this is not the case for null eigenvectors. Following the same formalism for null eigenvectors $\boldsymbol{W}^{*\top} = (\boldsymbol{K}_E{}^\top \boldsymbol{A}^\top \boldsymbol{V}^\top, \boldsymbol{K}_I{}^\top)^\top$ one finds the condition that $\boldsymbol{A}^\top \Lambda \boldsymbol{A}$ must be diagonal. In general, this is not the case, e.g., when multiple inhibitory neurons are tuned to the same eigenvector, i.e., when multiple columns of $\boldsymbol{A}$ hold the same vector up to a constant factor.

If $\lambda_E = \lambda_I \neq 0$, then we know that $\bar{\boldsymbol{w}}^*$ is an eigenvector of the modified covariance matrix, as discussed in Section 2.1.1. In the case that $\lambda_E = \lambda_I = 0$, we have the null eigenvectors discussed in Section 2.1.2. We therefore now address the case that $\lambda_E \neq \lambda_I$.

We begin with the case $\lambda_I \neq 0$. Then we can insert Eq. 70 into Eq. 68 to arrive at

$$\Lambda\left(\mathbb{1} - \frac{\lambda_E}{\lambda_I}\boldsymbol{A}\boldsymbol{A}^\top\right)\boldsymbol{V}^\top \boldsymbol{w}_E^* = \lambda_E \boldsymbol{V}^\top \boldsymbol{w}_E^*, \quad [71]$$

which, together with Eq. 70, gives necessary and sufficient conditions for a fixed point. From Eq. 71, we conclude that $\boldsymbol{V}^\top \boldsymbol{w}_E^*$ is an eigenvector of the diagonal matrix $\Lambda\left(\mathbb{1} - \frac{\lambda_E}{\lambda_I}\boldsymbol{A}\boldsymbol{A}^\top\right)$ with eigenvalue λ_E. When $\boldsymbol{V}^\top \boldsymbol{w}_E^*$ is one-hot, then the vector $\bar{\boldsymbol{w}}^*$ consists of an arbitrary multiple of the excitatory and inhibitory parts of a regular eigenvector, as covered in Section 2.1.3.

We now turn our attention to the case where $\boldsymbol{V}^\top \boldsymbol{w}_E^*$ is not simply one-hot. We can now say that for each component j of $\boldsymbol{V}^\top \boldsymbol{w}_E^*$ which is non-zero, the following equation must hold:

$$\lambda_j\left(1 - \frac{\lambda_E}{\lambda_I}(\boldsymbol{A}\boldsymbol{A}^\top)_{jj}\right) = \lambda_E. \quad [72]$$

This is a linear system in the pair of variables λ_E and λ_E/λ_I. We work under the mild assumptions that the eigenvalues λ_j, the diagonal elements $(\boldsymbol{A}\boldsymbol{A}^\top)_{jj}$, and their product $\lambda_j(\boldsymbol{A}\boldsymbol{A}^\top)_{jj}$ are distinct for each j. These conditions will in general hold in the absence of fine tuning. In this case, λ_E and λ_I provide two degrees of freedom and there will only be solutions when $\boldsymbol{V}^\top \boldsymbol{w}_E^*$ is (at most) two-hot, having non-zero components, j and k. Such solutions satisfy:

$$\begin{pmatrix}\lambda_j \\ \lambda_k\end{pmatrix} = \begin{pmatrix}\lambda_j(\boldsymbol{A}\boldsymbol{A}^\top)_{jj} & 1 \\ \lambda_k(\boldsymbol{A}\boldsymbol{A}^\top)_{kk} & 1\end{pmatrix}\begin{pmatrix}\lambda_E/\lambda_I \\ \lambda_E\end{pmatrix}, \quad [73]$$

which we can solve to obtain the expressions:

$$\lambda_E = \lambda_j\lambda_k \frac{(\boldsymbol{A}\boldsymbol{A}^\top)_{jj} - (\boldsymbol{A}\boldsymbol{A}^\top)_{kk}}{\lambda_j(\boldsymbol{A}\boldsymbol{A}^\top)_{jj} - \lambda_k(\boldsymbol{A}\boldsymbol{A}^\top)_{kk}}, \quad \lambda_I = \lambda_j\lambda_k \frac{(\boldsymbol{A}\boldsymbol{A}^\top)_{jj} - (\boldsymbol{A}\boldsymbol{A}^\top)_{kk}}{\lambda_j - \lambda_k}. \quad [74]$$

The components of the two-hot solution are determined by the known initial values of $k_E = \boldsymbol{c}_E{}^\top \boldsymbol{w}_E$ and $k_I = \boldsymbol{c}_I{}^\top \boldsymbol{w}_I$, which are kept constant throughout training [1]. Although two-hot fixed points do not require fine tuning of excitatory and inhibitory weight norms, we did not observe them in any of our numerical simulations and therefore assume they are unstable.

The final case to be considered is when $\lambda_I = 0, \lambda_E \neq 0$. In this situation, Eq. 70 tells us that $\boldsymbol{V}^\top \boldsymbol{w}_E^*$ is in the kernel of $\boldsymbol{A}^\top$ and therefore in the kernel of the diagonal matrix $\boldsymbol{A}\boldsymbol{A}^\top$ [2]. By using Eq. 68, we can therefore conclude that

$$\boldsymbol{A}\boldsymbol{A}^\top \Lambda(\boldsymbol{V}^\top \boldsymbol{w}_E^* - \boldsymbol{A}\boldsymbol{w}_I^*) = 0. \quad [75]$$

We work under the assumption that, in the absence of fine tuning, Λ has distinct non-zero eigenvalues. In this case, the first term in Equation 75 is zero, and $\boldsymbol{A}\boldsymbol{w}_I^*$ must also be in the kernel of $\boldsymbol{A}\boldsymbol{A}^\top$ and therefore in the kernel of $\boldsymbol{A}^\top$. So $\boldsymbol{w}_I^*$ is in the kernel of $\boldsymbol{A}^\top\boldsymbol{A}$ and therefore the kernel of $\boldsymbol{A}$. By Equation 68, this tells us that $\Lambda\boldsymbol{V}^\top \boldsymbol{w}_E^* = \lambda_E \boldsymbol{V}^\top \boldsymbol{w}_E^*$, and therefore $\boldsymbol{V}^\top \boldsymbol{w}_E^*$ is an eigenvector of Λ with eigenvalue λ_E. We therefore arrive at a fixed point for the system in which $\boldsymbol{V}^\top \boldsymbol{w}_E^*$ is one-hot with support on the kernel of $\boldsymbol{A}\boldsymbol{A}^\top$, and $\boldsymbol{w}_I^*$ is in the kernel of $\boldsymbol{A}$. This implies $\boldsymbol{w}_I^{*\top}\boldsymbol{y}_I = 0$ (cf. Eq. 50) which is biologically implausible since we constrain synaptic weights $\boldsymbol{w}_I^*$ and firing rates $\boldsymbol{y}_I$ to be positive.

Under mild assumptions regarding Λ and $\boldsymbol{A}\boldsymbol{A}^\top$, we have thus exhaustively characterized the fixed points of the system.

2.2 Stability analysis

We first consider the stability of fixed points that are regular eigenvectors of the modified covariance matrix and discuss the case of non-eigenvector fixed points afterwards. With Eq. 45, for the Jacobian $\bar{\boldsymbol{J}}$ it follows (cf. Eq. 29)

$$\boldsymbol{\tau}\,\bar{\boldsymbol{J}}\Big|_* = \boldsymbol{\tau}\,\frac{\mathrm{d}\dot{\bar{\boldsymbol{w}}}}{\mathrm{d}\bar{\boldsymbol{w}}}\Big|_* = \left[\mathbb{1} - \frac{\bar{\boldsymbol{v}}_E^*\bar{\boldsymbol{c}}_E{}^\top}{\bar{\boldsymbol{c}}_E{}^\top\bar{\boldsymbol{v}}_E^*} - \frac{\bar{\boldsymbol{v}}_I^*\bar{\boldsymbol{c}}_I{}^\top}{\bar{\boldsymbol{c}}_I{}^\top\bar{\boldsymbol{v}}_I^*}\right]\left[\bar{\boldsymbol{C}} - \bar{\lambda}^*\mathbb{1}\right], \quad [76]$$

[1] Briefly, the two normalization conditions are $k_E = \boldsymbol{c}^\top \boldsymbol{w}_E^*$, and $k_I = \boldsymbol{c}^\top \boldsymbol{w}_I^* = \frac{\lambda_E}{\lambda_I}\boldsymbol{c}^\top\boldsymbol{A}^\top\boldsymbol{V}^\top\boldsymbol{w}_E^*$, where we used Eq. 70. Then, by inserting Eqs. 74 we get two linear equations for the two unknown components of $\boldsymbol{w}_E^*$, which can be solved in terms of $k_E, k_I, \lambda_i, \lambda_j$. We can then insert the solution for $\boldsymbol{w}_E^*$ into Eq. 70 to obtain $\boldsymbol{w}_I^*$, which together defines all components of the eigenvector.

[2] Note that $\ker(\boldsymbol{A}^\top) = \ker(\boldsymbol{A}\boldsymbol{A}^\top)$ and $\ker(\boldsymbol{A}) = \ker(\boldsymbol{A}^\top\boldsymbol{A})$, for any matrix $\boldsymbol{A}$.

where $\bar{\boldsymbol{v}}_E^*$ and $\bar{\boldsymbol{v}}_I^*$ are the excitatory and the inhibitory part of the eigenvector fixed point $\bar{\boldsymbol{w}}^* = \bar{\boldsymbol{v}}^*$ with eigenvalue $\bar{\lambda}^*$, with an additional set of zeros to reach the correct dimensionality of the vector (cf. Eq. 41). To find the eigenvalues $\bar{\lambda}_{\boldsymbol{J}}$ of the Jacobian, we switch to the eigenbasis of the modified covariance matrix[1]:

$$\Rightarrow \quad \bar{\boldsymbol{V}}^{-1}\,\boldsymbol{J}\Big|_*\,\bar{\boldsymbol{V}} = \bar{\boldsymbol{V}}^{-1}\boldsymbol{\tau}^{-1}\bar{\boldsymbol{V}}\bar{\boldsymbol{V}}^{-1}\left[\mathbb{1} - \frac{\bar{\boldsymbol{v}}_E^*\bar{\boldsymbol{c}}_E{}^\top}{\bar{\boldsymbol{c}}_E{}^\top\bar{\boldsymbol{v}}_E^*} - \frac{\bar{\boldsymbol{v}}_I^*\bar{\boldsymbol{c}}_I{}^\top}{\bar{\boldsymbol{c}}_I{}^\top\bar{\boldsymbol{v}}_I^*}\right]\bar{\boldsymbol{V}}\left[\bar{\Lambda} - \bar{\lambda}^*\mathbb{1}\right], \qquad [77]$$

where we inserted $\bar{\boldsymbol{V}}\bar{\boldsymbol{V}}^{-1} \equiv \mathbb{1}$. The result is a block triangular matrix where each block on the diagonal corresponds to one regular eigenvector and its potentially multiple null eigenvectors. To better see this, we consider the first and second part of Eq. 77 separately. We define $\bar{\boldsymbol{\epsilon}} \equiv \bar{\boldsymbol{\tau}}^{-1}$, which remains a diagonal matrix with time constants for excitatory and inhibitory synapses on the diagonal, $\boldsymbol{\epsilon}_E = \mathbb{1}\epsilon_E$ and $\boldsymbol{\epsilon}_I = \mathbb{1}\epsilon_I$. Inserting the definition of the eigenvectors matrix and its inverse (Eq. 52) we write

$$\bar{\boldsymbol{V}}^{-1}\bar{\boldsymbol{\tau}}^{-1}\bar{\boldsymbol{V}} = \begin{pmatrix}(\mathbb{1}-\boldsymbol{A}\boldsymbol{A}^\top)^{-1} & \boldsymbol{0}\\ \boldsymbol{0} & (\mathbb{1}-\boldsymbol{A}^\top\boldsymbol{A})^{-1}\end{pmatrix}\begin{pmatrix}\boldsymbol{V}^\top & -\boldsymbol{A}\\ -\boldsymbol{A}^\top\boldsymbol{V}^\top & \mathbb{1}\end{pmatrix}\begin{pmatrix}\boldsymbol{\epsilon}_E & \boldsymbol{0}\\ \boldsymbol{0} & \boldsymbol{\epsilon}_I\end{pmatrix}\begin{pmatrix}\boldsymbol{V} & \boldsymbol{V}\boldsymbol{A}\\ \boldsymbol{A}^\top & \mathbb{1}\end{pmatrix} \qquad [78]$$

$$= \begin{pmatrix}(\mathbb{1}-\boldsymbol{A}\boldsymbol{A}^\top)^{-1} & \boldsymbol{0}\\ \boldsymbol{0} & (\mathbb{1}-\boldsymbol{A}^\top\boldsymbol{A})^{-1}\end{pmatrix}\begin{pmatrix}\boldsymbol{\epsilon}_E - \boldsymbol{\epsilon}_I\boldsymbol{A}\boldsymbol{A}^\top & (\boldsymbol{\epsilon}_E-\boldsymbol{\epsilon}_I)\boldsymbol{A}\\ (\boldsymbol{\epsilon}_I-\boldsymbol{\epsilon}_E)\boldsymbol{A}^\top & \boldsymbol{\epsilon}_I - \boldsymbol{\epsilon}_E\boldsymbol{A}^\top\boldsymbol{A}\end{pmatrix}. \qquad [79]$$

As one would expect, for $\boldsymbol{\epsilon}_E = \boldsymbol{\epsilon}_I$, this is equal to a scalar times the identity matrix. When we switch columns and rows such that pairs of regular and corresponding null eigenvectors form blocks, this becomes a block diagonal matrix. Note that this does not change the determinant or the eigenvalues of the matrix as for each row switch, there is a corresponding column switch that maintains the characteristic polynomial. Alternatively, we can assume that the matrix of eigenvectors $\bar{\boldsymbol{V}}$ and its inverse $\bar{\boldsymbol{V}}^{-1}$ are already appropriately sorted. Without loss of generality, we assume that the first columns of $\bar{\boldsymbol{V}}$ are the fixed point's eigenvector $\bar{\boldsymbol{v}}^*$ and its corresponding null eigenvectors, and write[2]

$$\bar{\boldsymbol{V}}^{-1}\bar{\boldsymbol{\tau}}^{-1}\bar{\boldsymbol{V}} = \begin{pmatrix}(1-\boldsymbol{a}^{*\top}\boldsymbol{a}^*)^{-1} & \boldsymbol{0} & \boldsymbol{0}\\ \boldsymbol{0} & (1-\boldsymbol{a}^*\boldsymbol{a}^{*\top})^{-1} & \\ \boldsymbol{0} & & \ddots\end{pmatrix}\begin{pmatrix}\epsilon_E - \epsilon_I\boldsymbol{a}^{*\top}\boldsymbol{a}^* & (\epsilon_E-\epsilon_I)\boldsymbol{a}^{*\top} & \boldsymbol{0}\\ (\epsilon_I-\epsilon_E)\boldsymbol{a}^* & \epsilon_I - \epsilon_E\boldsymbol{a}^*\boldsymbol{a}^{*\top} & \\ \boldsymbol{0} & & \ddots\end{pmatrix}, \qquad [80]$$

where $\boldsymbol{a}^*$ is a column vector that holds the multiples of the inhibitory neurons that are tuned to the feedforward eigenvector $\boldsymbol{v}^*$. As before, $\boldsymbol{0}$ are matrices of zeros and appropriate dimensionality, and ellipsis indicate continuing blocks on the diagonal with similar terms that belong to the non-fixed point eigenvectors and their null eigenvectors[3].

Similarly, we can write the second part of Eq. 77 as a block triangular matrix. Before sorting, we write

$$\bar{\boldsymbol{V}}^{-1}\left[\frac{\bar{\boldsymbol{v}}_E^*\bar{\boldsymbol{c}}_E{}^\top}{\bar{\boldsymbol{c}}_E{}^\top\bar{\boldsymbol{v}}_E^*} + \frac{\bar{\boldsymbol{v}}_I^*\bar{\boldsymbol{c}}_I{}^\top}{\bar{\boldsymbol{c}}_I{}^\top\bar{\boldsymbol{v}}_I^*}\right]\bar{\boldsymbol{V}} \equiv \bar{\boldsymbol{V}}^{-1}\left[\bar{\boldsymbol{v}}_E^*\boldsymbol{d}_E{}^\top + \bar{\boldsymbol{v}}_I^*\boldsymbol{d}_I{}^\top\right] = \bar{\boldsymbol{V}}^{-1}\begin{pmatrix}\boldsymbol{v}_E^*\boldsymbol{d}_E{}^\top\\ \boldsymbol{v}_I^*\boldsymbol{d}_I{}^\top\end{pmatrix}, \qquad [81]$$

$$\boldsymbol{d}_E{}^\top = \frac{\bar{\boldsymbol{c}}_E{}^\top\bar{\boldsymbol{V}}}{\bar{\boldsymbol{c}}_E{}^\top\bar{\boldsymbol{v}}_E^*}, \quad \boldsymbol{d}_I{}^\top = \frac{\bar{\boldsymbol{c}}_I{}^\top\bar{\boldsymbol{V}}}{\bar{\boldsymbol{c}}_I{}^\top\bar{\boldsymbol{v}}_I^*}, \quad \boldsymbol{v}_E^* = \boldsymbol{V}\boldsymbol{e}^*, \quad \boldsymbol{v}_I^* = \boldsymbol{A}^\top\boldsymbol{e}^*, \qquad [82]$$

where $\boldsymbol{d}_E{}^\top$ and $\boldsymbol{d}_I{}^\top$ are row vectors that hold the L1-norms of the eigenvectors' excitatory and inhibitory parts as a fraction of the L1-norm of the fixed point eigenvector's excitatory and inhibitory parts. The vector $\boldsymbol{e}^*$ is zero except for one entry, equal to one, which corresponds to the fixed point feedforward eigenvector $\boldsymbol{v}^*$. We continue by multiplying the inverse eigenvector matrix $\bar{\boldsymbol{V}}^{-1}$ from the left:

$$\bar{\boldsymbol{V}}^{-1}\begin{pmatrix}\boldsymbol{v}_E^*\boldsymbol{d}_E{}^\top\\ \boldsymbol{v}_I^*\boldsymbol{d}_I{}^\top\end{pmatrix} = \boldsymbol{N}\begin{pmatrix}\boldsymbol{V}^\top & -\boldsymbol{A}\\ -\boldsymbol{A}^\top\boldsymbol{V}^\top & \mathbb{1}\end{pmatrix}\begin{pmatrix}\boldsymbol{V}\boldsymbol{e}^*\boldsymbol{d}_E{}^\top\\ \boldsymbol{A}^\top\boldsymbol{e}^*\boldsymbol{d}_I{}^\top\end{pmatrix} = \boldsymbol{N}\begin{pmatrix}\boldsymbol{e}^*\boldsymbol{d}_E{}^\top - \boldsymbol{A}\boldsymbol{A}^\top\boldsymbol{e}^*\boldsymbol{d}_I{}^\top\\ -\boldsymbol{A}^\top\boldsymbol{e}^*\boldsymbol{d}_E{}^\top + \boldsymbol{A}^\top\boldsymbol{e}^*\boldsymbol{d}_I{}^\top\end{pmatrix}, \qquad [83]$$

[1] Note that we must make use of the inverse instead of the transpose since, in general, the eigenvector matrix $\bar{\boldsymbol{V}}$ is not orthonormal.

[2] Note that when sorted, $\boldsymbol{A}^\top\boldsymbol{A}$ is a block diagonal matrix. Further, as noted before, the matrix $\boldsymbol{A}\boldsymbol{A}^\top$ is always diagonal.

[3] The dimensionalities of these blocks depend on the number inhibitory neurons tuned to the respective feedforward eigenvector, i.e., if there are $n_I^\dagger$ inhibitory neurons tuned to a specific feedforward eigenvector $\boldsymbol{v}^\dagger$, the dimensionality of the corresponding block is $1+n_I$, due to one regular eigenvector and $n_I^\dagger$ corresponding null eigenvectors.

where we defined the normalization matrix $\boldsymbol{N}$ of the inverse eigenvector matrix $\bar{\boldsymbol{V}}^{-1}$ (cf. Eq. 52) to improve readability. It follows that the matrix above holds non-zero values in only a few rows, corresponding to the fixed point eigenvector (top block) and its null eigenvectors (bottom block). After rearranging, we get

$$\boldsymbol{N}\begin{pmatrix} \boldsymbol{e}^*\boldsymbol{d}_E^\top - \boldsymbol{A}\boldsymbol{A}^\top\boldsymbol{e}^*\boldsymbol{d}_I^\top \\ -\boldsymbol{A}^\top\boldsymbol{e}^*\boldsymbol{d}_E^\top + \boldsymbol{A}^\top\boldsymbol{e}^*\boldsymbol{d}_I^\top \end{pmatrix} = \boldsymbol{N}\begin{pmatrix} d_E^* - \boldsymbol{a}^{*\top}\boldsymbol{a}^* d_I^* & \boldsymbol{d}_E^{\circledast\top} - \boldsymbol{a}^{*\top}\boldsymbol{a}^*\boldsymbol{d}_I^{\circledast\top} & \dots \\ -\boldsymbol{a}^* d_E^* + \boldsymbol{a}^* d_I^* & -\boldsymbol{a}^*\boldsymbol{d}_E^{\circledast\top} + \boldsymbol{a}^*\boldsymbol{d}_I^{\circledast\top} & \\ & \boldsymbol{0} & \boldsymbol{0} \end{pmatrix} \quad [84]$$

where d_E^*, d_I^* and $\boldsymbol{d}_E^{\circledast\top}$, $\boldsymbol{d}_I^{\circledast\top}$ are the entries of $\boldsymbol{d}_E^\top$, $\boldsymbol{d}_I^\top$ that correspond to the fixed point eigenvector and its null eigenvectors, respectively. As before, ellipsis indicate additional non-zero entries. To find the respective entries of $\boldsymbol{d}_E$, $\boldsymbol{d}_I$ we use the definition of $\bar{\boldsymbol{V}}$ (Eq. 52) to write

$$\boldsymbol{d}_E^\top = \frac{\bar{\boldsymbol{c}}_E^\top\bar{\boldsymbol{V}}}{\bar{\boldsymbol{c}}_E^\top\bar{\boldsymbol{v}}_E^*} = \frac{1}{\boldsymbol{c}_E^\top\boldsymbol{v}^*}\left(\boldsymbol{c}_E^\top\boldsymbol{V},\ \boldsymbol{c}_E^\top\boldsymbol{V}\boldsymbol{A}\right), \quad [85]$$

$$\boldsymbol{d}_I^\top = \frac{\bar{\boldsymbol{c}}_I^\top\bar{\boldsymbol{V}}}{\bar{\boldsymbol{c}}_I^\top\bar{\boldsymbol{v}}_I^*} = \frac{1}{\boldsymbol{c}_I^\top\boldsymbol{A}^\top\boldsymbol{e}^*}\left(\boldsymbol{c}_I^\top\boldsymbol{A}^\top,\ \boldsymbol{c}_I^\top\mathbb{1}\right). \quad [86]$$

After rearranging the entries that correspond to the fixed point eigenvector and its null eigenvectors to the front we get

$$\boldsymbol{d}_E^\top = \left(d_E^*,\ \boldsymbol{d}_E^{\circledast\top},\ \dots\right) = \frac{1}{\boldsymbol{c}_E^\top\boldsymbol{v}^*}\left(\boldsymbol{c}_E^\top\boldsymbol{V}\boldsymbol{e}^*,\ \boldsymbol{c}_E^\top\boldsymbol{v}^*\boldsymbol{a}^{*\top}\right) = \left(1,\ \boldsymbol{a}^{*\top},\ \dots\right), \quad [87]$$

$$\boldsymbol{d}_I^\top = \left(d_I^*,\ \boldsymbol{d}_I^{\circledast\top},\ \dots\right) = \frac{1}{\boldsymbol{c}_I^\top\boldsymbol{A}^\top\boldsymbol{e}^*}\left(\boldsymbol{c}_I^\top\boldsymbol{A}^\top\boldsymbol{e}^*,\ \boldsymbol{c}_I^\top\right) = \left(1,\ \frac{\boldsymbol{c}_I^\top}{\boldsymbol{c}^\top\boldsymbol{a}^*},\ \dots\right), \quad [88]$$

where $\boldsymbol{e}^*$ selects the proper columns and $\boldsymbol{c}^\top$ is a row vector of ones of appropriate dimensionality. We insert Eq. 87 & 88 into Eq. 84 and find

$$\bar{\boldsymbol{V}}^{-1}\left[\frac{\bar{\boldsymbol{v}}_E^*\bar{\boldsymbol{c}}_E^\top}{\bar{\boldsymbol{c}}_E^\top\bar{\boldsymbol{v}}_E^*} + \frac{\bar{\boldsymbol{v}}_I^*\bar{\boldsymbol{c}}_I^\top}{\bar{\boldsymbol{c}}_I^\top\bar{\boldsymbol{v}}_I^*}\right]\bar{\boldsymbol{V}} = \boldsymbol{N}\begin{pmatrix} 1 - \boldsymbol{a}^{*\top}\boldsymbol{a}^* & \boldsymbol{0} & \\ \boldsymbol{0} & \frac{\boldsymbol{a}^*\boldsymbol{c}_I^\top}{\boldsymbol{c}^\top\boldsymbol{a}^*} - \boldsymbol{a}^*\boldsymbol{a}^{*\top} & \dots \\ & \boldsymbol{0} & \boldsymbol{0} \end{pmatrix} \equiv \begin{pmatrix} 1 & \boldsymbol{0} & \\ \boldsymbol{0} & \boldsymbol{M}^* & \dots \\ & \boldsymbol{0} & \boldsymbol{0} \end{pmatrix}, \quad [89]$$

$$\boldsymbol{M}^* = \left(\mathbb{1} - \boldsymbol{a}^*\boldsymbol{a}^\top\right)^{-1}\left(\frac{\boldsymbol{a}^*\boldsymbol{c}_I^\top}{\boldsymbol{c}^\top\boldsymbol{a}^*} - \boldsymbol{a}^*\boldsymbol{a}^{*\top}\right), \quad [90]$$

where we defined the matrix $\boldsymbol{M}^*$.

In summary, we find that after rearrangement, Eq. 77 is a block triangular matrix.

$$\Rightarrow \quad \bar{\boldsymbol{V}}^{-1}\,\boldsymbol{J}\Big|_*\,\bar{\boldsymbol{V}} = \boldsymbol{N}\begin{pmatrix} \epsilon_E - \epsilon_I\boldsymbol{a}^{*\top}\boldsymbol{a}^* & (\epsilon_E - \epsilon_I)\boldsymbol{a}^{*\top} & \boldsymbol{0} \\ (\epsilon_I - \epsilon_E)\boldsymbol{a}^* & \epsilon_I - \epsilon_E\boldsymbol{a}^*\boldsymbol{a}^{*\top} & \\ & \boldsymbol{0} & \ddots \end{pmatrix}\begin{pmatrix} 0 & \boldsymbol{0} & \\ \boldsymbol{0} & \mathbb{1} - \boldsymbol{M}^* & \dots \\ & \boldsymbol{0} & \mathbb{1} \end{pmatrix}\left[\bar{\Lambda} - \bar{\lambda}^*\mathbb{1}\right], \quad [91]$$

where we used Eq. 80 and Eq. 89. Therefore, to find the eigenvalues, we consider each diagonal block separately. We make the simplifying assumption that there is exactly one inhibitory neuron tuned to each feedforward eigenvector. Then, $\boldsymbol{a}^* \to a^*$ becomes a scalar, $\boldsymbol{N}$ and $\boldsymbol{A} = \boldsymbol{A}^\top$ become diagonal, and $\boldsymbol{M}^* \to 1$. The transformed Jacobian remains triangular and becomes

$$\Rightarrow \quad \bar{\boldsymbol{V}}^{-1}\,\boldsymbol{J}\Big|_*\,\bar{\boldsymbol{V}} = \boldsymbol{N}\begin{pmatrix} \epsilon_E - \epsilon_I a^{*2} & (\epsilon_E - \epsilon_I)a^* & \boldsymbol{0} \\ (\epsilon_I - \epsilon_E)a^* & \epsilon_I - \epsilon_E a^{*2} & \\ & \boldsymbol{0} & \ddots \end{pmatrix}\begin{pmatrix} 0 & 0 & \\ 0 & 0 & \dots \\ & \boldsymbol{0} & \mathbb{1} \end{pmatrix}\left[\bar{\Lambda} - \bar{\lambda}^*\mathbb{1}\right], \quad [92]$$

with 2×2 blocks on the diagonal of which we only show the first, that corresponds to perturbations in the direction of the fixed point eigenvector or its null eigenvector[1]. From the matrix product above, we see that their corresponding

[1] Since we assumed that there is exactly one inhibitory neuron per feedforward eigenvector, there is also exactly one null eigenvector per feedforward eigenvector (cf. Eq. 52)

eigenvalues must be zero since the first two columns of the second to last matrix are zero. For perturbations in the direction of a non-fixed point eigenvector $\bar{\boldsymbol{v}}^\dagger$ or its null eigenvector we have to consider the block matrix

$$\boldsymbol{J}_*^\dagger \equiv \bar{\boldsymbol{V}}^{\dagger-1}\,\bar{\boldsymbol{J}}\Big|_*\,\bar{\boldsymbol{V}}^\dagger = \frac{1}{1-a^{\dagger 2}}\begin{pmatrix}\epsilon_E - \epsilon_I a^{\dagger 2} & \epsilon_E - \epsilon_I \\ (-\epsilon_E + \epsilon_I)a^{\dagger 2} & -\epsilon_E a^{\dagger 2} + \epsilon_I\end{pmatrix}\begin{pmatrix}\bar{\lambda}^\dagger - \bar{\lambda}^* & 0 \\ 0 & -\bar{\lambda}^*\end{pmatrix}, \quad [93]$$

where $\bar{\boldsymbol{V}}^\dagger$ is a two-column matrix that holds $\bar{\boldsymbol{v}}^\dagger$ and its null eigenvector. The eigenvalues of this matrix are negative under two conditions. First, its determinant must be positive, and second, its trace must be negative. For trace and determinant, we find

$$\det(\bar{\boldsymbol{J}}_*^\dagger) = \frac{1}{\left(1-a^{\dagger 2}\right)^2}\left(1 - 2a^{\dagger 2} + a^{\dagger 4}\right)\epsilon_E\epsilon_I\left(\bar{\lambda}^* - \bar{\lambda}^\dagger\right)\bar{\lambda}^*, \quad [94]$$

$$\mathrm{tr}(\boldsymbol{J}_*^\dagger) = \frac{1}{\left(1-a^{\dagger 2}\right)}\left(\epsilon_E\left[\bar{\lambda}^\dagger - \left(1-a^{\dagger 2}\right)\bar{\lambda}^*\right] + \epsilon_I\left[-a^{\dagger 2}\bar{\lambda}^\dagger - \left(1-a^{\dagger 2}\right)\bar{\lambda}^*\right]\right) \quad [95]$$

Finally, the two stability conditions read

$$\det(\boldsymbol{J}_*^\dagger) \overset{!}{>} 0 \quad \Rightarrow \quad -\left(\bar{\lambda}^\dagger - \bar{\lambda}^*\right)\bar{\lambda}^*\epsilon_E\epsilon_I > 0, \quad [96]$$

$$\mathrm{tr}(\boldsymbol{J}_*^\dagger) \overset{!}{<} 0 \quad \Rightarrow \quad \epsilon_E(\lambda^\dagger - \bar{\lambda}^*) - \epsilon_I\left(a^{\dagger 2}\lambda^\dagger + \bar{\lambda}^*\right) < 0, \quad [97]$$

where, for the trace term, we made use of the equality $\bar{\lambda}^\dagger = \lambda^\dagger\left(1 - a^{\dagger 2}\right)$ (cf. Eq. 53) to replace $\bar{\lambda}^\dagger$.

2.2.1 Principal component analysis in inhibition modified input space

The first stability condition above states that only the fixed point $\bar{\boldsymbol{v}}^*$ with the largest eigenvalue, $\bar{\lambda}^* > \bar{\lambda}^\dagger, \forall\bar{\lambda}^\dagger$, can be stable, and then only if it is not repulsive, i.e., provided that its corresponding eigenvalue is larger than zero. An eigenvector can become repulsive if inhibition is sufficiently strong, i.e., if $\bar{\lambda}^* = \lambda^*(1 - a^{*2}) < 0 \Rightarrow a^{*2} > 1$. This implies that post-synaptic neurons tuned to repulsive eigenvectors receive more inhibition than excitation, which results in negative firing rates $r^* = \boldsymbol{y_E}^\mathsf{T}\boldsymbol{v}^* - \boldsymbol{y_E}^\mathsf{T}\boldsymbol{v}^*a^{*2} < 0$, for $a^{*2} > 1$ (cf. Eq. 55). However, in biology, neurons with larger inhibitory than excitatory input are hyperpolarized and remain silent, which is why we assume $\bar{\lambda}^* > 0$. In the following, we call the combination of the excitatory feedforward attraction of an eigenvector λ^* (cf. Sec. 1.1) plus any contribution of laterally projecting neurons, in this case, minus the lateral inhibitory repulsion $a^{*2}\lambda^*$, the effective attraction, $\bar{\lambda}^*$, of a feedforward input mode.

For $\epsilon_I = \epsilon_E$, the second condition reduces to[1] $\bar{\lambda}^\dagger - 2\bar{\lambda}^* < 0$, which holds if the first condition is met. Therefore, the post-synaptic neuron becomes tuned to the eigenvector of the modified covariance matrix with the largest eigenvalue, i.e., it performs principal component analysis on a modified feedforward input space, where the attraction of feedforward eigenvectors is modified by laterally projecting inhibitory neurons (cf. Eq. 53). We will further discuss the notion of a modified input space in Section 3.

2.2.2 Fast inhibition increases stability

In our networks, stationary states can still emerge when inhibitory plasticity is slower than excitatory plasticity. In the extreme case of static inhibition, $\epsilon_I = 0$, the second stability condition is still satisfied if the fixed point attraction $\bar{\lambda}^*$ is larger than the feedforward attraction $\lambda^\dagger$ of any other eigenvector, $\lambda^\dagger - \bar{\lambda}^* < 0$. When inhibitory weights are static, they remain tuned to the fixed point and the repulsive component of competing eigenvectors $a^{\dagger 2}\lambda^\dagger$ do not matter for stability. This explains why we have to consider only the attractive part $\lambda^\dagger$ of the effective attraction $\bar{\lambda}^\dagger$ in the first term of the second stability condition. However, for growing $\epsilon_I > 0$, the influence of the inhibitory part of competing eigenvectors increases, corresponding to an increasingly negative second term in the second stability condition[2]. Then, for sufficiently fast inhibitory plasticity $\epsilon_I > \epsilon_E$, the second condition always holds. Therefore, we consider slightly faster inhibitory than excitatory plasticity in our numerical simulations (cf. Table 1).

[1] Here, we make use of the equality $\bar{\lambda}^\dagger = \lambda^\dagger\left(1 - a^{\dagger 2}\right)$.

[2] Note that we consider non-repulsive fixed points $\bar{\lambda}^* > 0$ and inhibitory neurons with positive firing rates, i.e., $a > 0, \forall a$, such that the second term in the second stability condition is always negative.

2.2.3 Stability of non-eigenvector fixed points

Before, we considered the stability of fixed points $\bar{\boldsymbol{w}}^*$ that are eigenvectors $\bar{\boldsymbol{v}}$ of the modified covariance matrix $\bar{\boldsymbol{C}}$. Weight vectors of that shape put a strong constraint on the choice of the weight norms, as the ratio between the excitatory and the inhibitory weight norms is given by the norms of the excitatory and the inhibitory parts of the eigenvector (cf. Eq. 52). The issue was solved in that we found that arbitrary combinations of multiples of the excitatory and inhibitory components of regular eigenvectors are also fixed points (cf. Sec. 2.1.3). We will now consider the stability of such non-eigenvector fixed points.

Let the shape of a fixed point $\bar{\boldsymbol{w}}^*$ be (cf. Eq. 56)

$$\bar{\boldsymbol{w}}^* = \begin{pmatrix} k_E \boldsymbol{v}_E^* \\ k_I \boldsymbol{v}_I^* \end{pmatrix} = \begin{pmatrix} \mathbb{1} k_E & \boldsymbol{0} \\ \boldsymbol{0} & \mathbb{1} k_I \end{pmatrix} \bar{\boldsymbol{v}}^* \equiv \boldsymbol{K} \bar{\boldsymbol{v}}^*, \qquad [98]$$

where k_E and k_I are scalar constants. We recapitulate the general weight dynamics as given in Eq. 45:

$$\bar{\boldsymbol{\tau}} \dot{\bar{\boldsymbol{w}}} = \left[\mathbb{1} - \frac{\bar{\boldsymbol{w}}_E \bar{\boldsymbol{c}}_E{}^\mathsf{T}}{\bar{\boldsymbol{c}}_E{}^\mathsf{T} \bar{\boldsymbol{w}}_E} - \frac{\bar{\boldsymbol{w}}_I \bar{\boldsymbol{c}}_I{}^\mathsf{T}}{\bar{\boldsymbol{c}}_I{}^\mathsf{T} \bar{\boldsymbol{w}}_I} \right] \bar{\boldsymbol{C}} \bar{\boldsymbol{w}}. \qquad [99]$$

Instead of evaluating the eigenvalues of the Jacobian, we now switch to a new coordinate system in which the Jacobian will have a familiar shape. This is possible since fixed points and their stability do not depend on the choice of coordinates. We define:

$$\bar{\boldsymbol{w}}' \equiv \boldsymbol{K}^{-1/2} \bar{\boldsymbol{w}}, \quad \Rightarrow \bar{\boldsymbol{w}} = \boldsymbol{K}^{1/2} \bar{\boldsymbol{w}}', \qquad [100]$$

from which the weight dynamics can be written as

$$\dot{\bar{\boldsymbol{w}}}' = \boldsymbol{K}^{-1/2} \dot{\bar{\boldsymbol{w}}} = \boldsymbol{K}^{-1/2} \bar{\boldsymbol{\tau}}^{-1} \left[\mathbb{1} - \frac{\boldsymbol{K}^{1/2} \bar{\boldsymbol{w}}'_E \bar{\boldsymbol{c}}_E{}^\mathsf{T}}{\bar{\boldsymbol{c}}_E{}^\mathsf{T} \boldsymbol{K}^{1/2} \bar{\boldsymbol{w}}'_E} - \frac{\boldsymbol{K}^{1/2} \bar{\boldsymbol{w}}'_I \bar{\boldsymbol{c}}_I{}^\mathsf{T}}{\bar{\boldsymbol{c}}_I{}^\mathsf{T} \boldsymbol{K}^{1/2} \bar{\boldsymbol{w}}'_I} \right] \boldsymbol{K}^{-1/2} \boldsymbol{K}^{1/2} \bar{\boldsymbol{C}} \boldsymbol{K}^{1/2} \bar{\boldsymbol{w}}', \qquad [101]$$

where we inserted $\boldsymbol{K}^{-1/2}\boldsymbol{K}^{1/2} = \mathbb{1}$. We now make use of the following identities:

$$\bar{\boldsymbol{c}}_A{}^\mathsf{T} \boldsymbol{K}^{1/2} \bar{\boldsymbol{w}}'_A = k_A^{1/2} \bar{\boldsymbol{c}}_A{}^\mathsf{T} \bar{\boldsymbol{w}}'_A, \quad \boldsymbol{K}^{1/2} \bar{\boldsymbol{w}}'_A \bar{\boldsymbol{c}}_A{}^\mathsf{T} = k_A^{1/2} \bar{\boldsymbol{w}}'_A \bar{\boldsymbol{c}}_A{}^\mathsf{T}, \quad \frac{\bar{\boldsymbol{w}}'_A \bar{\boldsymbol{c}}_A{}^\mathsf{T}}{\bar{\boldsymbol{c}}_A{}^\mathsf{T} \bar{\boldsymbol{w}}'_A} \boldsymbol{K}^{-1/2} = \boldsymbol{K}^{-1/2} \frac{\bar{\boldsymbol{w}}'_A \bar{\boldsymbol{c}}_A{}^\mathsf{T}}{\bar{\boldsymbol{c}}_A{}^\mathsf{T} \bar{\boldsymbol{w}}'_A}, \quad \forall A \in \{E, I\}. \qquad [102]$$

We find that the $\boldsymbol{K}^{1/2}$ matrices inside the bracket cancel, and we can pull $\boldsymbol{K}^{-1/2}$ from the right side to the left side of the bracket:

$$\dot{\bar{\boldsymbol{w}}}' = \boldsymbol{K}^{-1/2} \dot{\bar{\boldsymbol{w}}} = \boldsymbol{K}^{-1/2} \bar{\boldsymbol{\tau}}^{-1} \boldsymbol{K}^{-1/2} \left[\mathbb{1} - \frac{\bar{\boldsymbol{w}}'_E \bar{\boldsymbol{c}}_E{}^\mathsf{T}}{\bar{\boldsymbol{c}}_E{}^\mathsf{T} \bar{\boldsymbol{w}}'_E} - \frac{\bar{\boldsymbol{w}}'_I \bar{\boldsymbol{c}}_I{}^\mathsf{T}}{\bar{\boldsymbol{c}}_I{}^\mathsf{T} \bar{\boldsymbol{w}}'_I} \right] \boldsymbol{K}^{1/2} \bar{\boldsymbol{C}} \boldsymbol{K}^{1/2} \bar{\boldsymbol{w}}'. \qquad [103]$$

We introduce the following definitions

$$\bar{\boldsymbol{\tau}}' = \bar{\boldsymbol{\tau}} \boldsymbol{K}, \quad \bar{\boldsymbol{C}}' = \boldsymbol{K}^{1/2} \bar{\boldsymbol{C}} \boldsymbol{K}^{1/2} = \left\langle \begin{pmatrix} k_E^{1/2} \mathbf{y}_E \\ k_I^{1/2} \mathbf{y}_I \end{pmatrix} \left(k_E^{1/2} \mathbf{y}_E{}^\mathsf{T}, -k_I^{1/2} \mathbf{y}_I{}^\mathsf{T} \right)^\mathsf{T} \right\rangle. \qquad [104]$$

Note that $\bar{\boldsymbol{C}}'$ is *not* the modified covariance matrix expressed in the new coordinate system but a new modified covariance matrix that corresponds to an altered input space where excitatory and inhibitory input firing rates $\boldsymbol{y}_E, \boldsymbol{y}_I$ are scaled by $k_E^{1/2}, k_I^{1/2}$, respectively. In summary, we can write the plasticity of the weight vector in the new coordinate system as[1]

$$\boxed{\bar{\boldsymbol{\tau}}' \dot{\bar{\boldsymbol{w}}}' = \left[\mathbb{1} - \frac{\bar{\boldsymbol{w}}'_E \bar{\boldsymbol{c}}_E{}^\mathsf{T}}{\bar{\boldsymbol{c}}_E{}^\mathsf{T} \bar{\boldsymbol{w}}'_E} - \frac{\bar{\boldsymbol{w}}'_I \bar{\boldsymbol{c}}_I{}^\mathsf{T}}{\bar{\boldsymbol{c}}_I{}^\mathsf{T} \bar{\boldsymbol{w}}'_I} \right] \bar{\boldsymbol{C}}' \bar{\boldsymbol{w}}'}\ . \qquad [105]$$

We are interested in the stability of the fixed points given in Eq. 98. In the new coordinate system, they become

$$\bar{\boldsymbol{w}}'^* = \boldsymbol{K}^{-1/2} \bar{\boldsymbol{w}}^* = \boldsymbol{K}^{-1/2} \boldsymbol{K} \bar{\boldsymbol{v}}^* = \boldsymbol{K}^{1/2} \bar{\boldsymbol{v}}^*. \qquad [106]$$

[1] Here, $\bar{\boldsymbol{\tau}}^{-1}$ and $\boldsymbol{K}^{-1/2}$ are both diagonal matrices and commute.

It is straightforward to proof that $\bar{\boldsymbol{w}}'^*$ is an eigenvector of the new modified covariance matrix $\bar{\boldsymbol{C}}'$ with eigenvalue $\bar{\lambda}'^* = (k_E - k_I a^{*2})\lambda^*$: With $\bar{\boldsymbol{C}}$ defined in Eq. 51 we get

$$\bar{\boldsymbol{C}}'\bar{\boldsymbol{w}}'^* = \boldsymbol{K}^{1/2}\bar{\boldsymbol{C}}\boldsymbol{K}^{1/2}\boldsymbol{K}^{1/2}\bar{\boldsymbol{v}}^* \quad [107]$$

$$= \boldsymbol{K}^{1/2}\begin{pmatrix} \boldsymbol{C} & -\boldsymbol{V}\boldsymbol{\Lambda}\boldsymbol{A} \\ \boldsymbol{A}^\top\boldsymbol{\Lambda}\boldsymbol{V}^\top & -\boldsymbol{A}^\top\boldsymbol{\Lambda}\boldsymbol{A}\end{pmatrix}\begin{pmatrix}\boldsymbol{V}\boldsymbol{e}^* k_E \\ \boldsymbol{A}^\top\boldsymbol{e}^* k_I\end{pmatrix} \quad [108]$$

$$= \boldsymbol{K}^{1/2}\begin{pmatrix}\boldsymbol{V}\boldsymbol{e}^*\lambda^* k_E - \boldsymbol{V}\boldsymbol{\Lambda}\boldsymbol{A}\boldsymbol{A}^\top\boldsymbol{e}^* k_I \\ \boldsymbol{A}^\top\boldsymbol{e}^*\lambda^* k_E - \boldsymbol{A}^\top\boldsymbol{\Lambda}\boldsymbol{A}\boldsymbol{A}^\top\boldsymbol{e}^* k_I\end{pmatrix} \quad [109]$$

$$= \boldsymbol{K}^{1/2}\begin{pmatrix}\boldsymbol{V}\boldsymbol{e}^* \\ \boldsymbol{A}^\top\boldsymbol{e}^*\end{pmatrix}(k_E - k_I a^{*2})\lambda^* = \bar{\boldsymbol{w}}'^*\bar{\lambda}'^*, \quad [110]$$

where we defined a^{*2} as the entry of the diagonal matrix $\boldsymbol{A}\boldsymbol{A}^\top$ that corresponds to the eigenvector $\boldsymbol{v}^*$[1]. Note that this is independent of the change of variables, however, only in the new coordinate system one can identify the new modified covariance matrix with an actual input space[2], where pre-synaptic firing rates are scaled by k_E, k_I (Eq. 104). In theory, we can now proceed in finding the eigenvalues of the Jacobian[3], as explained in Section 2.2. . As before, one finds that stability is largely determined by the eigenvalues of the modified covariance matrix, which now are $\bar{\lambda}'$.

Apart from providing a principled way to determine if a non-eigenvector fixed point is stable, our formulation provides additional insight: Let's assume the total synaptic inhibitory weight of a neuron is very small, much smaller than any eigenvector of $\bar{\boldsymbol{C}}$ would suggest, i.e., $k_I \ll 1$, while the excitatory weight norm is equal to one, which implies $k_E = 1$. As one would expect intuitively, the neuron does not exhibit much of the repulsion of the inhibitory neurons (cf. Eq. 104 for $k_I \ll 1$), and its stability would be primarily determined by the excitatory attraction of the different eigenvector modes, i.e., $\bar{\lambda}' = (k_E - k_I a^{*2})\lambda \approx \lambda$. In the extreme case, when the inhibitory weight norm is zero, i.e., $k_I = 0$, only the activity of the excitatory population is relevant.

While the effective plasticity timescale $\bar{\boldsymbol{\tau}}' = \bar{\boldsymbol{\tau}}\boldsymbol{K}$ in Equation 105 depends on the magnitude of the excitatory and the inhibitory part of the specific fixed point under consideration, this does *not* mean that the speed of synaptic plasticity is different from the original formulation in Equation 45. For example, when we consider a fixed point with a decreased inhibitory weight norm $k_I < 1$, the effective inhibitory plasticity appears to increase, since $\bar{\tau}'_I = \tau_I k_I$. However, this effect is balanced by the decrease in pre-synaptic inhibitory firing rates, which decreases with decreasing k_I. Similarly, the coordinate system in which we describe the weight dynamics also does not affect the speed of plasticity[4]. From Equation 103 we see that we can freely shift scaling matrices $\boldsymbol{K}$ between the modified covariance matrix and the plasticity timescale by pulling diagonal matrices of the same shape as $\boldsymbol{K}$ through the bracket (cf. Eq. 102). However, in Section 2.2 we only considered the stability of fixed points that are regular eigenvectors of the modified covariance matrix. If we had chosen, e.g., $\bar{\boldsymbol{C}}' = \boldsymbol{K}^{-1/2}\bar{\boldsymbol{C}}\boldsymbol{K}^{1/2}$ and $\boldsymbol{\tau}' = \boldsymbol{\tau}$, then $\bar{\boldsymbol{w}}'^* = \boldsymbol{K}^{1/2}\bar{\boldsymbol{v}}^*$ (cf. Eq. 106) would not be a regular eigenvector of $\bar{\boldsymbol{C}}'$ (cf. Eq. 107f.). Therefore, our derivation in Section 2.2 would not apply, and we would need to find a different way to proof stability.

3 Lateral input stretches and compresses the feedforward input space

Before we consider how synapse-type-specific Hebbian plasticity affects learning in fully plastic recurrent networks, we first build additional intuition for how static lateral input affects the weight dynamics. From the previous section we know that in this case the eigenvalues of the modified covariance matrix are the key factors that determine fixed point stability, and from Sections 1.1 & 2.1.2 we know that these eigenvalues describe the Hebbian growth towards the corresponding eigenvector that can be attractive or repulsive, corresponding to a positive or negative eigenvalue. When a neuron receives only feedforward excitatory input (Fig. S2*A*), the weight dynamics is described by a true covariance matrix with eigenvalues equal to the variances along the principal components of the feedforward input space (cf. Sec. 1). Then the weight vector in the fixed point aligns with the direction of maximal variance in the

[1] $a^{*2} = \boldsymbol{a}^{*\top}\boldsymbol{a}^*$, cf. Eq. 80.

[2] The new modified covariance matrix in the original coordinates is $\boldsymbol{K}^{1/2}\bar{\boldsymbol{C}}'\boldsymbol{K}^{-1/2} = \boldsymbol{K}\bar{\boldsymbol{C}}$, with eigenvectors $\boldsymbol{K}\bar{\boldsymbol{v}}^*$.

[3] We would have to employ the eigenvector basis of the new modified covariance matrix $\bar{\boldsymbol{V}}' = \boldsymbol{K}^{1/2}\bar{\boldsymbol{V}}$ for triangularization.

[4] A change in the overall weight norms, however, can affect the magnitude of postsynaptic activities and synaptic changes.

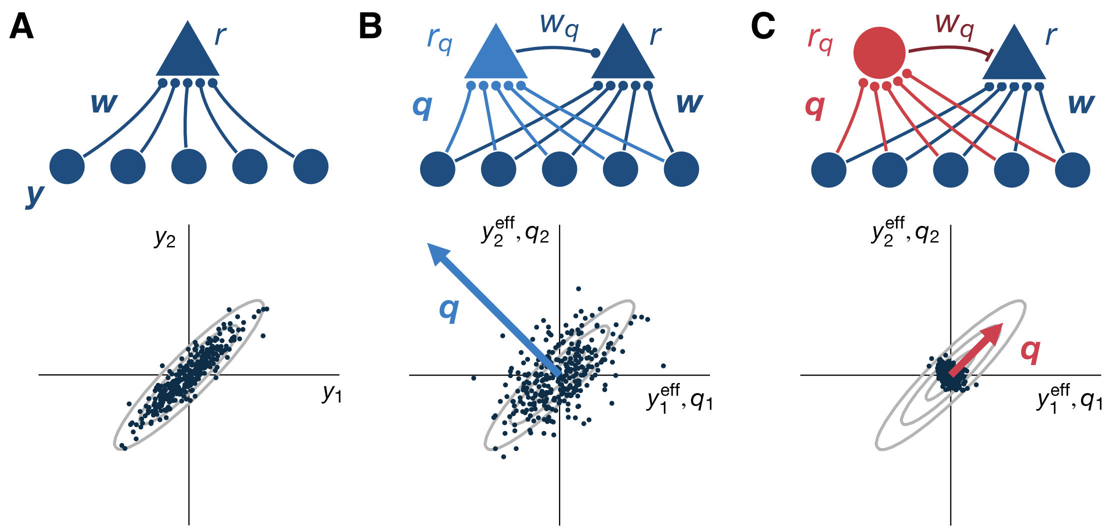

Figure S2: Input space modification due to lateral input. (*A*) Top: a single neuron with firing rate r receives synaptic inputs $\boldsymbol{w}$ from a population of excitatory neurons $\boldsymbol{y}$. Bottom: input distribution projected onto the first two input dimensions. Each dot represents the firing rates of the first two neurons during one input pattern. (Contour lines in light gray). Under a linear Hebbian learning rule, the neuron becomes selective for the direction of maximum variance, the first principal component (cf. Sec. 1). (*B*) Top: Same as in *A* for a neuron that receives additional input w_q from a laterally projecting excitatory neuron r_q which is tuned to an eigenvector $\boldsymbol{q}$ of the original input covariance matrix. Bottom: the effective input space $\boldsymbol{y}^{\text{eff}}$ of the target neuron (dark blue triangle) is warped such that the variance along the eigenvector $\boldsymbol{q}$ (blue arrow) is stretched in proportion to the absolute value of the weight vector $\boldsymbol{q}$. The contour lines of the original input distribution from *A* are shown in light gray for reference. (*C*) Top: Same as *B* for a laterally projecting inhibitory neuron. Bottom: Now, the effective input space is compressed. See text for details.

input space (Fig.1*G*). In the following, we introduce a similar perspective and show that additional lateral input can be interpreted to stretch and compress the original feedforward input space, while the feedforward component of the weight vector performs PCA on this modified input space.

We consider a circuit of two neurons that both receive feedforward input from a population of input neurons $\boldsymbol{y}$ (Fig. S2*B*, top). Let the first neuron have a fixed, non-plastic set of feedforward weights $\boldsymbol{q}$ and firing rate

$$r_q = \boldsymbol{q}^{\mathsf{T}} \boldsymbol{y} \tag{111}$$

We let the first neuron project laterally onto the second neuron via a synaptic weight w_q, without receiving any lateral input itself. Then the equilibrium firing rate of the second neuron is

$$r = w_q r_q + \boldsymbol{w}^{\mathsf{T}} \boldsymbol{y} \tag{112}$$

where we assume that both $\boldsymbol{w}$ and w_q are plastic according to a stabilized Hebbian rule.

From the perspective of the second neuron, the input space is increased by one dimension due to the additional lateral input, i.e., we can write Eq. 112 as

$$r = \bar{\boldsymbol{w}}^{\mathsf{T}} \bar{\boldsymbol{y}}, \qquad \bar{\mathbf{y}} = \left(\mathbf{y}^{\mathsf{T}}, \mathbf{q}^{\mathsf{T}} \mathbf{y}\right)^{\mathsf{T}}, \quad \bar{\boldsymbol{w}} = \left(\boldsymbol{w}^{\mathsf{T}}, w_q\right)^{\mathsf{T}}, \tag{113}$$

where, we defined the new input vector $\bar{\boldsymbol{y}}$ and the combined input weights $\bar{\boldsymbol{w}}$. Effectively this is still a feedforward network without feedback, and the static covariance matrix $\bar{\mathbf{C}}$ of the new inputs $\bar{\mathbf{y}}$ fully determines the average synaptic weight dynamics[1]:

$$\bar{\mathbf{C}} = \left\langle \bar{\mathbf{y}} \bar{\mathbf{y}}^{\mathsf{T}} \right\rangle = \left\langle \begin{pmatrix} \mathbf{y}\mathbf{y}^{\mathsf{T}} & \mathbf{y}\mathbf{y}^{\mathsf{T}}\mathbf{q} \\ \mathbf{q}^{\mathsf{T}}\mathbf{y}\mathbf{y}^{\mathsf{T}} & \mathbf{q}^{\mathsf{T}}\mathbf{y}\mathbf{y}^{\mathsf{T}}\mathbf{q} \end{pmatrix} \right\rangle = \begin{pmatrix} \mathbf{C} & \mathbf{C}\mathbf{q} \\ \mathbf{q}^{\mathsf{T}}\mathbf{C} & \mathbf{q}^{\mathsf{T}}\mathbf{C}\mathbf{q} \end{pmatrix}, \tag{114}$$

where $\boldsymbol{C}$ is the covariance matrix of the original input $\boldsymbol{y}$. We are interested in the eigenvectors $\bar{\boldsymbol{v}}$ and eigenvalues $\bar{\lambda}$ of this matrix for two reasons. First, because they describe the attraction towards different input modes due to the Hebbian term in our competitive plasticity rule. Second, because eigenvectors are fixed points with their stability

[1] In this case, $\bar{\boldsymbol{C}}$ is a true covariance matrix, since the lateral projecting neuron is excitatory.

mostly determined by the eigenvalues. Eigenvectors and eigenvalues must satisfy

$$\bar{\mathbf{C}}\bar{\mathbf{v}} = \bar{\lambda}\bar{\mathbf{v}}, \quad [115]$$

$$\begin{pmatrix} \mathbf{C} & \mathbf{Cq} \\ \mathbf{q}^\top\mathbf{C} & \mathbf{q}^\top\mathbf{Cq} \end{pmatrix}\begin{pmatrix} \mathbf{v}_F \\ v_q \end{pmatrix} = \bar{\lambda}\begin{pmatrix} \mathbf{v}_F \\ v_q \end{pmatrix}. \quad [116]$$

where v_q and $\boldsymbol{v}_F$ are the lateral and the feedforward components of the eigenvector, respectively. In the following, we focus on the feedforward component for different $\boldsymbol{q}$. It follows

$$\mathbf{C}\mathbf{v}_F + \mathbf{Cq}v_q = \bar{\lambda}\mathbf{v}_F, \quad [117]$$

$$\mathbf{q}^\top\mathbf{C}\mathbf{v}_F + \mathbf{q}^\top\mathbf{Cq}v_q = \bar{\lambda}v_q. \quad [118]$$

$$\mathbf{C}\left(\mathbf{v}_F + \mathbf{q}v_q\right) = \bar{\lambda}\mathbf{v}_F, \quad [119]$$

$$\mathbf{q}^\top\mathbf{C}\left(\mathbf{v}_F + \mathbf{q}v_q\right) = \bar{\lambda}v_q. \quad [120]$$

Inserting the first into the second expression gives $v_q = \mathbf{q}^\top\mathbf{v}_F$ which, when inserted into the first expression, results in:

$$\boxed{\mathbf{C}\left(\mathbb{1} + \mathbf{q}\mathbf{q}^\top\right)\mathbf{v}_F = \bar{\lambda}\mathbf{v}_F.} \quad [121]$$

This is again an eigenvector equation, where the feedforward components of the original eigenvector $\boldsymbol{v}_F$, are themselves eigenvectors of the matrix $\mathbf{C}(\mathbb{1} + \mathbf{q}\mathbf{q}^\top)$. Note that for $\boldsymbol{q} = \mathbf{0}$ we recover the case without lateral projections and feedforward components are multiples of eigenvectors of $\boldsymbol{C}$ with attractions $\bar{\lambda} = \lambda$. For general $\boldsymbol{q}$, the solution is not straightforward: We consider the equation in the input eigenspace, where Eq. 121 becomes

$$\Lambda\left(\mathbb{1} + \boldsymbol{q}_v\boldsymbol{q}_v{}^\top\right)\boldsymbol{v}_{Fv} = \bar{\lambda}\boldsymbol{v}_{Fv}, \quad [122]$$

with Λ being the diagonal matrix of feedforward eigenvalues λ and the subscript $(\cdot)_v$ indicates a vector in the eigenbasis of $\boldsymbol{C}$. In this basis, eigenvectors of $\boldsymbol{C}$ are unit vectors, i.e., $\boldsymbol{v}_v = \boldsymbol{e}$, where $\boldsymbol{e}$ is a vector of zeros with a single entry equal to one, corresponding to the respective eigenvector. When $\boldsymbol{q}$ contains components of more than one eigenvector, the matrix $\boldsymbol{q}_v\boldsymbol{q}_v{}^\top$ is not diagonal and eigenvectors of $\boldsymbol{C}$, do not solve the equation. Here we consider a simplified case: When the first neuron had plastic feedforward input, we know from Section 1 that it would converge to a multiple of an eigenvector of the feedforward covariance matrix[1], $\boldsymbol{q} \propto \boldsymbol{v}^\dagger$, with $\boldsymbol{C}\boldsymbol{v}^\dagger = \lambda^\dagger\boldsymbol{v}^\dagger$. Then, $\boldsymbol{q}_v\boldsymbol{q}_v{}^\top = e^\dagger e^{\dagger\top}$ is diagonal with a single non-zero entry, and from Equation 122 it is obvious that feedforward eigenvector components of $\bar{\boldsymbol{C}}$ are eigenvectors of the feedforward covariance matrix $\boldsymbol{C}$ that solve Eq. 121.

To find the eigenvalues $\bar{\lambda}$, we first consider feedforward eigenvector components $\boldsymbol{v}_F$ that are orthogonal to $\boldsymbol{q}$:

$$\boldsymbol{v}_F \propto \boldsymbol{v}^\ddagger \perp \boldsymbol{q} \propto \boldsymbol{v}^\dagger \quad \Rightarrow \quad \boldsymbol{q}^\top\boldsymbol{v}_F \propto \boldsymbol{v}^{\dagger\top}\boldsymbol{v}^\ddagger = 0, \quad [123]$$

Then it follows from Equation 121 that the corresponding eigenvalue of the modified covariance matrix equals the eigenvalue of the original covariance matrix, which is, by definition, equal to the variance $\sigma^{\ddagger 2}$ of the input distribution along the respective eigenvector.

$$\Rightarrow \quad \bar{\lambda}^\ddagger = \lambda^\ddagger = \sigma^{\ddagger 2}. \quad [124]$$

Therefore, input modes that are orthogonal to the tuning of the laterally projecting neuron maintain their attractions, equal to the respective eigenvalues of $\boldsymbol{C}$, and the laterally projecting neuron does not affect the Hebbian growth dynamics in the input subspace orthogonal to $\boldsymbol{q}$[2]. The remaining feedforward eigenvector component is proportional to $\boldsymbol{q}$:

$$\boldsymbol{v}_F \propto \boldsymbol{v}^\dagger \parallel \boldsymbol{q} = a_q\boldsymbol{v}^\dagger \quad \Rightarrow \quad \boldsymbol{q}^\top\boldsymbol{v}_F \propto \boldsymbol{v}^{\dagger\top}\boldsymbol{v}^\dagger = 1, \quad \Rightarrow \quad \bar{\lambda}^\dagger = \lambda^\dagger + \lambda^\dagger a_q^2 = \sigma^{\dagger 2} + \sigma_q^2, \quad [125]$$

[1] More precisely, $\boldsymbol{q}$ would converge to a multiple of the principal eigenvector. Here, we consider the more general case where $\boldsymbol{q}$ is proportional to an arbitrary eigenvector. We will see that with suitable lateral input, any feedforward eigenvector can be stable.

[2] However, the constraint term in the weight dynamics introduces interactions between the subspaces orthogonal and parallel to $\boldsymbol{q}$.

where we again made use of Equation 121. Here, a_q is equal to $\|\boldsymbol{q}\|$, the L2-norm of $\boldsymbol{q}$, and $\sigma_q^2 = \lambda^\dagger a_q^2$ is the firing rate variance of the laterally projecting neuron [1]. Therefore, the second neuron adjusts its feedforward weights $\boldsymbol{w}$ as if the variance along the eigenvector $\boldsymbol{q}$ was increased by σ_q^2 (Fig. S2*B*, bottom). In that sense, the second neuron 'perceives' its feedforward input space as stretched and we speak of a modified input space (cf. Sec. 2.2.1) that is described by a modified covariance matrix $\bar{\boldsymbol{C}}$. We note that it is possible to choose $\boldsymbol{q}$ such that an arbitrary direction of the input space becomes stable. For $\boldsymbol{q} = \boldsymbol{C}^{-1}\boldsymbol{h}$ Eq. 121 is[2]

$$\left(\mathbf{C} + \mathbf{h}\mathbf{h}^\mathsf{T}\boldsymbol{C}^{-1}\right)\mathbf{v}_F = \bar{\lambda}\mathbf{v}_F. \qquad [126]$$

For increasing $\|\boldsymbol{h}\|$, the principal eigenvector transitions from $\boldsymbol{v}_F \propto \boldsymbol{v}$, for $\|\boldsymbol{h}\| = 0$, to $\boldsymbol{v}_F^\infty \propto \boldsymbol{h}$ for $\|\boldsymbol{h}\| \to \infty$. In the following, we only consider the case when $\boldsymbol{q}$ is parallel to one of the eigenvectors of $\boldsymbol{C}$. Then, for sufficiently large a_q and $\boldsymbol{q} \propto \boldsymbol{v}^\dagger$, an arbitrary non-principle eigenvector $\boldsymbol{v}^\dagger$ with attraction $\bar{\lambda}^\dagger = \lambda^\dagger(1 + a_q^2)$ can become stable. In that case, the corresponding fixed point is of the following shape[3]:

$$\Rightarrow \quad \bar{\boldsymbol{w}}^* = \begin{pmatrix} \boldsymbol{w}^* \\ w_q^* \end{pmatrix} = \begin{pmatrix} \boldsymbol{w}^* \\ \boldsymbol{q}^\mathsf{T}\boldsymbol{w}^* \end{pmatrix} \propto \begin{pmatrix} \boldsymbol{v}^\dagger \\ a_q \end{pmatrix}, \qquad [127]$$

When the laterally projecting neuron is inhibitory (Fig. S2*C*, top), the modified covariance matrix becomes (cf. Eq. 51)

$$\bar{\boldsymbol{C}} = \begin{pmatrix} \mathbf{C} & -\mathbf{Cq} \\ \mathbf{q}^\mathsf{T}\mathbf{C} & -\mathbf{q}^\mathsf{T}\mathbf{Cq} \end{pmatrix}, \qquad [128]$$

and it follows that the input space is compressed along $\boldsymbol{q} \propto \boldsymbol{v}^\dagger$ (Fig. S2*C*, bottom):

$$\bar{\lambda}^\dagger = \lambda^\dagger - \lambda^\dagger a_q^2 = \sigma^{\dagger 2} - \sigma_q^2. \qquad [129]$$

In the case of lateral inhibition and sufficiently large vector norms a_q, an eigenvector can become repulsive, i.e., its eigenvalue becomes negative. Geometrically, this corresponds to a reflection of the input space along $\boldsymbol{q}$ through the origin, which can no longer be visualized as intuitively as in Fig. S2.

We can generalize the overall approach to multiple excitatory and inhibitory neurons such that the effective attraction towards a feedforward eigenvector becomes

$$\bar{\lambda} = \lambda\left(1 + \|\boldsymbol{a}_E\|^2 - \|\boldsymbol{a}_I\|^2\right), \qquad [130]$$

$$\Rightarrow \quad \boxed{\bar{\lambda} = \sigma^2 + \|\boldsymbol{\sigma}_E\|^2 - \|\boldsymbol{\sigma}_I\|^2}, \qquad [131]$$

where $\lambda = \sigma^2$, the vectors $\boldsymbol{a}_E$, $\boldsymbol{a}_I$ hold the feedforward vector norms of the laterally projecting neurons that are tuned to the respective feedforward eigenvector, and $\boldsymbol{\sigma}_A = \sqrt{\lambda}\boldsymbol{a}_A$, $A \in \{E, I\}$, hold the standard deviations of their firing rates. This allows writing the regular fixed points as[4]

$$\boxed{\bar{\boldsymbol{w}}^* = \begin{pmatrix} \boldsymbol{w}^* \\ \boldsymbol{w}_E^* \\ \boldsymbol{w}_I^* \end{pmatrix} \propto \begin{pmatrix} \boldsymbol{v} \\ \boldsymbol{a}_E \\ \boldsymbol{a}_I \end{pmatrix} = \sigma^{-1}\begin{pmatrix} \sigma\boldsymbol{v} \\ \boldsymbol{\sigma}_E \\ \boldsymbol{\sigma}_I \end{pmatrix}}, \qquad [132]$$

This implies that for regular fixed points, the total synaptic weight is distributed among lateral synapses in proportion to the standard deviation of their pre-synaptic activities. Note that the different weight norms of the excitatory and inhibitory part of non-eigenvector fixed points can distort this relation (cf. Sec. 2.1.3).

In summary, we demonstrated how static lateral input can be interpreted to reshape the feedforward attraction landscape of afferent neurons. Note that these results are independent of what causes the laterally projecting neurons' tuning. The second, afferent neuron does not 'see' what inputs to the laterally projecting neurons cause their

[1] From Equation 111 we immediately find $\sigma_q^2 = \langle r_q^2 \rangle - \langle r_q \rangle^2 = \mathbf{q}^\mathsf{T}\mathbf{C}\mathbf{q} = \lambda^\dagger a_q^2$, for $\boldsymbol{q} = a_q\boldsymbol{v}^\dagger$, where we assumed zero mean input, $\langle \boldsymbol{y} \rangle = \mathbf{0}$.

[2] Note that $\boldsymbol{C}^{-1} = (\boldsymbol{C}^{-1})^\mathsf{T}$ since $\boldsymbol{C}$ is a true covariance matrix, i.e., $\boldsymbol{C}$ and $\boldsymbol{C}^{-1}$ are symmetric.

[3] If a_q is too small so that $\bar{\lambda}^\dagger < \bar{\lambda}^\ddagger = \lambda^\ddagger$, the principal feedforward eigenvector $\boldsymbol{v}^\ddagger$ of $\boldsymbol{C}$ with eigenvalue $\lambda^\ddagger$ is stable and $\bar{w}^* = (\boldsymbol{v}^{\ddagger\mathsf{T}}, 0)^\mathsf{T}$.

[4] If none of the laterally projecting neurons is tuned to a specific feedforward eigenvector $\boldsymbol{v}^\ddagger$, i.e., $\boldsymbol{v}^\ddagger \perp \boldsymbol{q}_i \ \forall i$, the corresponding fixed point becomes $\bar{v}^\ddagger = (\boldsymbol{v}^{\ddagger\mathsf{T}}, \mathbf{0}^\mathsf{T}, \mathbf{0}^\mathsf{T})^\mathsf{T}$.

tuning. For example, in addition to feedforward input, laterally projecting neurons might be integrated into a recurrent circuit of neurons that are all tuned to the same eigenvector[1]. Then $\boldsymbol{\sigma}_E^2, \boldsymbol{\sigma}_I^2$ result from recurrent interaction in addition to the norm of the feedforward weight vectors. However, for the dynamics of the second neuron, it would not make any difference as long as the firing rate statistics of its pre-synaptic inputs were the same. In the following sections, we will consider circuits where the firing rate statistics emerge from recurrent interactions.

4 Eigencircuits

In the previous section we considered neurons that receive feedforward input from an excitatory population and lateral input from neurons with fixed feedforward tuning (Fig. S2). We found that the attraction of different feedforward input modes is determined by the eigenvalues of a modified covariance matrix, composed of a feedforward contribution and a contribution due to the laterally projecting neurons that is proportional to the variance of their firing rates (Eq. 131). In this section, we consider networks of recurrently connected, laterally projecting neurons and explore the variances of their firing rates.

First, we consider a network of excitatory and inhibitory neurons $\boldsymbol{y}_E, \boldsymbol{y}_I$ that are laterally connected to themselves and each other and receive feedforward input from the same excitatory population $\boldsymbol{y}$. We assume that the activity in the network is dominated by feedforward input such that neurons become selective for different eigenvectors of the feedforward covariance matrix $\boldsymbol{C} = \langle \boldsymbol{y}\boldsymbol{y}^\mathsf{T} \rangle$, e.g., the steady state firing rate y_a of a neuron that is tuned to an eigenvector $\boldsymbol{v}_a$ is proportional to ${\boldsymbol{v}_a}^\mathsf{T}\boldsymbol{y}$ (Fig. 4*A*), where the proportionality factor depends on the number and firing rates of other neurons that are tuned to the same eigenvector (see Sec. 4.1). Then the average Hebbian growth of a synapse that connects two neurons that are tuned to different eigenvectors is[2]:

$$\langle \dot{w}_{ab} \rangle \propto \langle y_a y_b \rangle \propto \langle {\boldsymbol{v}_a}^\mathsf{T} \boldsymbol{y}\boldsymbol{y}^\mathsf{T} \boldsymbol{v}_b \rangle = {\boldsymbol{v}_a}^\mathsf{T} \boldsymbol{C} \boldsymbol{v}_b = \lambda_b {\boldsymbol{v}_a}^\mathsf{T} \boldsymbol{v}_b = 0. \quad [133]$$

Due to the competition for synaptic resources, the synapse loses out to the non-zero growth of synapses that connect neurons that are tuned to the same eigenvector, and decays to zero over time (Fig. 4*B*). Eventually, the circuit is separated into sub-circuits that are tuned to different eigenvectors with recurrent connections within, but not between sub-circuits. Since there is one sub-circuit per eigenvector of the feedforward covariance matrix, we call these decoupled sub-circuits 'eigencircuits' (cf. Fig. 4).

4.1 Variance propagation

In Section 3, we have seen that the attraction and the stability of a feedforward eigenvector are closely related to the firing rate variances of laterally projecting neurons, independent from how these variances arise. In the effective feedforward circuits that we considered, it was straightforward to compute variances based on feedforward weight norms (Eq.131 f.). We now show how variances can be determined in recurrent eigencircuits, which allows to quantify the effective attraction of an input mode.

We consider a generic eigencircuit and investigate how variances propagate through the network, i.e., our goal is to express the standard deviation of a neuron's firing rate as a function of the standard deviations of its pre-synaptic input firing rates. For a neuron in an eigencircuit, all pre-synaptic inputs with non-zero synaptic weight are tuned to the same feedforward eigenvector $\boldsymbol{v}$. We only consider these non-zero entries and assume that the steady state firing rate of an arbitrary neuron can be written as (Fig. S3*A*)

$$r = \boldsymbol{w}^\mathsf{T}\boldsymbol{y} + {\boldsymbol{w}_E}^\mathsf{T}\boldsymbol{y}_E - {\boldsymbol{w}_I}^\mathsf{T}\boldsymbol{y}_I, \quad [134]$$

$$\boldsymbol{y}_E = \boldsymbol{a}_E(\boldsymbol{v}^\mathsf{T}\boldsymbol{y}), \quad \boldsymbol{y}_I = \boldsymbol{a}_I(\boldsymbol{v}^\mathsf{T}\boldsymbol{y}), \quad [135]$$

Note that before, $\boldsymbol{a}_E$ and $\boldsymbol{a}_I$ referred to feedforward weight norms (cf. Sec. 3). Now these vectors more generally express how firing rate variances relate to the input variance along the eigencircuit's feedforward eigenvector, without making any assumptions about how this tuning arises. We will show in Section 5 that this assumption is correct and specify how the entries of $\boldsymbol{a}_E$, $\boldsymbol{a}_I$ relate to the recurrent excitatory and inhibitory weights (cf. Eqs. 161 & 162). For

[1]Another example is neurons that project from outside the local circuit, e.g., from another brain area that is higher up in the processing hierarchy.

[2]Since we assume Hebbian plasticity between all types of neurons, excitatory and inhibitory, we do not specify the neuron type. y_a and y_b are the firing rates of two arbitrary vectors that are part of two different eigencircuits.

the weight vectors, we require that the excitatory and inhibitory parts are normalized to maintain the total amount of inhibitory and excitatory synaptic resources:

$$\begin{pmatrix} \boldsymbol{w} \\ \boldsymbol{w}_E \end{pmatrix} = W_E \frac{\boldsymbol{v}_E}{\|\boldsymbol{v}_E\|_p}, \quad \boldsymbol{w}_I = W_I \frac{\boldsymbol{v}_I}{||\boldsymbol{v}_I||_p}, \tag{136}$$

$$\boldsymbol{v}_E = \begin{pmatrix} \boldsymbol{v} \\ \boldsymbol{a}_E \end{pmatrix}, \quad \boldsymbol{v}_I = \boldsymbol{a}_I, \tag{137}$$

where W_E, W_I are scalar weight norms, and $\boldsymbol{v}_E$, $\boldsymbol{v}_I$ are the excitatory and inhibitory parts of the fixed point eigenvector (cf. Sec. 5), with entries that are proportional to the pre-synaptic standard deviations (cf. Eq. 132). Then, the p-norm, $\|\cdot\|_p$, is maintained due to competition for synaptic resources[1]. For the post-synaptic firing rate, it follows

$$r = \left(\frac{1 + \|\boldsymbol{a}_E\|^2}{\|\boldsymbol{v}_E\|_p} W_E - \frac{\|\boldsymbol{a}_I\|^2}{\|\boldsymbol{v}_I\|_p} W_I \right) (\boldsymbol{v}^\top \boldsymbol{y}). \tag{138}$$

The first bracket is a scalar pre-factor which makes it straightforward to compute the standard deviation:

$$\sigma_r = \left(\frac{1 + \|\boldsymbol{a}_E\|^2}{\|\boldsymbol{v}_E\|_p} W_E - \frac{\|\boldsymbol{a}_I\|^2}{\|\boldsymbol{v}_I\|_p} W_I \right) \sigma = \frac{\sigma^2 + \|\boldsymbol{a}_E\|^2 \sigma^2}{\|\boldsymbol{v}_E\|_p \sigma} W_E - \frac{\|\boldsymbol{a}_I\|^2 \sigma^2}{\|\boldsymbol{v}_I\|_p \sigma} W_I, \tag{139}$$

$$\Rightarrow \quad \boxed{\sigma_r = \frac{\|\boldsymbol{\sigma}^E\|^2}{\|\boldsymbol{\sigma}^E\|_p} W_E - \frac{\|\boldsymbol{\sigma}^I\|^2}{\|\boldsymbol{\sigma}^I\|_p} W_I}, \tag{140}$$

$$\boldsymbol{\sigma}^E = \left(\sigma, \boldsymbol{\sigma}_E{}^\top \right)^\top, \quad \boldsymbol{\sigma}^I = \boldsymbol{\sigma}_I, \tag{141}$$

For a network in the steady state, i.e., when synaptic weights converged, this equation puts the standard deviation of neural firing rates in relation to each other, i.e., it provides the standard deviation of a post-synaptic neuron's activity as a function of the standard deviations of its pre-synaptic input neurons' activities[2]. It describes how standard deviations and variances 'propagate' through the network. In the next section, we will use this variance propagation equation (Eq. 140) to express the standard deviations in terms of only the weight norms and the feedforward standard deviation σ.

4.2 Consistency conditions provide eigencircuit firing rate variances

We now consider a single eigencircuit where n_E excitatory and n_I inhibitory neurons are recurrently connected, and are tuned to the same feedforward eigenvector with standard deviation σ (Fig. S3*B*). In their steady state, all neurons have to fulfil the variance propagation equation (Eq. 140). In the fully connected eigencircuit, the firing rate variance of each neuron depends on the firing rate variances of all other neurons, and all neurons have the same set of non-zero pre-synaptic inputs. This provides $N = n_E + n_I$ consistency conditions for the N unknown standard deviations. For example, the condition for a single excitatory neuron i reads

$$\sigma_E^i = W_{EE}^i \left(\frac{\sigma^2 + \|\boldsymbol{\sigma}_E\|^2}{\sigma + \|\boldsymbol{\sigma}_E\|_1} \right) - W_{EI}^i \left(\frac{\|\boldsymbol{\sigma}_I\|^2}{\|\boldsymbol{\sigma}_I\|_1} \right), \tag{142}$$

where we chose the L1-norm, $p = 1$, for normalization (but see Sec. 4.3), and $W_{AB},\ A, B \in \{E, I\}$ are the total synaptic weight that a neuron of type A receives from neurons of type B. We make the simplifying assumption that all neurons have similar weight norms, i.e., $W_{AB}^i \approx W_{AB},\ \forall i,\ A, B \in \{E, I\}$. Then, also the standard deviations of their activities are similar, and we approximate $\sigma_A^i \approx \sigma_A,\ \forall i,\ A \in \{E, I\}$:

$$\|\boldsymbol{\sigma}_A\|^2 = \sum_i \sigma_A^{i2} \approx n_A \sigma_A^2, \quad \Rightarrow \quad \frac{\sigma^2 + \|\boldsymbol{\sigma}_A\|^2}{\sigma + \|\boldsymbol{\sigma}_A\|_1} \approx \frac{\sigma^2 + n_A \sigma_A^2}{\sigma + n_A \sigma_A}. \tag{143}$$

[1]The vectors $(\boldsymbol{w}^\top, \boldsymbol{w}_E{}^\top)^\top$ and $\boldsymbol{w}_I$ are normalized such that $\left\|(\boldsymbol{w}^\top, \boldsymbol{w}_E{}^\top)^\top\right\|_p = W_E$, and $\|\boldsymbol{w}_I\|_p = W_I$. This is achieved by scaling the excitatory and inhibitory part of the regular eigenvector, i.e., scaling $\boldsymbol{v}_E$ by $k_E = W_E / \|\boldsymbol{v}_E\|_p$, and $\boldsymbol{v}_I$ by $k_I = W_I / \|\boldsymbol{v}_I\|_p$ (cf. Sec. 2.1.3)

[2]Note that we allow self-excitation and self-inhibition, i.e., in a fully connected recurrent network, σ_r also appears on the right sides of the equation, as an entry of $\boldsymbol{\sigma}^E$ or $\boldsymbol{\sigma}^I$.

The standard deviations of excitatory and inhibitory neural firing rates become

$$\sigma_E = W_{EE}\left(\frac{\sigma^2 + n_E\sigma_E^2}{\sigma + n_E\sigma_E}\right) - W_{EI}\left(\frac{n_I\sigma_I^2}{n_I\sigma_I}\right), \qquad \sigma_I = W_{IE}\left(\frac{\sigma^2 + n_E\sigma_E^2}{\sigma + n_E\sigma_E}\right) - W_{II}\left(\frac{n_I\sigma_I^2}{n_I\sigma_I}\right). \tag{144}$$

After some algebra, this yields the standard deviations as

$$\boxed{\sigma_I = \frac{W_{IE}}{1 + W_{II}}\frac{1}{\Phi}\sigma_E}, \qquad \Phi \equiv \left[W_{EE} - \frac{W_{EI}W_{IE}}{1 + W_{II}}\right], \tag{145}$$

$$\Rightarrow \quad \boxed{\sigma_E = \frac{1}{2(1-\Phi)\,n_E}\left(-1 \pm \sqrt{1 + 4\Phi(1-\Phi)\,n_E}\right)\sigma}. \tag{146}$$

This provides standard deviations as a function of the number of neurons in the eigencircuit[1], n_E, n_I, their weight norms, W_{AB}, and the standard deviation of the feedforward input activity along the corresponding eigenvector, σ. Via Eq. 131, we can determine how the eigencircuit modifies the attraction of its feedforward eigenvector, i.e., the effective attraction $\bar{\lambda}$ is

$$\bar{\lambda} = \sigma^2 + n_E\sigma_E^2 - n_I\sigma_I^2 \equiv \lambda + \lambda_{\text{eig}}, \tag{147}$$

where we defined the attraction of the eigencircuit, λ_{eig}, and λ is the attraction of the respective feedforward eigenvector. In the following, we refer to $\bar{\lambda}$ interchangeably as the effective attraction of the eigencircuit or the effective attraction of the feedforward input mode.

In summary, we assumed that neurons are tuned to feedforward eigenvectors (Eq.135) and showed how the network decomposes into recurrent eigencircuits. We demonstrated how variances propagate through such eigencircuits, and quantified how eigencircuits modify the attraction of their feedforward eigenvector (cf. Sec. 3) by laterally projecting onto other neurons (cf. Fig. 4*C*). In the following (Sec. 5), we will show that eigencircuits are indeed stable fixed points of fully plastic recurrent networks and investigate their stability.

4.3 A note on the choice of weight norm

The choice of the weight norm that is maintained via multiplicative normalization is non-trivial. Biologically we motivated normalization by the competition for a limited amount of synaptic resources. We assumed the simplest case, where the L1-norm is maintained, and each resource unit translates to one unit of synaptic strength. An alternative choice would be to maintain the L2-norm. In the variance propagation equation (Eq. 140) this corresponds to $p = 2$ which becomes

$$\sigma_r = \left\|\boldsymbol{\sigma}^E\right\|W_E - \left\|\boldsymbol{\sigma}^I\right\|W_I. \tag{148}$$

Following a similar logic as in Section 4.2, the eigencircuit consistency condition for a single inhibitory neuron becomes (cf. Eq. 142):

$$\sigma_I = \frac{W_{IE}}{1 + W_{II}}\left(\sigma^2 + \|\boldsymbol{\sigma}_E\|^2\right)^{\frac{1}{2}}, \tag{149}$$

where we once more assumed that all neurons have similar weight norms, $W_{AB}^i \approx W_{AB}, \forall i$. The variance of an excitatory neuron becomes

$$\sigma_E^2 = \Phi^2\left(\sigma^2 + \|\boldsymbol{\sigma}_E\|^2\right) = \Phi^2\left(\sigma^2 + n_E\sigma_E^2\right), \tag{150}$$

$$\Rightarrow \quad \boxed{\sigma_E^2 = \frac{\Phi^2}{1 - \Phi^2 n_E}\sigma^2}. \tag{151}$$

For an increasing number of excitatory neurons n_E, the firing rate variance of a single excitatory neuron grows and diverges for $\Phi^2 n_E = 1$. For even larger n_E, variances would have to be negative to fulfil the consistency condition,

[1] Note that for $0 > \Phi < 1$ there exists a real solution for σ_E, independent of n_E.

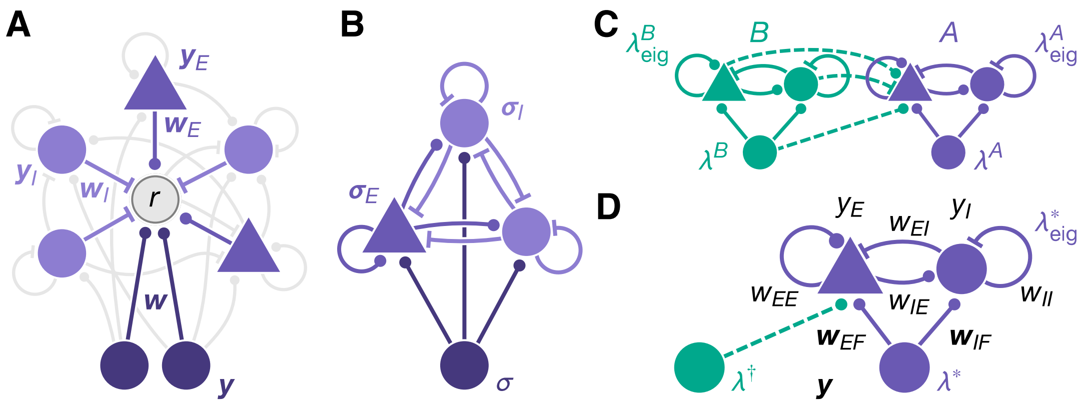

Figure S3: (*A*) A neuron with firing rate r (gray, center) receives synaptic inputs as part of a recurrent eigencircuit. The neuron receives excitatory synapses $\boldsymbol{w}$ from a population of input neurons $\boldsymbol{y}$ (dark purple, bottom). Excitatory (purple, triangles) and inhibitory neurons (light purple, circles) with firing rates, $\boldsymbol{y_E}, \boldsymbol{y}_I$, that are part of the same eigencircuit, project laterally onto neuron r via excitatory $\boldsymbol{w}_E$ and inhibitory $\boldsymbol{w}_I$ synapses. Recurrent synaptic connections that are not inputs of neuron r are shown in light gray – Not all synaptic connections are shown, for clarity. (*B*) Recurrently connected eigencircuit of $n_E = 1$ excitatory neuron (purple triangle) and $n_I = 2$ inhibitory neurons (light purple circles) that are tuned to the same feedforward eigenvector (dark purple circle, bottom). The standard deviation σ of input firing rates along the input eigenvector propagates through the network and results in firing rate standard deviations of $\boldsymbol{\sigma}_E$ and $\boldsymbol{\sigma}_I$ (cf. Eq. 145 f.). (*C*) Two excitatory neurons (triangles, top) and two inhibitory neurons (circles, top) in a recurrent circuit receive feedforward excitation from two input neurons (purple and green circles, bottom) that correspond to two different eigenvectors with eigenvalue λ^A, λ^B. Neurons are configured in a fixed point with two eigencircuits, A and B, with eigencircuit attractions λ^A_{eig}, λ^B_{eig} (cf. Eq. 147). Neurons that are part of the same eigencircuit are recurrently connected to each other. Synaptic weights between neurons that are tuned to different eigencircuits are zero. The excitatory neuron in eigencircuit A is perturbed in the direction of eigencircuit B (dashed lines). (*D*) Equivalent circuit with one excitatory and one inhibitory neuron. We consider a fixed point, where both neurons are tuned to the same feedforward eigenvector with eigenvalue λ^*. The neurons form an eigencircuit with attraction λ^*_{eig}. The excitatory neuron is perturbed in the direction of another feedforward eigenvector with attraction $\lambda^\dagger$ (dashed line). Firing rates, $y_E, y_I, \boldsymbol{y}$, and recurrent and feedforward synaptic weights, $w_{EE}, w_{EI}, w_{IE}, w_{II}, \boldsymbol{w}_{EF}, \boldsymbol{w}_{IF}$, are shown in black (cf. Eqs.153 & 153). See text for details.

which is not possible. It follows that for sufficiently large n_E there exist no fixed points. This is not unique to the L2-norm but holds for any $p > 1$. Such norms allow for a larger total synaptic weight (in terms of its L1-norm) when distributed across multiple synapses. Additional neurons provide additional recurrent synapses, which leads to the growth of the effective recurrent excitation until activities can no longer be stabilized by recurrent inhibition. For a suitable choice of the weight norms, Φ can, in principle, become small enough to balance the number of excitatory neurons in any eigencircuit to maintain positive variances. However, this requires additional fine-tuning and fails when n_E becomes unexpectedly large.

5 E-I networks with fully plastic recurrent connectivity

We now consider fully connected networks of excitatory and inhibitory neurons where all connections, feedforward and recurrent, are plastic according to the competitive Hebbian learning rule we introduced in Section 2. We will first show that eigencircuits are fixed points and then consider their stability with respect to a weight perturbation. Specifically, we would like to know when a neuron from one eigencircuit becomes attracted to a different feedforward eigenvector. We start with some simplifying assumptions.

Since each neuron can be bidirectionally connected to all other neurons, the dimensionality of the weight dynamics grows quadratically with the number of neurons. We are only interested in the general principles and consider a simplified circuit of two excitatory and two inhibitory neurons. One possible fixed point configuration is shown in Figure S3*C* (without dashed lines), where neurons are configured in two eigencircuits, A,B, with one excitatory and one inhibitory neuron per eigencircuit[1]. In this fixed point, all neurons receive feedforward input from a population of input neurons but synapses that connect neurons of different eigencircuits are zero (cf. Sec. 4). When a neuron in eigencircuit A is perturbed towards the other eigencircuit B (Fig. S3*C*, dashed lines), the tuning and the firing rates of all neurons in eigencircuit A change. However, neurons in eigencircuit B are unaffected because there are no connections projecting from eigencircuit A to eigencircuit B. Therefore, we only consider the recurrence within eigencircuit A, and think of input from other eigencircuits as effectively feedforward and static: That is, we construct an equivalent circuit where we perturb an excitatory neuron that is part of an eigencircuit, '$*$', in the direction of another eigencircuit, '$\dagger$', that does not contain any neurons and has feedforward attraction equal to the effective

[1]We will show in Section 5.1 that eigencircuits are in fact fixed points of the weight dynamics.

attraction of eigencircuit B, that is[1] (Fig. S3*D*)

$$\lambda^{\dagger} = \bar{\lambda}^{B} = \lambda^{B} + \lambda^{B}_{\text{eig}}, \quad \lambda^{\dagger}_{\text{eig}} = 0. \tag{152}$$

The configuration and attraction of the perturbed eigencircuit '*' is equal to eigencircuit A, i.e., $\lambda^{*} = \lambda^{A}$, $\lambda^{*}_{\text{eig}} = \lambda^{A}_{\text{eig}}$. In Section 5.2.3 we will explain in more detail why these two circuits (Fig. S3*C* & *D*) are highly similar with regards to their stability.

In the equivalent circuit (Fig. S3*D*), we now consider the generic equilibrium firing rates of the $n_E = 1$ excitatory and $n_I = 1$ inhibitory neuron without taking any tuning into account (Fig. S3*D*)

$$y_E = \boldsymbol{w}_{EF}{}^{\mathsf{T}}\boldsymbol{y} + w_{EE}y_E - w_{EI}y_I, \tag{153}$$

$$y_I = \boldsymbol{w}_{IF}{}^{\mathsf{T}}\boldsymbol{y} + w_{IE}y_E - w_{II}y_I, \tag{154}$$

where $\boldsymbol{y}$ holds the firing rates of a population of N_F input neurons and we did not assume any specific tuning of the feedforward weights $\boldsymbol{w}_{EF}$, $\boldsymbol{w}_{IF}$. Since the network is linear, we can solve for the firing rates:

$$y_E = \frac{1}{1 - w_{EE} + \dfrac{w_{EI}w_{IE}}{1 + w_{II}}}\left(\boldsymbol{w}_{EF}{}^{\mathsf{T}} - \frac{w_{EI}\boldsymbol{w}_{IF}{}^{\mathsf{T}}}{1 + w_{II}}\right)\boldsymbol{y} \equiv \boldsymbol{a}_E{}^{\mathsf{T}}\boldsymbol{y}, \tag{155}$$

$$y_I = \frac{1}{1 + w_{II} + \dfrac{w_{IE}w_{EI}}{1 - w_{EE}}}\left(\boldsymbol{w}_{IF}{}^{\mathsf{T}} + \frac{w_{IE}\boldsymbol{w}_{EF}{}^{\mathsf{T}}}{1 - w_{EE}}\right)\boldsymbol{y} \equiv \boldsymbol{a}_I{}^{\mathsf{T}}\boldsymbol{y}, \tag{156}$$

where we defined the effective feedforward vectors $\boldsymbol{a}_E$, $\boldsymbol{a}_I$. The weight dynamics is

$$\boldsymbol{\tau}\dot{\boldsymbol{w}} = \begin{pmatrix} \dot{\boldsymbol{w}}_{EF} \\ \dot{w}_{EE} \\ \dot{w}_{EI} \\ \vdots \end{pmatrix} = \begin{pmatrix} \boldsymbol{y}\boldsymbol{y}^{\mathsf{T}} & \boldsymbol{y}y_E & -\boldsymbol{y}y_I & \\ y_E\boldsymbol{y}^{\mathsf{T}} & y_Ey_E & -y_Ey_I & \mathbf{0} \\ y_I\boldsymbol{y}^{\mathsf{T}} & y_Iy_E & -y_Iy_I & \\ & \mathbf{0} & & \ddots \end{pmatrix}\begin{pmatrix} \boldsymbol{w}_{\boldsymbol{EF}} \\ w_{EE} \\ w_{EI} \\ \vdots \end{pmatrix} - \begin{pmatrix} \gamma_E & 0 & 0 & \\ 0 & \gamma_E & 0 & \mathbf{0} \\ 0 & 0 & \rho_E & \\ & \mathbf{0} & & \ddots \end{pmatrix}\begin{pmatrix} \boldsymbol{w}_{\boldsymbol{EF}} \\ w_{EE} \\ w_{EI} \\ \vdots \end{pmatrix}, \tag{157}$$

where ellipsis indicate similar terms for afferent weights of the inhibitory neuron. We define the modified covariance matrix

$$\bar{\boldsymbol{C}} = \begin{pmatrix} \langle\boldsymbol{y}\boldsymbol{y}^{\mathsf{T}}\rangle & \langle\boldsymbol{y}y_E\rangle & -\langle\boldsymbol{y}y_I\rangle & \\ \langle y_E\boldsymbol{y}^{\mathsf{T}}\rangle & \langle y_Ey_E\rangle & -\langle y_Ey_I\rangle & \mathbf{0} \\ \langle y_I\boldsymbol{y}^{\mathsf{T}}\rangle & \langle y_Iy_E\rangle & -\langle y_Iy_I\rangle & \\ & \mathbf{0} & & \ddots \end{pmatrix} = \begin{pmatrix} \boldsymbol{C} & \boldsymbol{C}\boldsymbol{a}_E & -\boldsymbol{C}\boldsymbol{a}_I & \\ \boldsymbol{a}_E{}^{\mathsf{T}}\boldsymbol{C} & \boldsymbol{a}_E{}^{\mathsf{T}}\boldsymbol{C}\boldsymbol{a}_E & -\boldsymbol{a}_E{}^{\mathsf{T}}\boldsymbol{C}\boldsymbol{a}_I & \mathbf{0} \\ \boldsymbol{a}_I{}^{\mathsf{T}}\boldsymbol{C} & \boldsymbol{a}_I{}^{\mathsf{T}}\boldsymbol{C}\boldsymbol{a}_E & -\boldsymbol{a}_I{}^{\mathsf{T}}\boldsymbol{C}\boldsymbol{a}_I & \\ & \mathbf{0} & & \ddots \end{pmatrix}, \tag{158}$$

and write the average synaptic change as[2] (cf. Eq. 40)

$$\Rightarrow \quad \bar{\boldsymbol{\tau}}\dot{\bar{\boldsymbol{w}}} \equiv \bar{\boldsymbol{C}}\bar{\boldsymbol{w}} - \boldsymbol{\Gamma}\bar{\boldsymbol{w}}, \tag{159}$$

where $\boldsymbol{\Gamma}$ is a diagonal matrix that holds the scalar constraint factors, and $\bar{\boldsymbol{\tau}}$ holds the timescales for excitatory synapses, $\boldsymbol{\tau}_E = \mathbb{1}\tau_E$, and inhibitory synapses, $\boldsymbol{\tau}_I = \mathbb{1}\tau_I$, on the diagonal. We make the simplifying assumption that the plasticity of excitatory and inhibitory synapses is equally fast, $\tau_E = \tau_I = \tau$. Then $\bar{\boldsymbol{\tau}} = \tau\mathbb{1}$, which does not affect the fixed points or the stability of the system[3], and we set $\bar{\boldsymbol{\tau}} = \mathbb{1}$.

Note that this is a highly non-linear dynamical system since the modified covariance matrix not only depends on the feedforward inputs $\boldsymbol{y}$ but also on the plastic synaptic weights $\bar{\boldsymbol{w}}$, in addition to the weight dependence of the normalization factors $\boldsymbol{\Gamma}$. Next, we show that the eigencircuit configuration we discussed in the introduction to this section is in fact a fixed point of the weight dynamics.

[1] See Eq. 147 for the definition of the eigencircuit attraction λ_{eig}.
[2] We omitted the angle notation $\langle\cdot\rangle$ to improve readability.
[3] It does not affect the sign of the eigenvalues of the Jacobian, since τ is always positive. In principle, however, different timescales for excitatory and inhibitory weights can affect stability (cf. Sec. 2.2).

5.1 Fixed points

In general, fixed points $\bar{\boldsymbol{w}}^*$ must fulfil the following condition

$$\bar{\mathbf{C}}^*\bar{\boldsymbol{w}}^* - \boldsymbol{\Gamma}^*\bar{\boldsymbol{w}}^* \overset{!}{=} 0. \quad [160]$$

where $\bar{\boldsymbol{C}}^*$ is the modified covariance matrix evaluated in the fixed point. We consider the special case when the two neurons form a single eigencircuit, tuned to the feedforward eigenvector $\boldsymbol{v}^*$. Then we can write the excitatory and inhibitory firing rates as[1]

$$y_E^* = \boldsymbol{a}_E^{*\mathsf{T}}\boldsymbol{y} = \boldsymbol{y}^\mathsf{T}\boldsymbol{a}_E^*, \quad \boldsymbol{a}_E^* = a_E^*\boldsymbol{v}^*, \quad [161]$$

$$y_I^* = \boldsymbol{a}_I^{*\mathsf{T}}\boldsymbol{y} = \boldsymbol{y}^\mathsf{T}\boldsymbol{a}_I^*, \quad \boldsymbol{a}_I^* = a_I^*\boldsymbol{v}^*, \quad [162]$$

where a_E and a_I depend on the excitatory and inhibitory weights and can be determined via Eq. 155 & 156. This demonstrates that when neurons are tuned to the same feedforward eigenvector $\boldsymbol{v}^*$, their firing rate is proportional to the projection of the activity vector $\boldsymbol{y}$ onto the eigenvector $\boldsymbol{v}^*$, and justifies our assumption in Eq. 135. The modified covariance matrix in the fixed point becomes

$$\bar{\boldsymbol{C}}^* = \begin{pmatrix} \boldsymbol{C} & \boldsymbol{C}\boldsymbol{v}^*a_E^* & -\boldsymbol{C}\boldsymbol{v}^*a_I^* & \\ a_E^*\boldsymbol{v}^{*\mathsf{T}}\boldsymbol{C} & a_E^*\boldsymbol{v}^{*\mathsf{T}}\boldsymbol{C}\boldsymbol{v}^*a_E^* & -a_E^*\boldsymbol{v}^{*\mathsf{T}}\boldsymbol{C}\boldsymbol{v}^*a_I^* & \boldsymbol{0} \\ a_I^*\boldsymbol{v}^{*\mathsf{T}}\boldsymbol{C} & a_I^*\boldsymbol{v}^{*\mathsf{T}}\boldsymbol{C}\boldsymbol{v}^*a_E^* & -a_I^*\boldsymbol{v}^{*\mathsf{T}}\boldsymbol{C}\boldsymbol{v}^*a_I^* & \\ & \boldsymbol{0} & & \ddots \end{pmatrix} = \begin{pmatrix} \boldsymbol{C} & \lambda^*a_E^*\boldsymbol{v}^* & -\lambda^*a_I^*\boldsymbol{v}^* & \\ \lambda^*a_E^*\boldsymbol{v}^{*\mathsf{T}} & \lambda^*a_E^{*2} & -\lambda^*a_E^*a_I^* & \boldsymbol{0} \\ \lambda^*a_I^*\boldsymbol{v}^{*\mathsf{T}} & \lambda^*a_I^*a_E^* & -\lambda^*a_I^{*2} & \\ & \boldsymbol{0} & & \ddots \end{pmatrix}. \quad [163]$$

which can be diagonalized by the eigenvector matrix $\bar{\boldsymbol{V}}^*$ and its inverse:

$$\bar{\boldsymbol{V}}^* = \begin{pmatrix} \boldsymbol{V}_{\backslash *} & \boldsymbol{v}^* & \boldsymbol{v}^*a_E^* & \boldsymbol{v}^*a_I^* & \\ \boldsymbol{0} & a_E^* & -1 & 0 & \boldsymbol{0} \\ \boldsymbol{0} & a_I^* & 0 & 1 & \\ & & \boldsymbol{0} & & \ddots \end{pmatrix}, \quad \bar{\boldsymbol{V}}^{*-1} = \mathcal{N}^{-1}\begin{pmatrix} \mathcal{N}\boldsymbol{V}_{\backslash *}^{\mathsf{T}} & \boldsymbol{0} & \boldsymbol{0} & \\ \boldsymbol{v}^{*\mathsf{T}} & a_E^* & -a_I^* & \\ a_E\boldsymbol{v}^{*\mathsf{T}} & -(1-a_I^{*2}) & -a_E^*a_I^* & \boldsymbol{0} \\ -a_I\boldsymbol{v}^{*\mathsf{T}} & -a_I^*a_E^* & 1+a_E^{*2} & \\ & \boldsymbol{0} & & \ddots \end{pmatrix}, \quad [164]$$

$$\mathcal{N} \equiv 1 + a_E^{*2} - a_I^{*2}, \quad [165]$$

where the subscript $(\cdot)_{\backslash *}$ indicates that a matrix does not contain an entry that corresponds to the input mode $\boldsymbol{v}^*$.

In general, $\bar{\boldsymbol{C}}^*$ has one diagonal block of dimension $D = N_F + N_E + N_I$ per neuron in the circuit, i.e., $N_E + N_I$ blocks[2]. Then, $\bar{\boldsymbol{C}}^*$ is of dimension $(N_E + N_I)D \times (N_E + N_I)D$. Therefore, to diagonalize $\bar{\boldsymbol{C}}^*$, we require $(N_F + N_E + N_I)(N_E + N_I)$ eigenvectors. Because $\bar{\boldsymbol{C}}^*$ has a block diagonal structure (Eq. 163), with the first $D \times D$ block driving development of weights onto the excitatory neuron and the second $D \times D$ block driving development of weights onto the inhibitory neuron, the eigenvector matrix $\bar{\boldsymbol{V}}^*$ and its inverse have the same block diagonal structure. Since each block has the same sub-structure, we only show the first block in Eq. 164. Assuming that all neurons in the circuit are tuned to a feedforward eigenvector, we have $N_F + n_E + n_I$ eigenvectors of $\bar{\boldsymbol{C}}^*$ per eigencircuit and block, where n_E and n_I are the number of excitatory and inhibitory neurons in the respective eigencircuit: N_F regular eigenvectors, and $n_E + n_I$ null eigenvectors (cf. Sec. 2.1.2). For the specific circuit at hand, we have one excitatory and one inhibitory neuron, $N_E = N_I = 1$, recurrently connected in the same eigencircuit, i.e., there are $N_F - 1$ eigencircuits with $n_E = n_I = 0$ and one eigencircuit with $n_E^* = n_I^* = 1$. The first column of $\bar{\boldsymbol{V}}^*$ in Eq. 164 corresponds to the $N_F - 1$ eigencircuits with $n_E = n_I = 0$, i.e., with one regular eigenvector per feedforward eigenvector $\boldsymbol{v} \neq \boldsymbol{v}^*$, but without null-eigenvectors. The corresponding eigenvalues are $\boldsymbol{\Lambda}_{\backslash *}$, which are also eigenvalues of the feedforward covariance matrix $\boldsymbol{C}$. For the eigencircuit corresponding to $\boldsymbol{v}^*$ there are $1 + n_E^* + n_I^* = 3$ eigenvectors of $\bar{\boldsymbol{C}}^*$. The first is a regular eigenvector and the last two are null eigenvectors, where the excitatory feedforward component is cancelled by either a negative lateral

[1] Note that here the superscript '*' indicates a variable that is evaluated in the fixed point of the weight dynamics and not a fixed point of the firing rate activity. Different input patterns $\boldsymbol{y}$ result in different neural activities y_E^*, y_I^*.

[2] Remember that N_F is the number of input neurons, and N_E, N_I are the total excitatory and inhibitory neurons in the circuit, respectively.

excitatory component[1], or a positive lateral inhibitory component [2]. The null eigenvectors have eigenvalues equal to zero, and the eigenvalue of the regular eigenvector is $\bar{\lambda}^* = \lambda^*(1 + a_E^{*2} - a_I^{*2})$.

Similar to the feedforward case, arbitrary multiples of the separately normalized parts of eigenvectors of $\bar{\boldsymbol{C}}^*$ are fixed points. The only exception is the rightmost null eigenvector (cf. Sec. 2.1.3). There, the inhibitory and the excitatory weights are aligned such that the post-synaptic activity is zero, which does not allow for arbitrary scaling of the excitatory and inhibitory weight norms. Inserting these fixed points into Equations 155 & 156, provides conditions to determine a_E^* and a_I^*.

5.2 Stability analysis

We are interested in the stability of the circuit described in the introduction of Section 5 and consider the stability of a regular eigenvector $\bar{\boldsymbol{v}}^*$

$$\bar{\boldsymbol{w}}^* = \bar{\boldsymbol{v}}^* = \begin{pmatrix} \boldsymbol{v}^* \\ a_E^* \\ a_I^* \\ \boldsymbol{v}^* \\ a_E^* \\ a_I^* \end{pmatrix}, \quad \bar{\lambda}^* = \lambda^* \left(1 + a_E^{*2} - a_I^{*2}\right), \tag{166}$$

This means we do *not* consider arbitrary scalings of the excitatory and inhibitory parts of eigenvectors of $\bar{\boldsymbol{C}}^*$, but assume that weight norms are fine tuned to match the norms of the excitatory and inhibitory parts of the regular eigenvector[3] $\bar{\boldsymbol{v}}^*$.

When are such eigenvectors stable, and when are they attracted to a different input mode? To answer this question, we consider small fixed point perturbations $\Delta\bar{\boldsymbol{w}}(t_0)$, where the excitatory neuron shifts its tuning in the direction of a different feedforward input eigenvector $\boldsymbol{v}^\dagger$:

$$\Delta\bar{\boldsymbol{w}}(t_0) \propto \begin{pmatrix} \boldsymbol{v}^\dagger \\ 0 \\ 0 \\ \boldsymbol{0} \\ 0 \\ 0 \end{pmatrix} = \bar{\boldsymbol{V}}^* \boldsymbol{e}^\dagger. \tag{167}$$

where $\boldsymbol{e}^\dagger$ is a vector of zeros with a single non-zero entry that corresponds to the feedforward eigenvector $\boldsymbol{v}^\dagger$ (cf. Eq. 164). The system is stable with respect to a perturbation if the perturbation decays to zero over time. To check this, we consider the following differential equation that holds for small perturbations (cf. Sec. 1.2.2)

$$\frac{\mathrm{d}}{\mathrm{d}t}\Delta\bar{\boldsymbol{w}}(t) = \bar{\boldsymbol{J}}^* \Delta\bar{\boldsymbol{w}}(t), \tag{168}$$

where $\bar{\boldsymbol{J}}^*$ is the Jacobian evaluated in the fixed point. We will consider the dynamics in the non-orthogonal eigenbasis $\bar{\boldsymbol{V}}^*$ of the modified covariance matrix $\bar{\boldsymbol{C}}^*$ evaluated in the fixed point $\bar{\boldsymbol{w}}^* = \bar{\boldsymbol{v}}^*$. Note that $\bar{\boldsymbol{V}}^*$ is not time-dependent,

[1] In our simulations, we constrain synaptic weights to be positive. Then null eigenvectors with negative weights are only relevant in combination with regular eigenvectors: When a null eigenvector is added to a regular eigenvector, the net synaptic input does not change. For example, a decrease in recurrent excitation due to a negative excitatory component of the null eigenvector is balanced by an increase in feedforward excitation.

[2] We can generalize this approach to the case where neurons are tuned to different feedforward eigenvectors. For example, consider we add a second excitatory neuron that is, however, tuned to a different feedforward eigenvector, $\boldsymbol{v}^\dagger$. This gives rise to an additional null eigenvector, $(\boldsymbol{v}^{\dagger\top} a_E^\dagger, 0, 0, -1, \boldsymbol{0}^\top)^\top$, in the first block of $\bar{\boldsymbol{V}}^*$ (Eq. 164). In addition, one of the regular eigenvectors in the first column block of $\bar{\boldsymbol{V}}^*$ (Eq. 164) becomes $(\boldsymbol{v}^{\dagger\top}, 0, 0, a_E^\dagger, \boldsymbol{0}^\top)^\top$. Importantly, this is the case for each diagonal block of $\bar{\boldsymbol{V}}^*$, i.e., we get D additional null eigenvectors and D altered regular eigenvectors per additional neuron. This ensures that we always have $N_E + N_I$ null eigenvectors and N_F regular eigenvectors per block, which allows to diagonalize $\bar{\boldsymbol{C}}^*$ which is of dimension $(N_E + N_I)D \times (N_E + N_I)D$, independent from the feedforward tunings of neurons – with the caveat that all neurons must be tuned to feedforward eigenvectors.

[3] We presume that when considering the stability of non-eigenvector fixed points, it is possible to make a similar argument as in Section 2.2.3 and consider regular eigenvectors of a different modified covariance matrix $\bar{\boldsymbol{C}}'$ with adjusted plasticity timescales, $k_E\tau_E$, $k_I\tau_I$. Here we consider the case of $\tau_E = \tau_I$ and regular eigenvectors of $\bar{\boldsymbol{C}}^*$, i.e., $k_E = k_I = 1$.

because it is evaluated in the fixed point. In this static basis, we can express perturbations as

$$\Delta\bar{\boldsymbol{w}}_v(t) = \bar{\boldsymbol{V}}^{*-1}\Delta\bar{\boldsymbol{w}}(t), \tag{169}$$

$$\Rightarrow \Delta\bar{\boldsymbol{w}}_v(t_0) = \bar{\boldsymbol{V}}^{*-1}\Delta\bar{\boldsymbol{w}}(t_0) \propto \boldsymbol{e}^{\dagger}, \tag{170}$$

where the subscript $(\cdot)_v$ indicates a vector or matrix expressed in this basis. The perturbation dynamics becomes

$$\frac{\mathrm{d}}{\mathrm{d}t}\Delta\bar{\boldsymbol{w}}_v(t) = \frac{\mathrm{d}}{\mathrm{d}t}\left(\bar{\boldsymbol{V}}^{*-1}\Delta\bar{\boldsymbol{w}}(t)\right) = \bar{\boldsymbol{V}}^{*-1}\frac{\mathrm{d}}{\mathrm{d}t}\Delta\bar{\boldsymbol{w}}(t) = \bar{\boldsymbol{V}}^{*-1}\bar{\boldsymbol{J}}^{*}\Delta\bar{\boldsymbol{w}}(t) = \bar{\boldsymbol{V}}^{*-1}\bar{\boldsymbol{J}}^{*}\bar{\boldsymbol{V}}^{*}\Delta\bar{\boldsymbol{w}}_v(t) = \bar{\boldsymbol{J}}^{*}_v\,\Delta\bar{\boldsymbol{w}}_v(t), \tag{171}$$

where we defined the transformed Jacobian, $\bar{\boldsymbol{J}}^{*}_v = \bar{\boldsymbol{V}}^{*-1}\bar{\boldsymbol{J}}^{*}\bar{\boldsymbol{V}}^{*}$. Without loss of generality, we assume that eigenvectors in $\bar{\boldsymbol{V}}^{*}$ are sorted such that the first entry of $\boldsymbol{e}^{\dagger}$ is non-zero, i.e., the first column of $\bar{\boldsymbol{V}}^{*}$ is proportional to the initial perturbation $\Delta\bar{\boldsymbol{w}}(t_0)$ (cf. Eq. 167). Next, we will derive the transformed Jacobian.

5.2.1 The transformed Jacobian

First, we consider the regular Jacobian $\bar{\boldsymbol{J}}^{*}$. We rewrite the dynamics in Eq. 159 as[1]

$$\dot{\bar{\boldsymbol{w}}} = \left[\mathbb{1} - \frac{(\bar{\boldsymbol{w}}_{EF} + \bar{\boldsymbol{w}}_{EE})\bar{\boldsymbol{c}}_{EE}{}^{\mathsf{T}}}{\bar{\boldsymbol{c}}_{EE}{}^{\mathsf{T}}(\bar{\boldsymbol{w}}_{EF} + \bar{\boldsymbol{w}}_{EE})} - \ldots\right]\bar{\boldsymbol{C}}\bar{\boldsymbol{w}}, \qquad \bar{\boldsymbol{w}}_{EF} = \begin{pmatrix}\boldsymbol{w}_{EF}\\ 0\\ 0\\ \boldsymbol{0}\\ 0\\ 0\end{pmatrix}, \quad \bar{\boldsymbol{w}}_{EE} = \begin{pmatrix}\boldsymbol{0}\\ w_{EE}\\ 0\\ \boldsymbol{0}\\ 0\\ 0\end{pmatrix}, \quad \bar{\boldsymbol{c}}_{EE} = \begin{pmatrix}\boldsymbol{c}\\ 1\\ 0\\ \boldsymbol{0}\\ 0\\ 0\end{pmatrix}, \tag{172}$$

where the second term in the bracket corresponds to the normalization of all excitatory synapses onto the excitatory neuron, additional normalization terms are indicated by ellipsis[2] (cf. Eq. 45), and $\boldsymbol{c}$ is a vector of ones. Then the Jacobian has the following shape (cf. Eq. 29)

$$\bar{\boldsymbol{J}}^{*} = \left.\frac{\mathrm{d}\dot{\bar{\boldsymbol{w}}}}{\mathrm{d}\bar{\boldsymbol{w}}}\right|_{*} = \left[\mathbb{1} - \frac{(\bar{\boldsymbol{v}}^{*}_{EF} + \bar{\boldsymbol{v}}^{*}_{EE})\bar{\boldsymbol{c}}_{EE}{}^{\mathsf{T}}}{\bar{\boldsymbol{c}}_{EE}{}^{\mathsf{T}}(\bar{\boldsymbol{v}}^{*}_{EF} + \bar{\boldsymbol{v}}^{*}_{EE})} - \ldots\right]\left(\bar{\boldsymbol{C}}^{*} - \bar{\lambda}^{*}\mathbb{1} + \left.\frac{\mathrm{d}\bar{\boldsymbol{C}}}{\mathrm{d}\bar{\boldsymbol{w}}}\right|_{*}\bar{\boldsymbol{w}}^{*}\right), \tag{173}$$

where $\bar{\boldsymbol{v}}^{*}_{EF}$, $\bar{\boldsymbol{v}}^{*}_{EE}$ have the same shape as $\bar{\boldsymbol{w}}_{EF}$, $\bar{\boldsymbol{w}}_{EE}$ in Eq. 172 with entries corresponding to the respective entries of the regular eigenvector $\bar{\boldsymbol{v}}^{*}$ (cf. Eq. 166). Note that we accounted for the weight dependence of the modified covariance matrix $\bar{\boldsymbol{C}}$ which results in the tensor $\mathrm{d}\bar{\boldsymbol{C}}/\mathrm{d}\bar{\boldsymbol{w}}$. To find the transformed Jacobian $\bar{\boldsymbol{V}}^{*-1}\bar{\boldsymbol{J}}^{*}\bar{\boldsymbol{V}}^{*}$, we consider the first bracket:

$$\bar{\boldsymbol{V}}^{*-1}\left[\mathbb{1} - \frac{(\bar{\boldsymbol{v}}^{*}_{EF} + \bar{\boldsymbol{v}}^{*}_{EE})\bar{\boldsymbol{c}}_{EE}{}^{\mathsf{T}}}{\bar{\boldsymbol{c}}_{EE}{}^{\mathsf{T}}(\bar{\boldsymbol{v}}^{*}_{EF} + \bar{\boldsymbol{v}}^{*}_{EE})} - \ldots\right]\bar{\boldsymbol{V}}^{*} \tag{174}$$

The first entry remains equal to the identity matrix, as the eigenvector matrix and its inverse cancel. We consider the columns $\bar{\boldsymbol{v}}^{*}_b$ of $\bar{\boldsymbol{V}}^{*}$ separately. Then, we can write

$$\left[-\frac{(\bar{\boldsymbol{v}}^{*}_{EF} + \bar{\boldsymbol{v}}^{*}_{EE})\bar{\boldsymbol{c}}_{EE}{}^{\mathsf{T}}}{\bar{\boldsymbol{c}}_{EE}{}^{\mathsf{T}}(\bar{\boldsymbol{v}}^{*}_{EF} + \bar{\boldsymbol{v}}^{*}_{EE})} - \ldots\right]\bar{\boldsymbol{v}}^{*}_b = -(\bar{\boldsymbol{v}}^{*}_{EF} + \bar{\boldsymbol{v}}^{*}_{EE})h^b_{EE} - \bar{\boldsymbol{v}}^{*}_{EI}h^b_{EI} - (\bar{\boldsymbol{v}}^{*}_{IF} + \bar{\boldsymbol{v}}^{*}_{IE})h^b_{IE} - \bar{\boldsymbol{v}}^{*}_{II}h^b_{II} = -\boldsymbol{H}_b\bar{\boldsymbol{v}}^{*}, \tag{175}$$

$$\boldsymbol{H}_b \equiv \begin{pmatrix}\mathbb{1}h^b_{EE} &&&&& \\ & h^b_{EE} &&&& \\ && h^b_{EI} &&& \\ &&& \mathbb{1}h^b_{IE} && \\ &&&& h^b_{IE} & \\ &&&&& h^b_{II}\end{pmatrix}, \quad h^b_{EE} \equiv \frac{\bar{\boldsymbol{c}}_{EE}{}^{\mathsf{T}}\bar{\boldsymbol{v}}^{*}_b}{\bar{\boldsymbol{c}}_{EE}{}^{\mathsf{T}}(\bar{\boldsymbol{v}}^{*}_{EF} + \bar{\boldsymbol{v}}^{*}_{EE})}, \quad h^b_{EI} \equiv \frac{\bar{\boldsymbol{c}}_{EI}{}^{\mathsf{T}}\bar{\boldsymbol{v}}^{*}_b}{\bar{\boldsymbol{c}}_{EI}{}^{\mathsf{T}}\bar{\boldsymbol{v}}^{*}_{EI}}, \tag{176}$$

[1] Remember that we set $\bar{\boldsymbol{\tau}} = \mathbb{1}$.

[2] In general, there are $2 \times (n_E + n_I)$ normalization terms.

where $\boldsymbol{H}_b$ is a diagonal matrix with entries corresponding to the respective normalization constraint, of which we give h^b_{EE} and h^b_{EI} as examples. Then each column $\bar{\boldsymbol{v}}^*_b$ of $\bar{\boldsymbol{V}}^*$ is transformed into a multiple of the separately normalized parts of the fixed point eigenvector $\bar{\boldsymbol{v}}^*$ (Eq. 166). After transformation, the bth column becomes

$$\bar{\boldsymbol{V}}^{*-1}\boldsymbol{H}_b\bar{\boldsymbol{v}}^* = \mathcal{N}^{-1}\begin{pmatrix} \mathcal{N}\,{\boldsymbol{V}_{\backslash *}}^{\mathsf{T}} & \mathbf{0} & \mathbf{0} & \\ {\boldsymbol{v}^*}^{\mathsf{T}} & a^*_E & -a^*_I & \\ a_E{\boldsymbol{v}^*}^{\mathsf{T}} & -(1-a^{*2}_I) & -a^*_E a^*_I & \mathbf{0} \\ -a_I{\boldsymbol{v}^*}^{\mathsf{T}} & -a^*_I a^*_E & 1+a^{*2}_E & \\ & \mathbf{0} & & \ddots \end{pmatrix}\begin{pmatrix} h^b_{EE}\boldsymbol{v}^* \\ h^b_{EE}a^*_E \\ h^b_{EI}a^*_I \\ \vdots \end{pmatrix} = \begin{pmatrix} \mathbf{0} \\ \vdots \end{pmatrix}, \quad [177]$$

where, as before, ellipsis indicate potentially non-zero entries. Importantly, after the transformation, the first $N_F - 1$ entries are zero, independent of the column index, b, because $\boldsymbol{v}^*$ is orthogonal to the columns of $\boldsymbol{V}_{\backslash *}$. Overall, we can write

$$\Rightarrow \quad \bar{\boldsymbol{V}}^{*-1}\left[\mathbb{1} - \frac{(\bar{\boldsymbol{v}}^*_{EF}+\bar{\boldsymbol{v}}^*_{EE}){\bar{\boldsymbol{c}}_{EE}}^{\mathsf{T}}}{{\bar{\boldsymbol{c}}_{EE}}^{\mathsf{T}}(\bar{\boldsymbol{v}}^*_{EF}+\bar{\boldsymbol{v}}^*_{EE})} - \ldots\right]\bar{\boldsymbol{V}}^* = \begin{pmatrix} \mathbb{1} & \mathbf{0} & \mathbf{0} & \mathbf{0} & \\ \vdots & \vdots & \vdots & \vdots & \mathbf{0} \\ & & \mathbf{0} & & \ddots \end{pmatrix}, \quad [178]$$

where the block structure arises from the block structure of $\bar{\boldsymbol{V}}^{*-1}$ (cf. Eq. 177).

After transformation, the second bracket of Eq. 173 becomes

$$\bar{\boldsymbol{V}}^{*-1}\left(\bar{\boldsymbol{C}}^* - \bar{\lambda}^*\mathbb{1} + \left.\frac{\mathrm{d}\bar{\boldsymbol{C}}}{\mathrm{d}\bar{\boldsymbol{w}}}\right|_* \bar{\boldsymbol{w}}^*\right)\bar{\boldsymbol{V}}^* = \left(\bar{\Lambda}^* - \bar{\lambda}^*\mathbb{1} + \bar{\boldsymbol{V}}^{*-1}\left.\frac{\mathrm{d}\bar{\boldsymbol{C}}}{\mathrm{d}\bar{\boldsymbol{w}}}\right|_* \bar{\boldsymbol{w}}^*\bar{\boldsymbol{V}}^*\right). \quad [179]$$

We next consider the first columns of $\left.\frac{\mathrm{d}\bar{\boldsymbol{C}}}{\mathrm{d}\bar{\boldsymbol{w}}}\right|_*\bar{\boldsymbol{w}}^*$, for which we compute the matrix $\left.\frac{\mathrm{d}\bar{\boldsymbol{C}}}{\mathrm{d}w^b_{EF}}\right|_*$, where w^b_{EF} is the bth feed-forward weight onto the excitatory neuron.

$$\left.\frac{\mathrm{d}\bar{\boldsymbol{C}}}{\mathrm{d}w^b_{EF}}\right|_* = \frac{\mathrm{d}}{\mathrm{d}w^b_{EF}}\left.\begin{pmatrix} \boldsymbol{C} & \boldsymbol{C}\boldsymbol{a}_E & -\boldsymbol{C}\boldsymbol{a}_I & \\ {\boldsymbol{a}_E}^{\mathsf{T}}\boldsymbol{C} & {\boldsymbol{a}_E}^{\mathsf{T}}\boldsymbol{C}\boldsymbol{a}_E & -{\boldsymbol{a}_E}^{\mathsf{T}}\boldsymbol{C}\boldsymbol{a}_I & \mathbf{0} \\ {\boldsymbol{a}_I}^{\mathsf{T}}\boldsymbol{C} & {\boldsymbol{a}_I}^{\mathsf{T}}\boldsymbol{C}\boldsymbol{a}_E & -{\boldsymbol{a}_I}^{\mathsf{T}}\boldsymbol{C}\boldsymbol{a}_I & \\ & \mathbf{0} & & \ddots \end{pmatrix}\right|_* \quad [180]$$

$$= \begin{pmatrix} \mathbf{0} & \boldsymbol{C}\left.\frac{\mathrm{d}\boldsymbol{a}_E}{\mathrm{d}w^b_{EF}}\right|_* & -\boldsymbol{C}\left.\frac{\mathrm{d}\boldsymbol{a}_I}{\mathrm{d}w^b_{EF}}\right|_* & \\ \left.\frac{\mathrm{d}{\boldsymbol{a}_E}^{\mathsf{T}}}{\mathrm{d}w^b_{EF}}\right|_*\boldsymbol{C} & \left(\left.\frac{\mathrm{d}{\boldsymbol{a}_E}^{\mathsf{T}}}{\mathrm{d}w^b_{EF}}\right|_*\boldsymbol{C}\boldsymbol{a}^*_E + {\boldsymbol{a}^*_E}^{\mathsf{T}}\boldsymbol{C}\left.\frac{\mathrm{d}\boldsymbol{a}_E}{\mathrm{d}w^b_{EF}}\right|_*\right) & -\left(\left.\frac{\mathrm{d}{\boldsymbol{a}_E}^{\mathsf{T}}}{\mathrm{d}w^b_{EF}}\right|_*\boldsymbol{C}\boldsymbol{a}^*_I + {\boldsymbol{a}^*_E}^{\mathsf{T}}\boldsymbol{C}\left.\frac{\mathrm{d}\boldsymbol{a}_I}{\mathrm{d}w^b_{EF}}\right|_*\right) & \mathbf{0} \\ \left.\frac{\mathrm{d}{\boldsymbol{a}_I}^{\mathsf{T}}}{\mathrm{d}w^b_{EF}}\right|_*\boldsymbol{C} & \left(\left.\frac{\mathrm{d}{\boldsymbol{a}_I}^{\mathsf{T}}}{\mathrm{d}w^b_{EF}}\right|_*\boldsymbol{C}\boldsymbol{a}^*_E + {\boldsymbol{a}^*_I}^{\mathsf{T}}\boldsymbol{C}\left.\frac{\mathrm{d}\boldsymbol{a}_E}{\mathrm{d}w^b_{EF}}\right|_*\right) & -\left(\left.\frac{\mathrm{d}{\boldsymbol{a}_I}^{\mathsf{T}}}{\mathrm{d}w^b_{EF}}\right|_*\boldsymbol{C}\boldsymbol{a}^*_I + {\boldsymbol{a}^*_I}^{\mathsf{T}}\boldsymbol{C}\left.\frac{\mathrm{d}\boldsymbol{a}_I}{\mathrm{d}w^b_{EF}}\right|_*\right) & \\ & \mathbf{0} & & \ddots \end{pmatrix}, \quad [181]$$

where we used the definition of $\bar{\boldsymbol{C}}$ from Eq. 158. The vectors $\boldsymbol{a}_E$ and $\boldsymbol{a}_I$ are defined in Eq. 155 & 156. It follows:

$$\left.\frac{\mathrm{d}\boldsymbol{a}_E}{\mathrm{d}w^b_{EF}}\right|_* = \frac{1}{1 - w^*_{EE} + \dfrac{w^*_{EI}w^*_{IE}}{1+w^*_{II}}}\boldsymbol{e}_b \equiv \mu_E\boldsymbol{e}_b, \quad [182]$$

$$\left.\frac{\mathrm{d}\boldsymbol{a}_I}{\mathrm{d}w^b_{EF}}\right|_* = \frac{1}{1 + w^*_{II} + \dfrac{w^*_{IE}w^*_{EI}}{1-w^*_{EE}}}\frac{w^*_{IE}}{1-w^*_{EE}}\boldsymbol{e}_b \equiv \mu_I\boldsymbol{e}_b, \quad [183]$$

where $\boldsymbol{e}_b$ is a vector of dimension N_F with entries equal to zero, except for the bth entry equal to one. Additionally, we have (cf. Eqs.161 & 162)

$$\boldsymbol{C}\boldsymbol{a}^*_E = \lambda^* a^*_E\boldsymbol{v}^*, \quad \boldsymbol{C}\boldsymbol{a}^*_I = \lambda^* a^*_I\boldsymbol{v}^*, \quad [184]$$

which results in

$$\Rightarrow \quad \frac{\mathrm{d}\bar{\boldsymbol{C}}}{\mathrm{d}w_{EF}^{b}}\bigg|_{*} = \begin{pmatrix} \boldsymbol{0} & \mu_E \boldsymbol{C}\boldsymbol{e}_b & -\mu_I \boldsymbol{C}\boldsymbol{e}_b & \\ \mu_E \boldsymbol{e}_b{}^{\mathsf{T}}\boldsymbol{C} & 2\lambda^* a_E^* \mu_E \boldsymbol{v}^{*\mathsf{T}}\boldsymbol{e}_b & -\lambda^*(\mu_E a_E^* + \mu_I a_E^*)\boldsymbol{v}^{*\mathsf{T}}\boldsymbol{e}_b & \boldsymbol{0} \\ \mu_I \boldsymbol{e}_b{}^{\mathsf{T}}\boldsymbol{C} & \lambda^*(\mu_I a_E^* + \mu_E a_I^*)\boldsymbol{v}^{*\mathsf{T}}\boldsymbol{e}_b & 2\lambda^* a_I^* \mu_I \boldsymbol{v}^{*\mathsf{T}}\boldsymbol{e}_b & \\ & \boldsymbol{0} & & \ddots \end{pmatrix}, \tag{185}$$

$$\Rightarrow \quad \frac{\mathrm{d}\bar{\boldsymbol{C}}}{\mathrm{d}w_{EF}^{b}}\bigg|_{*} \bar{\boldsymbol{w}}^* = \begin{pmatrix} \beta_E \boldsymbol{C}\boldsymbol{e}_b \\ g_1 \boldsymbol{v}^{*\mathsf{T}}\boldsymbol{e}_b \\ g_2 \boldsymbol{v}^{*\mathsf{T}}\boldsymbol{e}_b \\ \boldsymbol{0} \end{pmatrix}, \quad \bar{\boldsymbol{w}}^* = \bar{\boldsymbol{v}}^* = \begin{pmatrix} \boldsymbol{v}^* \\ w_{EE}^* \\ w_{EI}^* \\ \vdots \end{pmatrix}, \quad \beta_E = \mu_E w_{EE}^* - \mu_I w_{EI}^*, \tag{186}$$

where $g_{(\cdot)}$ are scalars.

$$\Rightarrow \quad \frac{\mathrm{d}\bar{\boldsymbol{C}}}{\mathrm{d}\boldsymbol{w}_{EF}}\bigg|_{*} \boldsymbol{w}^* = \begin{pmatrix} \beta_E \boldsymbol{C} \\ g_1 \boldsymbol{v}^{*\mathsf{T}} \\ g_2 \boldsymbol{v}^{*\mathsf{T}} \\ \boldsymbol{0} \end{pmatrix}. \tag{187}$$

We find other columns in a similar fashion and write

$$\Rightarrow \quad \frac{\mathrm{d}\bar{\boldsymbol{C}}}{\mathrm{d}\bar{\boldsymbol{w}}}\bigg|_{*} \boldsymbol{w}^* = \begin{pmatrix} \beta_E \boldsymbol{C} & g_3 \boldsymbol{v}^* & g_6 \boldsymbol{v}^* & \\ g_1 \boldsymbol{v}^{*\mathsf{T}} & g_4 & g_7 & \boldsymbol{0} \\ g_2 \boldsymbol{v}^{*\mathsf{T}} & g_5 & g_8 & \\ & \boldsymbol{0} & & \ddots \end{pmatrix}, \tag{188}$$

where, again, $g_{(\cdot)}$ are scalars. After applying the transformation, we get

$$\bar{\boldsymbol{V}}^{*-1} \frac{\mathrm{d}\bar{\boldsymbol{C}}}{\mathrm{d}\bar{\boldsymbol{w}}}\bigg|_{*} \bar{\boldsymbol{w}}^* \bar{\boldsymbol{V}}^* = \bar{\boldsymbol{V}}^{*-1} \begin{pmatrix} \beta_E \boldsymbol{C} & g_3 \boldsymbol{v}^* & g_6 \boldsymbol{v}^* & \\ g_1 \boldsymbol{v}^{*\mathsf{T}} & g_4 & g_7 & \boldsymbol{0} \\ g_2 \boldsymbol{v}^{*\mathsf{T}} & g_5 & g_8 & \\ & \boldsymbol{0} & & \ddots \end{pmatrix} \begin{pmatrix} \boldsymbol{V}_{\setminus *} & \boldsymbol{v}^* & \boldsymbol{v}^* a_E^* & \boldsymbol{v}^* a_I^* & \\ \boldsymbol{0} & a_E^* & -1 & 0 & \boldsymbol{0} \\ \boldsymbol{0} & a_I^* & 0 & 1 & \\ & & \boldsymbol{0} & & \ddots \end{pmatrix} \tag{189}$$

$$= \bar{\boldsymbol{V}}^{*-1} \begin{pmatrix} \beta_E \boldsymbol{C}\boldsymbol{V}_{\setminus *} & g_9 \boldsymbol{v}^* & g_{12} \boldsymbol{v}^* & g_{15} \boldsymbol{v}^* & \\ \boldsymbol{0} & g_{10} & g_{13} & g_{16} & \boldsymbol{0} \\ \boldsymbol{0} & g_{11} & g_{14} & g_{17} & \\ & \boldsymbol{0} & & & \ddots \end{pmatrix} \tag{190}$$

$$= \mathcal{N}^{-1} \begin{pmatrix} \mathcal{N}\boldsymbol{V}_{\setminus *}{}^{\mathsf{T}} & \boldsymbol{0} & \boldsymbol{0} & \\ \boldsymbol{v}^{*\mathsf{T}} & a_E^* & -a_I^* & \\ a_E \boldsymbol{v}^{*\mathsf{T}} & -(1 - a_I^{*2}) & -a_E^* a_I^* & \boldsymbol{0} \\ -a_I \boldsymbol{v}^{*\mathsf{T}} & -a_I^* a_E^* & 1 + a_E^{*2} & \\ & \boldsymbol{0} & & \ddots \end{pmatrix} \begin{pmatrix} \beta_E \boldsymbol{V}_{\setminus *}\Lambda_{\setminus *} & g_9 \boldsymbol{v}^* & g_{12} \boldsymbol{v}^* & g_{15} \boldsymbol{v}^* & \\ \boldsymbol{0} & g_{10} & g_{13} & g_{16} & \boldsymbol{0} \\ \boldsymbol{0} & g_{11} & g_{14} & g_{17} & \\ & \boldsymbol{0} & & & \ddots \end{pmatrix} \tag{191}$$

$$= \begin{pmatrix} \beta_E \Lambda_{\setminus *} & \boldsymbol{0} & \boldsymbol{0} & \boldsymbol{0} & \\ \boldsymbol{0} & g_{18} & g_{21} & g_{24} & \\ \boldsymbol{0} & g_{19} & g_{22} & g_{25} & \boldsymbol{0} \\ \boldsymbol{0} & g_{20} & g_{23} & g_{26} & \\ & \boldsymbol{0} & & & \ddots \end{pmatrix}. \tag{192}$$

The fully transformed Jacobian is (cf. Eq. 179)

$$\bar{\boldsymbol{J}}_v^* = \bar{\boldsymbol{V}}^{*-1}\bar{\boldsymbol{J}}^*\bar{\boldsymbol{V}}^* = \bar{\boldsymbol{V}}^{*-1}\left[\mathbb{1} - \frac{(\bar{\boldsymbol{v}}_{EF}^* + \bar{\boldsymbol{v}}_{EE}^*)\bar{\boldsymbol{c}}_{EE}{}^{\top}}{\bar{\boldsymbol{c}}_{EE}{}^{\top}(\bar{\boldsymbol{v}}_{EF}^* + \bar{\boldsymbol{v}}_{EE}^*)} - \ldots\right]\bar{\boldsymbol{V}}^*\left(\bar{\Lambda}^* - \bar{\lambda}^*\mathbb{1} + \bar{\boldsymbol{V}}^{*-1}\left.\frac{\mathrm{d}\bar{\boldsymbol{C}}}{\mathrm{d}\bar{\boldsymbol{w}}}\right|_*\bar{\boldsymbol{w}}^*\bar{\boldsymbol{V}}^*\right) \qquad [193]$$

Finally, by inserting Eq. 178 & 192 we find

$$\bar{\boldsymbol{V}}^{*-1}\bar{\boldsymbol{J}}^*\bar{\boldsymbol{V}}^* = \begin{pmatrix} \mathbb{1} & \mathbf{0} & \mathbf{0} & \mathbf{0} & \\ \vdots & \vdots & \vdots & \vdots & \mathbf{0} \\ & \mathbf{0} & & & \ddots \end{pmatrix}\left(\bar{\Lambda}^* - \bar{\lambda}^*\mathbb{1} + \begin{pmatrix} \beta_E\boldsymbol{\Lambda}_{\backslash *} & \mathbf{0} & \mathbf{0} & \mathbf{0} & \\ \mathbf{0} & g_{18} & g_{21} & g_{24} & \\ \mathbf{0} & g_{19} & g_{22} & g_{25} & \mathbf{0} \\ \mathbf{0} & g_{20} & g_{23} & g_{26} & \\ & \mathbf{0} & & & \ddots \end{pmatrix}\right). \qquad [194]$$

$$\Rightarrow \quad \bar{\boldsymbol{J}}_v^* = \begin{pmatrix} \left(\bar{\Lambda}_{\backslash *} - \bar{\lambda}^*\mathbb{1} + \beta_E\boldsymbol{\Lambda}_{\backslash *}\right) & \mathbf{0} & \mathbf{0} & \mathbf{0} & \\ \vdots & \vdots & \vdots & \vdots & \mathbf{0} \\ \mathbf{0} & & & & \ddots \end{pmatrix}, \qquad [195]$$

where $\bar{\Lambda}_{\backslash *}$ contains eigenvalues of $\bar{\boldsymbol{C}}^*$ that correspond to regular, non-fixed point eigenvectors.[1]

5.2.2 Stability conditions

The dynamics of a general fixed point perturbation $\Delta\boldsymbol{w}_v$ in the eigenbasis of $\bar{\boldsymbol{C}}^*$ is (cf. Eq.171)

$$\frac{\mathrm{d}}{\mathrm{d}t}\Delta\bar{\boldsymbol{w}}_v = \bar{\boldsymbol{J}}_v^*\,\Delta\bar{\boldsymbol{w}}_v = \begin{pmatrix} \left(\bar{\Lambda}_{\backslash *} - \bar{\lambda}^*\mathbb{1} + \beta_E\boldsymbol{\Lambda}_{\backslash *}\right) & \mathbf{0} & \mathbf{0} & \\ \vdots & \vdots & \vdots & \mathbf{0} \\ \mathbf{0} & & & \ddots \end{pmatrix}\Delta\bar{\boldsymbol{w}}_v. \qquad [196]$$

Note that the transformed Jacobian (Eq. 195) has a triangular block structure, and each row of $\bar{\boldsymbol{J}}_v^*$ corresponds to the growth of a perturbation in the direction of a different eigenvector of $\bar{\boldsymbol{C}}^*$. We are only interested in perturbations that grow in the direction of a non-fixed point feedforward eigenvector, $\boldsymbol{V}_{\backslash *}$. Therefore, we focus on the first rows of $\bar{\boldsymbol{J}}_v^*$, which correspond to growth in these directions. Except for the first diagonal block, these rows are zero. It follows that perturbations $\Delta\bar{\boldsymbol{w}}_v(t_0)$ that do not already contain components in the direction of non-fixed point eigenvectors, also do not develop such components in their later dynamics. In contrast, perturbations in the direction of a non-fixed point feedforward input mode, e.g., $\Delta\bar{\boldsymbol{w}}_v \propto \boldsymbol{e}^\dagger$, can induce perturbations within the original eigencircuit that corresponds to the feedforward eigenvector $\boldsymbol{v}^*$ [2]. For example, a decrease in feedforward and recurrent excitatory synaptic weights within the eigencircuit balances the increase of feedforward excitatory synaptic weights due to the perturbation towards a different eigencircuit, to maintain the weight norm. However, as explained above, these 'second-order' perturbations, without components in the direction of non-fixed point feedforward eigenvectors, $\boldsymbol{V}_{\backslash *}$, are contained within the eigencircuit, i.e., they can not induce subsequent perturbations in the direction of non-fixed point feedforward input modes, $\boldsymbol{V}_{\backslash *}$. Therefore, to answer the question of when an eigencircuit is stable, we only consider the dynamics along the direction of the original perturbation (cf. Eq.167) by projecting the dynamics onto the perturbation vector at time zero, $\Delta\bar{\boldsymbol{w}}_v(t_0) \propto \boldsymbol{e}^\dagger$ (cf. Eq.170):

$$\boldsymbol{e}^{\dagger\top}\frac{\mathrm{d}}{\mathrm{d}t}\Delta\bar{\boldsymbol{w}}_v = \left(\bar{\lambda}^\dagger - \bar{\lambda}^* + \beta_E\lambda^\dagger\right)\boldsymbol{e}^{\dagger\top}\Delta\bar{\boldsymbol{w}}_v, \qquad [197]$$

which provides the eigencircuit stability condition for the excitatory neuron

$$\boxed{\bar{\lambda}^\dagger - \bar{\lambda}^* + \beta_E\lambda^\dagger < 0}\,. \qquad [198]$$

[1] Note that in our specific network the top left block of $\bar{\Lambda}^*$ is equal to $\boldsymbol{\Lambda}_{\backslash *}$, i.e., $\bar{\Lambda}_{\backslash *} = \boldsymbol{\Lambda}_{\backslash *}$, because there are no neurons tuned to the respective feedforward eigenvectors. In particular, $\bar{\lambda}^\dagger = \lambda^\dagger$ (cf. Eq. 152)

[2] This is due to the potentially non-zero elements in the block below the top left diagonal block of $\bar{\boldsymbol{J}}_v^*$ in Equation 196.

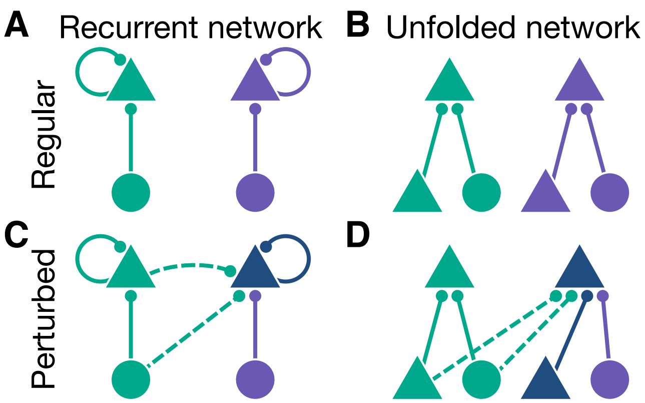

Figure S4: (*A*) Two excitatory neurons (triangles) are tuned to two different, but equally attractive input modes (circles, green and purple). (*B*) The same circuit as in *A*, unfolded to highlight pre-synaptic partners. Both input modes are balanced in their attraction. (*C*) Perturbing the purple excitatory neuron towards the green input mode (dashed lines) shifts its tuning (dark blue) such that it now responds to both the green and the purple input modes. (*D*) The unfolded circuit from *C*. Due to the perturbation, the green input mode is now more attractive, and the previously purple excitatory neuron shifts its tuning. See text for details.

If Eq. 198 holds, perturbations in the direction of non-fixed point eigenvectors decay to zero, and the eigencircuit is stable. For β_E we have (cf. Eqs. 186 & 182 f.)

$$\beta_E = \frac{1}{1 - w^*_{EE} + \frac{w^*_{EI} w^*_{IE}}{1 + w^*_{II}}} w^*_{EE} - \frac{1}{1 + w^*_{II} + \frac{w^*_{IE} w^*_{EI}}{1 - w^*_{EE}}} \left(\frac{w^*_{EI} w^*_{IE}}{1 - w^*_{EE}} \right). \quad [199]$$

From Eq. 155 & 156 we find

$$\left. \frac{\mathrm{d}y_E}{\mathrm{d}(\boldsymbol{w}_{EF}{}^{\mathsf{T}} \boldsymbol{y})} \right|_* = \frac{1}{1 - w^*_{EE} + \frac{w^*_{EI} w^*_{IE}}{1 + w^*_{II}}}, \quad [200]$$

$$\left. \frac{\mathrm{d}y_I}{\mathrm{d}(\boldsymbol{w}_{EF}{}^{\mathsf{T}} \boldsymbol{y})} \right|_* = \frac{1}{1 + w^*_{II} + \frac{w^*_{IE} w^*_{EI}}{1 - w^*_{EE}}} \frac{w^*_{IE}}{1 - w^*_{EE}}, \quad [201]$$

and we get

$$\Rightarrow \boxed{\beta_E = \left. \frac{\mathrm{d}y_E}{\mathrm{d}(\boldsymbol{w}_{EF}{}^{\mathsf{T}} \boldsymbol{y})} \right|_* w^*_{EE} - \left. \frac{\mathrm{d}y_I}{\mathrm{d}(\boldsymbol{w}_{EF}{}^{\mathsf{T}} \boldsymbol{y})} \right|_* w^*_{EI}}. \quad [202]$$

Following the same framework, we find the stability condition when perturbing the inhibitory neuron:

$$\boxed{\bar{\lambda}^\dagger - \bar{\lambda}^* + \beta_I \lambda^\dagger < 0}, \quad [203]$$

$$\Rightarrow \boxed{\beta_I = \left. \frac{\mathrm{d}y_E}{\mathrm{d}(\boldsymbol{w}_{IF}{}^{\mathsf{T}} \boldsymbol{y})} \right|_* w^*_{IE} - \left. \frac{\mathrm{d}y_I}{\mathrm{d}(\boldsymbol{w}_{IF}{}^{\mathsf{T}} \boldsymbol{y})} \right|_* w^*_{II}}. \quad [204]$$

We will now interpret these results.

5.2.3 Eigencircuit stability depends on recurrent connectivity

We first consider the case when the effective attraction of all eigencircuits is the same, i.e., $\bar{\lambda}^* = \bar{\lambda}^\dagger$ (cf. Eqs. 198 & 203). Then the stability is fully determined by β_E, and β_I. In feedforward circuits we have not found any β-terms, because in that case, the modified covariance matrix does not depend on any plastic synaptic weights (cf. Eq. 51 & Sec. 2.2). This is not the case in recurrent circuits where the perturbation induces a change in the tuning of laterally projecting neurons.

To build some intuition, we consider a simple example: Think of a recurrent network of two excitatory neurons with identical weight norms , and an external population of excitatory neurons projecting feedforward input to both. In the fixed point, the neurons are tuned to two different feedforward input eigenvectors of equal attraction and are recurrently connected to themselves but not each other (Fig. S4*A*). Then the effective attraction of the two eigencircuits is the same. In general, neurons receive synaptic inputs, but have no information about the overall network structure, e.g., which synaptic inputs are feedforward or recurrent. Taking this perspective, we unfold the recurrent network and observe that the effective mode attraction is a combination of the feedforward input and the recurrent self-excitation (Fig. S4*B*). When we perturb one neuron towards the opposing input mode (Fig. S4*C*, dashed lines), the tuning of the perturbed neuron changes slightly in the direction of that mode (Fig. S4*C*, dark blue). From the perspective of the perturbed neuron, this tuning change leads to an attraction increase of the opposing eigencircuit, which is now more attractive than the original eigencircuit of the perturbed neuron (Fig. S4*D*), and the perturbation grows in the direction of the more attractive mode – the fixed point is unstable. Similarly, if the neurons were inhibitory instead, the perturbation would decrease the attraction towards the opposite input mode which would stabilize the network.

In our mathematical analysis of the circuit shown in Figure S3*D*, the attraction increase or decrease due to the tuning change of recurrently projecting neurons is reflected in the β-terms in Equations 198 & 203, which emerge from the weight dependence of the modified covariance matrix $\bar{\boldsymbol{C}}$ (cf. Eq. 173). For example, when perturbing the excitatory neuron, the increase in attraction from the perspective of the perturbed neuron is (cf. Eq. 198)

$$\beta_E \lambda^{\dagger} = \left(\left.\frac{\mathrm{d}y_E}{\mathrm{d}(\boldsymbol{w}_{EF}{}^{\top}\boldsymbol{y})}\right|_{*} \lambda^{\dagger} \right) w^{*}_{EE} - \left(\left.\frac{\mathrm{d}y_I}{\mathrm{d}(\boldsymbol{w}_{EF}{}^{\top}\boldsymbol{y})}\right|_{*} \lambda^{\dagger} \right) w^{*}_{EI}, \qquad [205]$$

where the brackets reflect the tuning shifts of the excitatory and the inhibitory neuron[1] in response to the perturbation of $\boldsymbol{w}_{EF}$ in the direction of $\boldsymbol{v}^{\dagger}$, which is then weighted by the respective synaptic connection onto the excitatory neuron, w^{*}_{EE}, w^{*}_{EI}. When the inhibitory neuron is perturbed instead, the terms for β_I follow the same logic (cf. Eq. 204).

Without going through the lengthy mathematical derivation, we now give some intuition about β-terms of the network perturbation in Figure S3*C*. In the fixed point, the perturbed excitatory neuron receives recurrent input from all neurons in its eigencircuit, including itself. In the following, superscripts indicate the corresponding eigencircuit, *A* or *B*. Then, as for the equivalent circuit (cf. Fig. S3*D*), β^A_E comprises two terms, one due to the tuning shift of y^A_E, and a second due to the tuning shift of y^A_I. Assuming the same weight norms, this is exactly equal to the β_E for the equivalent circuit (Eq. 198). Different from β_E, β^A_E is weighted with the effective attraction $\bar{\lambda}^B = \lambda^B + \lambda^B_{\text{eig}}$, instead of only the feedforward attraction (cf. $\lambda^{\dagger}$ in Eq. 205), because the perturbation comprises not only the feedforward eigenvector component but the whole eigencircuit (cf. dashed lines in Figs. S3*C* & *D*). This is why, for the equivalent circuit, we chose the feedforward attraction $\lambda^{\dagger} = \bar{\lambda}^B = \lambda^B + \lambda^B_{\text{eig}}$ (Eq. 152). Then, the diagonal entries corresponding to the respective perturbations in the upper left blocks of the transformed Jacobians are the same[2] (cf. Eq. 195), i.e.,

$$\bar{\lambda}^{\dagger} - \bar{\lambda}^{*} + \beta_E \lambda^{\dagger} = \bar{\lambda}^B - \bar{\lambda}^A + \beta^B_E \bar{\lambda}^B. \qquad [206]$$

We find that perturbations in both circuits initially follow the same dynamics, while the later dynamics diverges: At time t_0, there are no lateral projections from eigencircuit *B* towards eigencircuit *A* (cf. Fig. S3*C*), since in the fixed point there are no recurrent connections between eigencircuits (cf. Sec. 4), and the perturbation at time t_0 only introduces connections from eigencircuit *B* onto eigencircuit *A*. However, as we just discussed, the perturbation introduces a tuning shift in neurons of eigencircuit *A* in the direction of eigencircuit *B*. This shift leads to non-zero correlations between neurons of both eigencircuits, and synaptic weights from eigencircuit *A* onto eigencircuit *B* start to grow. These growing synapses shift the attraction of neurons in eigencircuit *B* and thus impact the dynamics of perturbation components in the direction of eigencircuit *B*. Therefore, the transformed Jacobian of the original circuit (Fig. S3*C*) has a more complex structure than the Jacobian for the equivalent circuit[3]. However, since we consider an initial perturbation that is aligned with a regular eigenvector, i.e., $\Delta\bar{\boldsymbol{w}}_v(t_0) \propto \boldsymbol{e}^B$ is one-hot (cf. Eq. 170), the top left diagonal block of the Jacobian still determines the initial dynamics[4].

[1] Note that also the tuning of the inhibitory neuron changes, although it is not directly perturbed.

[2] Recall that $\lambda^{\dagger}_{\text{eig}} = 0$ and, therefore, $\bar{\lambda}^{\dagger} = \lambda^{\dagger}$ (Eq. 152).

[3] The Jacobian of the original circuit (Fig. S3*C*) has additional entries to the right of the top left diagonal block in Equation 196 that are non-zero. These non-zero entries result in the growth of synapses of y^A_E in the direction of eigencircuit *B* due to 'second-order' perturbations of recurrent synapses from eigencircuit *A* to eigencircuit *B*.

[4] Non-zero entries of the Jacobian to the right of the top left diagonal block are cancelled by the zero entries in the initial perturbation vector $\Delta\bar{\boldsymbol{w}}_v(t_0)$ (cf. Eq. 196).

In summary, recurrent synapses can stabilize or destabilize a circuit with respect to small perturbations away from a fixed point. These stabilizing and destabilizing effects are described by β-terms that depend on the specific weight configuration in the fixed point (cf. Eq. 199), which again depends on the weight norms that constrain the total synaptic weights. In the following, we consider the case when synaptic weights are tuned such that β-terms are small. In the equivalent circuit (Fig. S3*D*) this is the case when the influence of the tuning shifts of the excitatory and the inhibitory neuron balance each other[1] (cf. the first and second terms in Eqs. 202 & 204).

5.2.4 Decorrelation condition

We now consider how neurons self-organize to represent all parts of their input space instead of clustering all their tuning curves around a dominant input mode. We consider the fixed point stability of different eigencircuit configurations. In particular, we consider the case when recurrent connectivity motifs do not influence the stability of an eigencircuit. The β-terms in Equations 198 & 203 describe the change in the covariance structure of the network due to a small perturbation (cf. Sec. 5.2.3). Since we consider the stability of a single neuron in a larger network of many neurons, $N_E, N_I \gg 1$, these changes in the covariance structure are small, and the dynamics is dominated by the total attractions of the eigencircuits. Therefore, in the following, we consider β_E and β_I to be small, approximately equal to zero This can be achieved by a suitable choice of weight norms[2]. Then, all eigencircuits are marginally stable when they are equally attractive, i.e., (cf. Eqs. 198 & 203 for $\beta_{E/I} = 0$)

$$\bar{\lambda}^a = \lambda^a + \lambda^a_{\text{eig}} \overset{!}{=} \bar{\lambda}^b, \quad \forall a, b. \tag{207}$$

For homogeneous input spaces, where the feedforward attraction of all input modes is the same, i.e., $\lambda^a = \lambda^b = \lambda, \forall a, b$, the only alternative stable configuration is when all neurons are tuned to the same feedforward input mode and form a single eigencircuit. Such a configuration does not reflect the tunings of biological neural populations, where all parts of the input space are represented. To prevent such a global clustering of neural tunings, we require that the corresponding eigencircuit is unstable. When all β-terms are approximately zero, this is the case when the effective attraction of the only occupied eigencircuit, $\bar{\lambda}^*$, is smaller than the attraction of one of the $N_F - 1$ unoccupied[3] input modes, $\bar{\lambda}^\dagger = \lambda^\dagger$ (cf. Eq. 198 & 203):

$$\bar{\lambda}^* < \lambda^\dagger, \tag{208}$$

$$\Rightarrow \quad \sum_i \sigma^2_{E,i} - \sum_I \sigma^2_{I,j} + \lambda < \lambda, \tag{209}$$

$$\Rightarrow \quad N_E \sigma^2_E - N_I \sigma^2_I < 0, \tag{210}$$

$$\Rightarrow \quad \boxed{N_E \sigma^2_E < N_I \sigma^2_I}, \tag{211}$$

where σ^2_E, σ^2_I are the average variance, and N_E, N_I the total number of inhibitory and excitatory neurons. When this condition is satisfied, the only stable solution is when the effective attraction of all eigencircuits is identical. The simplest configuration where this is the case is when each eigencircuit contains the same number of excitatory and inhibitory neurons.

6 Movie captions

Movie S1: Decorrelation of feedforward tuning curves of excitatory neurons in plastic recurrent networks. Development of feedforward tuning curves of $N_E = 10$ excitatory neurons (cf. Figs. 3*A* & *B*). Synaptic weights were initialized randomly. Different color shades indicate weights of different post-synaptic neurons.

[1]Note that the term in Equation 204 that corresponds to the tuning shift of the inhibitory neuron can be positive since $\mathrm{d}y_I / \mathrm{d}(\boldsymbol{w}_{IF}{}^\top \boldsymbol{y})$ is negative when the circuit is inhibition stabilized (*6*), i.e., $w_{EE} > 1$. In that case, the first term in Equation 204 is negative, since $\mathrm{d}y_E / \mathrm{d}(\boldsymbol{w}_{IF}{}^\top \boldsymbol{y})$ is also negative (cf. Eqs.155 & 156).

[2]See Section 5.2.3 for a discussion of the case $\beta_{E/I} \neq 0$.

[3]An unoccupied input mode corresponds to $\lambda^\dagger_{\text{eig}} = 0$ (cf. Eq. 147).

Movie S2: Decorrelation of feedforward tuning curves of inhibitory neurons in plastic recurrent networks. Development of feedforward tuning curves of $N_I = 10$ inhibitory neurons (cf. Figs. 3*A* & *B*). Synaptic weights were initialized randomly. Different color shades indicate weights of different post-synaptic neurons.

References

1. E. Oja, Simplified neuron model as a principal component analyzer. *Journal of mathematical biology* (1982).
2. K. D. Miller, D. J. MacKay, The role of constraints in Hebbian learning. *Neural computation* (1994).
3. S. H. Strogatz, *Nonlinear dynamics and chaos: with applications to physics, biology, chemistry, and engineering* (CRC press, 2018).
4. T. P. Vogels *et al.*, Inhibitory plasticity balances excitation and inhibition in sensory pathways and memory networks. *Science* (2011).
5. C. Clopath *et al.*, Receptive field formation by interacting excitatory and inhibitory synaptic plasticity. *BioRxiv* (2016).
6. M. V. Tsodyks *et al.*, Paradoxical effects of external modulation of inhibitory interneurons. *Journal of neuroscience* (1997).
